# A Computational Approach for the Prediction of HIV Resistance Based on Amino Acid and Nucleotide Descriptors

**DOI:** 10.3390/molecules23112751

**Published:** 2018-10-24

**Authors:** Olga Tarasova, Nadezhda Biziukova, Dmitry Filimonov, Vladimir Poroikov

**Affiliations:** Institute of Biomedical Chemistry, Moscow 119121, Russia; nad.smol@gmail.com (N.B.); dmitry.filimonov@ibmc.msk.ru (D.F.); vladimir.poroikov@ibmc.msk.ru (V.P.)

**Keywords:** HIV-1, reverse transcriptase, protease, resistance, computational prediction, random forest

## Abstract

The high variability of the human immunodeficiency virus (HIV) is an important cause of HIV resistance to reverse transcriptase and protease inhibitors. There are many variants of HIV type 1 (HIV-1) that can be used to model sequence-resistance relationships. Machine learning methods are widely and successfully used in new drug discovery. An emerging body of data regarding the interactions of small drug-like molecules with their protein targets provides the possibility of building models on “structure-property” relationships and analyzing the performance of various machine-learning techniques. In our research, we analyze several different types of descriptors in order to predict the resistance of HIV reverse transcriptase and protease to the marketed antiretroviral drugs using the Random Forest approach. First, we represented amino acid sequences as a set of short peptide fragments, which included several amino acid residues. Second, we represented nucleotide sequences as a set of fragments, which included several nucleotides. We compared these two approaches using open data from the Stanford HIV Drug Resistance Database. We have determined the factors that modulate the performance of prediction: in particular, we observed that the prediction performance was more sensitive to certain drugs than a type of the descriptor used.

## 1. Introduction

The human immunodeficiency virus (HIV)/AIDS pandemic is one of the most important challenges facing humanity. The human immunodeficiency virus type 1 (HIV-1) leads to more than 1.8 million newly infected persons per year and causes over 1 million deaths every year. More than 36 million people are currently living with HIV [1]. HIV-1 exhibits high mutation rates and a great ability to recombination. These two factors can cause the high level of HIV-1 resistance, which leads to the necessity of HIV treatment with several combinations of antiretroviral drugs. The prediction of HIV resistance to a drug or a combination of drugs is an important issue for the development of new potent and safe antiretroviral drugs. There are several methods aimed at predicting HIV-1 resistance and disease progression based on amino acid / nucleotide sequences of HIV core proteins (reverse transcriptase, protease) [2,3,4,5,6,7,8,9,10,11,12,13,14,15,16]. Some of them have the ultimate goal of predicting HIV-1 resistance to reverse transcriptase (RT) and protease inhibitors (PR) [2,3,4,5,6,7,8,9,10,11,12,13] based on amino acid or nucleotide sequences of HIV RT and PR. In the study by Drăghici, S. and Potter, R.B. [2], neural networks were used to predict the resistance of HIV-1 to Indinavir and Saquinavir. Beerenwinkel N. et al. [3] used the Support Vector Regression based on 1980 descriptors for PR and 4401 descriptors for RT. Later, an application of the Decision Trees methodology was implemented in the Geno2Pheno web-tool [4]. Three different machine learning approaches were used to predict HIV resistance to RT and PR inhibitors [5]. Application of several different machine learning approaches to the prediction of HIV-1 resistance was also reported in References [5,6,7,8,9,10,11,12]. Some other approaches, including the study by Kierczak, M. et al. [13], are rule-based. There have also been several methods aimed at predicting HIV resistance based on the analysis of tropism [14] using various physicochemical or structural features of the HIV-1 gp120 protein. The resistance of HIV-1 to Bevirimat, a drug inhibiting the maturation of HIV-1 particles to infectious virions was predicted and analyzed in a study by Dybowski, N. et al. [15]. There are several approaches related to the HIV resistance analyses but not aimed to predict the resistance of HIV variants to a certain drug [16,17]. A lot of information on HIV sequences of various resistant and susceptible variants collected within numerous studies is available mostly for the HIV-1 core proteins, including reverse transcriptase, protease, and integrase (IN). This provides the possibility of using data sets of HIV variants with data on HIV resistance for the development and testing of new computational approaches that can be further used to analyze the resistance associated with other new mechanisms. In most cases, the preliminary alignment is used to predict the resistance of HIV variants with certain amino acid/nucleotide sequences to a particular drug. We have earlier developed a computational method for predicting the HIV resistance to antiretroviral drugs based on a set of reverse transcriptase (RT) amino acid sequences [11,12]. The method is based on the representation of an amino acid sequence of an HIV variant as a set of position-specific descriptors. Such descriptors are the combinations of a single-letter amino acid code, which position is determined using multiple alignments. In the current study, we suggest applying the nucleotide-based and peptide-based descriptors for predicting the HIV-1 resistance to reverse transcriptase (RT) and protease (PR) inhibitors. First, we represented amino acid sequences as a set of short peptide fragments that include several amino acid residues. Second, we trasformed nucleotide sequences in a set of short nucleotide fragments. It is reasonable to suggest that application of short peptides/nucleotides as descriptors allows predicting HIV-1 resistance without any preliminary alignment. Elimination of errors occuring during the alignment procedure can increase the overall accuracy of the prediction. We have earlier used [11,18] several machine-learning approaches to perform different scientific tasks and observed the better performance of the Random Forest (RF) approach in comparison with Naïve Bayes classifier, RBF networks, multilayer perceptron, and convolutional neural networks. Moreover, we have shown that the RF approach is less sensitive to imbalanced datasets comparing with artificial neural networks. Thus, we used the RF approach since, according to our earlier studies, it yielded the best performance for both balanced and imbalanced datasets [11,18].

## 2. Results

### Results of the Prediction Based on the Peptide-Based Descriptors and Nucleotide Descriptors

The number of peptide and nucleotide descriptors was higher than the number of instances. To reduce the number of descriptors, we selected 500 most common nucleotide descriptors that had a frequency of occurrence higher than 30. We also selected 130 most common descriptors from the short peptides with an occurrence frequency of over 100 among all the amino acids sequences.

We calculated the metrics of classification, including sensitivity (Sns), specificity (Spc), precision (positive predictive value: PPV), the Matthews correlation coefficient (MCC), and the area under the Receiver operating characteristics (ROC) curve (AUC). We predicted whether a particular sequence belongs to HIV variants resistant to RT or PR inhibitors. The accuracy of prediction (Table 1) is calculated as a result of 5-fold cross validation. The average sensitivities of the models for prediction of HIV-1 resistance are 0.94 (short peptides as descriptors) and 0.92 (nucleotide sequences as descriptors), while the average specificities are 0.75 (peptide descriptors) and 0.78 (nucleotide-based descriptors). The balanced accuracies of the resistance prediction to RT inhibitors are 0.84 (peptide-based descriptors) and 0.85 (nucleotide-based descriptors). The balanced accuracies of the resistance prediction to PR inhibitors are about 0.86 (peptide-based descriptors) and 0.85 (nucleotide-based descriptors).

## 3. Discussion

### 3.1. Comparison of the Accuracy Obtained Using Particular Types of Descriptors

The results of the prediction gave us an opportunity to compare the usage of short peptides and short nucleotides as descriptors. It is clear that, in general, the RT nucleotide descriptors yield a better performance compared to the peptide descriptors. However, for particular drugs—including Lamivudine (3TC), Abacavir (ABC), and Tenofovir (TDF)—the situation was different. The fact that, in general, nucleotide descriptors yield the better performance has confirmed our hypothesis that they are more descriptive than peptide ones. As to the resistance to PR inhibitors, in general, the performance based on the nucleotide descriptors was not higher in comparison to that based on the short peptides; the performance of prediction was more dependent on the drug than on the type of the descriptors. To explain our findings, we compared the value reflecting the ratio of the number of resistant to the number of susceptible variants in the sets of protease and reverse transcriptase sequences, respectively. The ratio *r* of resistant to susceptible variants was 0.49 ± 0.56 (average ± standard deviation (SD)) for the set of RT amino acid sequences (the PhenosenseDS-RT set; see the Materials and Methods Section). The same value, *r*, was 2.73 ± 2.26 for the nucleotide sequences of RT. The value *r* was 1.61 ± 0.87 for the nucleotide sequence of PR. Therefore, in general, the RT dataset was more imbalanced and biased towards the resistant class. It might be a factor that influences a higher performance of prediction for the RT dataset in comparison to the PR one. Nevertheless, nucleotide descriptors are largely preferable for the prediction of HIV-1 resistance. They do not require any processing procedures (i.e., the translation of a nucleotide sequence to amino acid sequence) or pre-alignment and can be used for prediction using various machine learning approaches. In addition, we did not use the complete set of peptide/nucleotide descriptors generated from the training sets in order to reduce their number and avoid an incorrect ratio between the number of instances and the number of descriptors. Although we selected only a few descriptors, the performance of prediction was still reasonable. It confirmed that we chose correct procedure of descriptors selection.

### 3.2. Application of the Model to Predict Human Immunodeficiency Virus Type 1 (HIV-1) Resistance to Protease Inhibitors

We tested whether our approach can recognize resistant variants if the sequence of Protease of the corresponding variant (or isolate) contains wild-type residues in the major positions affecting the resistance. We created data set HiglyResPR, which contained the wild-type residues in the major drug resistance positions: there were 449 instances in HiglyResPR. Then we excluded them from the main set (consisting of 900 istances) and used in as an external test set. The results of prediction for the HiglyResPR are given in Table 2.

Table 2 shows that an alignment-independent approach can be used to predict whether a particular amino acid sequence belongs to a class of resistant variants with good performance for azatanavir (ATV), indinavir (IDV), lopinavir (LPV), nelfinavir (NFV), saquinavir (SQV). However, the results are not that impressive for fosamprenavir (FPV), tipranavir (TPV), darunavir (DRV). This might be associated with the mutation patterns of particular sequences. Nevertheless, we think that the descriptors that we generated, where it was possible, for the whole sequence can potentially lead to a high number of instances. Such a significant quantity of instances can be correctly analyzed, even if such sequences do not contain any mutations in the major drug resistance positions.

### 3.3. Comparison with the Earlier Developed Approaches

The most recent approaches aimed at predicting HIV resistance to RT and/or PR inhibitors are briefly reviewed in References [19,20]. There are several studies related to the HIV resistance predictions [2,3,4,5,6,7,8,9,10,11,12,13,14,15]. However, References [13,14,15,16] do not provide a prediction of HIV resistance to protease and reverse transcriptase inhibitors; they are aimed either on the prediction of tropism, the usage of a co-receptor, and HIV maturation inhibition [13,14,15] or prediction of the HIV inhibitors activity against mutated strains. We compared the performances of prediction using our method with the earlier developed machine learning approaches [3,4], where decision trees were used as the main computational method. For comparison purposes, we chose two studies that used metrics similar to ours, including sensitivity, specificity, and balanced accuracy. Thus, we were able to compare the performances of the predictions in a comparatively straightforward way. Assuming that we can calculate the balanced accuracy (BA) of prediction if we know the sensitivity and specificity (BA = (Sensitivity + Specificity)/2), we decided to compare the BA values obtained by our method and those achieved by other methods. We compared the accuracy of the prediction obtained by our method using descriptors based on short nucleotides. The results are given in Table 3. The AUC values were also given and available for comparison.

In the study by Beerenwinkel, N. et al. [3], the authors used 471 pre-aligned sequences of the *pol* gene. The decision tree model was built for each drug. In the study by Rhee, S.-Y. et al. [5], the authors applied five different statistical methods to classify the isolates as susceptibly/intermediately/highly resistant to a certain drugs. However, it is unclear whether the preliminary alignment was used in this study. For a direct comparison, we used only the parameters of performance obtained by the decision tree algorithm.

We also compared the results of the prediction obtained by our approach with other machine learning methods aimed at predicting the resistance to RT and PR inhibitors [7,10]. Table 3 shows the comparison of the prediction performance. For the most common reverse transcriptase inhibitors, such as abacavir (ABC) and nevirapine (NVP), the prediction performance obtained by our approach was the same as that which was reported in the study by Beerenwinkel et al., 2002 [3]. For zidovudine (AZT), the prediction performance achieved by our method was lower than in Reference [3], but it was higher than that reported in the study by Rhee S.Y. et al. [3]. In our approach, the prediction performance for stavudine (D4T) and didanozine (DDI) was much higher whereas, for IDV, NFV, SQV protease inhibitors this value was higher or the same. Hence, this simple method based on the use of short nucleotide descriptors and the Random Forest as a modeling method can be successfully applied for prediction of HIV variants resistance to reverse transcriptase and protease inhibitors.

The AUC values were higher for lamivudine (3TC), zidovudine (AZT), stavudine (D4T), didanosine (DDI), efavirenz (EFV), tenofovir (TDF), atazanavir (ATV), and indinavir (IDV) in comparison with those reported in Reference [7]. The number of misclassification errors, in general, was higher for the predictions made by our approach for RT inhibitors and they are lower for PR inhibitors compared to Reference [10]. We would like to emphasize that the usage of misclassification errors without any additional metrics of accuracy is not enough for a proper comparison of accuracy since the number of errors does not include the metrics reflecting the recognition of each class, i.e., “resistant” and “susceptible”. In this case, the number of classification errors might be very low despite the fact that one class—for example, the “susceptible”one—is prevalent in the test set and has a very good prediction, while another class cannot be recognized well.

The results of the prediction reported in the study by Murray et al. [6] are only given for tenofovir (TDF), so the comparison of these results with those obtained by our method with this study will not provide any significant differences [6]. Additionally, we could not directly compare our approach with that of Reference [8] since those models were obtained using another test system (Antivirogram). We showed earlier that the Phenosense data provided a higher accuracy of prediction [19]. Comparing to our approaches developed earlier [11,12], the performance of prediction was higher for several drugs, including AZT, TDF, EFV, and DDI.

For this purpose, we created a training set and a test set of PR sequences. As a training set, we used data on HIV PR sequences and the corresponding data on resistance uploaded to the Stanford HIV Drug Resistance Database (StDB) no later than 2006. 

It consisted of 448 sequences with resistance data against 8 HIV protease inhibitors. For the test set, we used all sequences uploaded to StDB after 2006. This set contained 51 sequences. So, we attempted to model a kind of prospective validation. Both sets with sequences, resistance data and the set of descriptors are available in Supplementary Materials (TrainingPR, ExtTestSetPR). Further, we would have liked to compare the results of prediction with other similar approaches. Unfortunately, we were able to draw a comparison only with the approach by Beerenwinkel, N. et al., 2003 [3] since the Geno2Pheno web-application is available just for that approach [4].

**Figure 1:**
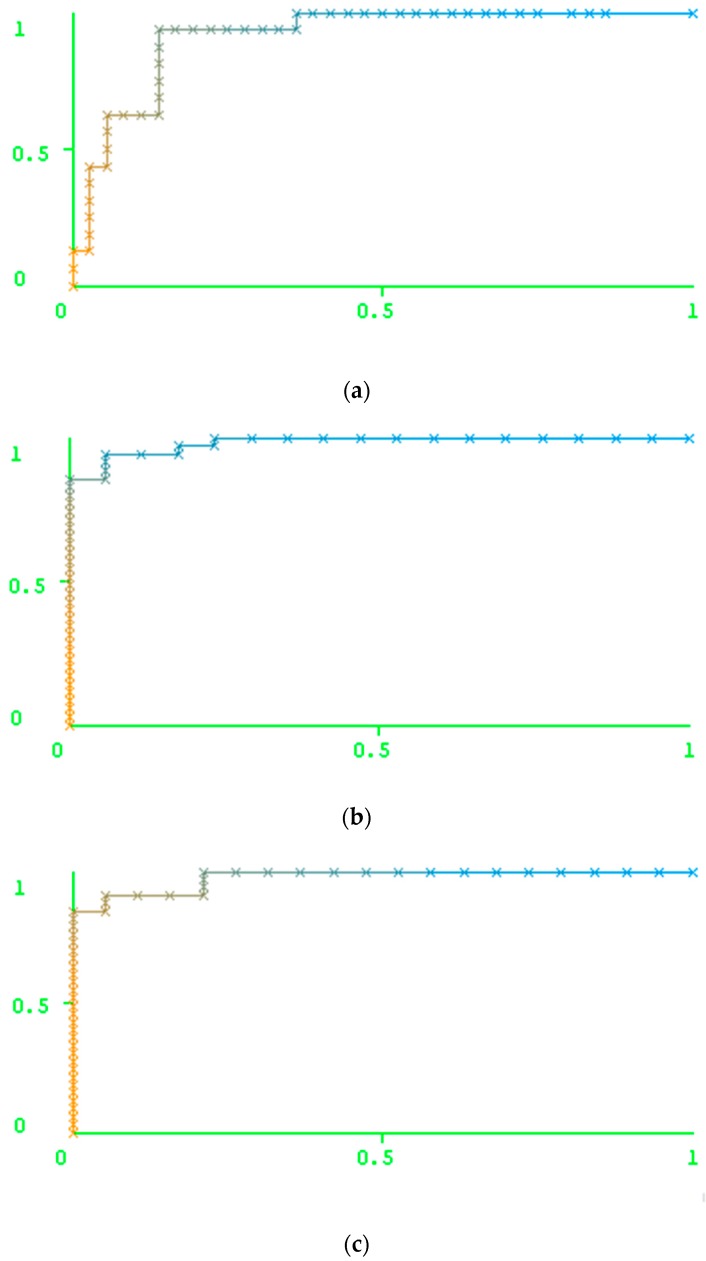

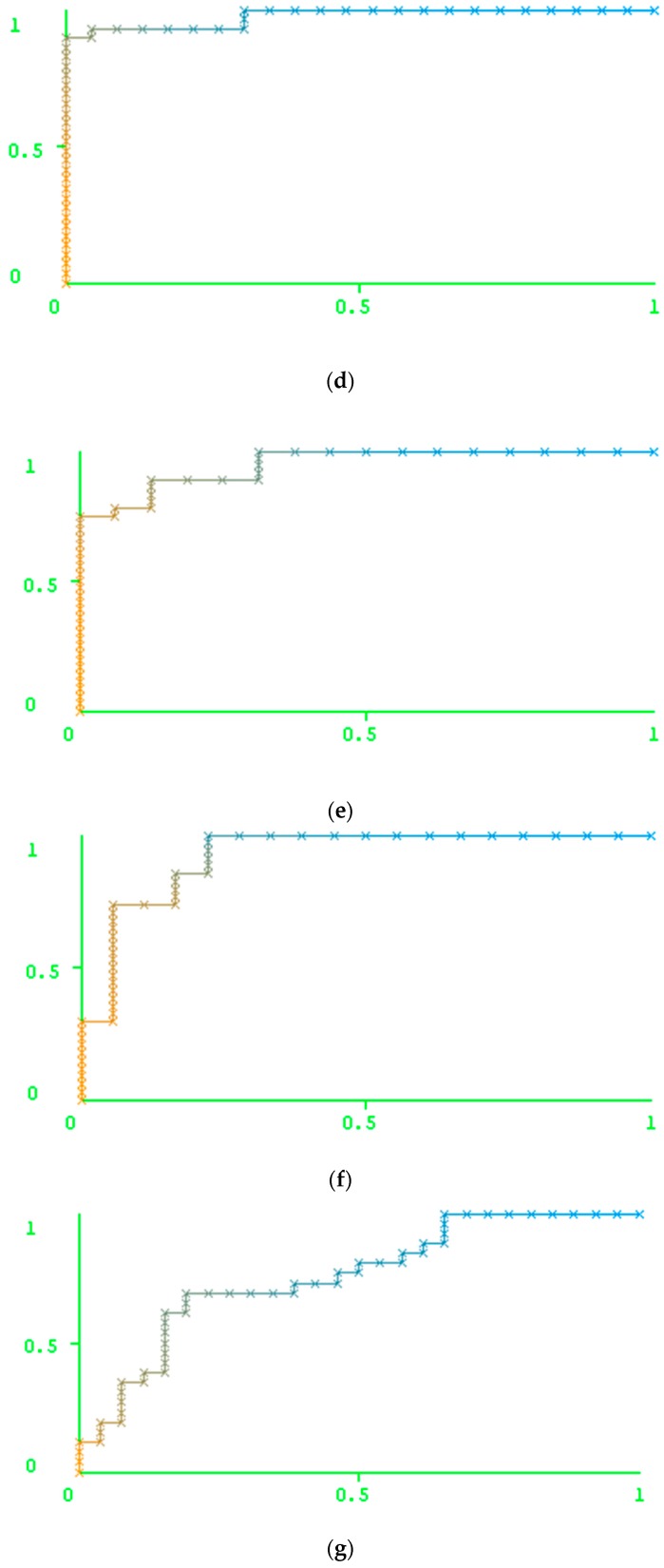

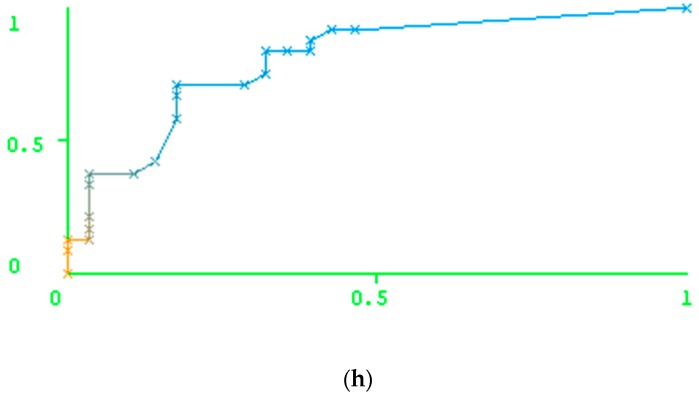
The reciever operating characteristics (ROC) curves obtained for the results of the human immunodeficiency virus type 1 (HIV-1) resistance prediction to eight protease inhibitors: (**a**) fosamprenavir (FPV), (**b**) azatanavir (ATV), (**c**) indinavir (IDV), (**d**) lopinavir (LPV), (**e**) nelfinavir (NFV), (**f**) saquinavir (SQV), (**g**) tipranavir (TPV), (**h**) darunavir (DRV). X axis: False Positive Rate (FPR); Y axis: True Positive Rate (TPR); Color represents Weka threshold value set to get the value of FPR/YPR for each point. For blue color, threshold value is close to zero (“0”), for orange color, value it is close to one (“1”).

We used the training set, created the models, made predictions for the test set using our method and then uploaded the sequences of the test set to the Geno2Pheno web server. The results obtained in Geno2Pheno made it possible to calculate the values of Sensitivity, Specificity, Precision (PPV), Recall (Rec) and Balanced accuracy (BA). The same characteristics were obtained in Weka 3.8 using the set of descriptors and RF model (Table 4). We also reported the areas under the ROC curve for each model and the AUC values themselves (Figure 1). The data used for the ROC curve plotting are also available in the Supplementary Materials. We suppose that these data can be used for the comparison of our approach with any other method.

As Table 4 shows, the results of the prediction made by Geno2Pheno are better than the prediction results obtained by our method for the resistance against ATV and DRV, while, for IDV, LPV, NFV, and SQV, the results of the prediction obtained using our method outperform the results of the prediction using Geno2Pheno. The accuracy of prediction was similar for the cases of FPV and TPV.

As Figure 1 shows, the performance of the prediction is high for indinavir and nelfinavir, while this value is not as high for tipranavir (TPV) and darunavir (DRV). In particular, one can notice a comparatively low number of resistant variants against the latter two drugs in the test set. In this case, the results might be explained by the peculiarities of the test set creation. However, the 5-fold validation also did not reveal the high accuracy of prediction for these two drugs. Therefore, the low accuracy of prediction for these two drugs can be explained by a weak relationship between the features of the amino acid and the nucleotide sequences and the level of resistance of the corresponding variants to the PR inhibitors.

In general, the 5-fold validation and the results of the prediction for the test set allowed for good recognition of resistant HIV variants against the following drugs: zidovudine (AZT), stavudine (D4T), efavirenz (EFV), etravirine (ETR), nevirapine (NVP), indinavir (IDV), lopinavir (LPV), nelfinavir (NFV), and saquinavir (SQV).

## 4. Materials and Methods

### 4.1. Datasets

For our models, we used 1985 amino acid sequences of HIV reverse transcriptase and 2109 amino acid sequences of protease from the Stanford HIV Drug Resistance Database [21]. We downloaded the data produced by the Phenosense test system (PhenosenseDS set). We chose the PhenosenseDS set because it contains highly consistent data on HIV RT amino acid sequences compared to the other test systems [19]. StDB contains data on the resistance of HIV variants to ten RT inhibitors used in clinical practice (the abbreviations are given in brackets): lamivudine (3TC), abacavir (ABC), zidovudine (AZT), stavudine (D4T), didanosine (DDI), efavirenz (EFV), etravirine (ETR), nevirapine (NVP), rilpivirine (RPV), and tenofovir (TDF). The data on the resistance to protease inhibitors are available for eight drugs: fosamprenavir (FPV), azatanavir (ATV), indinavir (IDV), lopinavir (LPV), nelfinavir (NFV), saquinavir (SQV), tipranavir (TPV), and darunavir (DRV). A particular RT or PR sequence characterizes by the fold ratio (FR) value, estimated for each antiretroviral drug. The FR value is calculated as the ratio (IC_50_/IC_50__WT): the IC_50_ value of a drug for an HIV RT variant is divided by the IC_50__WT of this drug for the wild-type of RT. The data on the amino acid sequences of RT and PR with the FR values against a certain drug are collected in the PhenosenseDS dataset. To divide the set of HIV-1 isolates into the resistant and susceptible variants, we used the clinical cutoff of FR, which is the value calculated based on the clinical response data from treatment-experienced patients (for details, see References [11,22]). The numbers of susceptible/resistant variants for each drug are given in Table 5. Approximately 38% of the amino acid sequences from PhenosenseDS contained two to four symbols in a single position corresponding to a single-letter amino acid code (“mixture”).

We calculated the frequency of occurrence of each amino acid residue from the “mixture” in the set of resistant variants of HIV-1. We put the most frequent amino acid residues in the resistant variants in the positions where any “mixture” had occurred.

We prepared the data for the prediction of HIV resistance to RT and PR inhibitors based on amino acid sequences since there were a lot of open data on the relationship between amino acid sequences and the resistance to HIV RT and PR inhibitors. Additionally, we would like to demonstrate the ability of our approach to deal with the descriptors of nucleotide sequences due to several reasons. The first reason is so that it can handle nucleotide polymorphisms and have the possibility of taking frameshift mutations into account, which may be unrecognized when the DNA-protein translation algorithm is applied before the classification procedure. The second reason is the possibility of dealing with the nucleotide sequences without any preprocessing since the initial material taken from a patient is isolate that contain nucleotide sequences. However, most available data do not contain a lot of information on the relationship between nucleotide sequences and amino acid sequences. Therefore, we first used short peptides as descriptors to prove the effectiveness of our method when using a comparatively big dataset and then we prepared a dataset of nucleotide descriptors and tested the applicability of our method based on it.

Unfortunately, the nucleotide sequences were not available for all isolates of PhenosenseDS. Nevertheless, we collected the nucleotide sequences by overlapping the isolate identifiers of PhenosenseDS and the data regarding the genotype-treatment relationships from StDB, which, in turn, contained the nucleotide sequences. The data on nucleotide sequences were obtained for 683 variants of RT (PhenosenseNDS-RT) and for 877 variants of PR (PhenosenseNDS-PR). The details of the number of susceptible and resistant variants of this dataset are given in Table 6. The total number of instances in each dataset is different since each dataset contains several samples without any data on resistance.

### 4.2. Descriptors

In our work, we used two types of descriptors and compared the performance of the predictions for these two types of descriptors. We used fragments of nucleotide sequences as descriptors. Each descriptor was generated as a set of 24 nucleotides centered by positions, with each one shifted by 9 nucleotides from the previous centered position. We carried out a few experiments to maximize the description of the nucleotide sequence and to minimize the number of descriptors so that the length of the nucleotide fragments and the shift of the centered position were determined as mentioned earlier.

In total, 4187 short peptides were generated (the average number was 240 ± 28 descriptors per sequence) for HIV-1 reverse transcriptase (PeptRT descriptors set) and 4789 descriptors for HIV-1 protease (PeptPR set).

The number of generated short nucleotide sequences was 15687 descriptors (the average number was 180 ± 26 descriptors per sequence) for HIV-1 RT (NuclRT set) and 5461 for HIV-1 PR (NuclPR descriptors set).

### 4.3. Algorithm and Validation

We generated a set of short peptides and short nucleotides from the sequences of training sets. Then we selected the most common short peptide and nucleotide-based descriptors. The number of selected nucleotide-based descriptors was 500 and the number of short peptide descriptors was 130.

Further, we prepared a set of binary descriptors based on the generated set of peptides and nucleotides. For each amino acid or nucleotide sequence, we designed a set of “0” and “1”, where “0” was added to the set if the descriptor of the sequence considered could not be found in the total set of descriptors and “1” if, conversely. So, for each amino acid or nucleotide sequence, we prepared a set of “0” and “1” of a fixed size. The size of nucleotide-based descriptors was 500, and the size of peptide-based descriptors was 130. We used these descriptors for prediction performed using the Random Forest algorithm implemented in the Weka 3 software. We chose this algorithm because it had yielded the best performance for both the balanced and imbalanced datasets [8]. The parameters of the algorithm were the following: the number of “seeds” = 1; the number of “trees” (iterations) = 1000; the number of examples utilized in one iteration (batch size) = 1000; the other parameters were used as the default ones. A 5-fold cross-validation procedure was applied to calculate the performance of the prediction.

## 5. Conclusions

In our study, we demonstrated the use of the Random Forest approach for the modeling of HIV-1 resistance to reverse transcriptase and protease inhibitors based on the descriptors represented by small peptide and/or nucleotide fragments. We observed a reasonable performance of prediction, depending on the specific drug and specific protein (reverse transcriptase, protease). In general, the prediction performance for the resistance to RT was better compared to PR. We also showed that the nucleotide descriptors generated from several positions located at an equal distance from each other, as well as the descriptors based on short peptides, could be used for the prediction. Comparison of our approach to the earlier developed ones gave the possibility of determining several cases, where the prediction performance was better than that obtained by the earlier approaches. Generally, the method proposed provides similar or higher accuracy of prediction compared to other methods and can be used for prediction of HIV resistance to reverse transcriptase and protease inhibitors.

## Figures and Tables

**Table 1:** The performance of the prediction of the human immunodeficiency virus type 1 (HIV-1) resistance to reverse transcriptase (RT) and protease inhibitors (PR) inhibitors.

Drug	Peptide Descriptors					Nucleotide Descriptors				
**Reverse Transcriptase Inhibitors**										
	Sns	Spc	PPV	MCC	AUC	Sns	Spc	PPV	MCC	AUC
3TC	0.98	0.68	0.95	0.74	0.96	0.99	0.63	0.93	0.75	0.97
ABC	0.98	0.74	0.94	0.70	0.91	0.98	0.72	0.89	0.72	0.92
AZT	0.91	0.76	0.89	0.70	0.93	0.93	0.78	0.85	0.72	0.94
D4T	0.93	0.80	0.84	0.70	0.91	0.94	0.79	0.85	0.70	0.91
DDI	0.90	0.74	0.92	0.72	0.94	0.98	0.65	0.89	0.69	0.91
EFV	0.88	0.76	0.91	0.70	0.91	0.87	0.89	0.81	0.69	0.91
ETR	0.92	0.74	0.94	0.70	0.92	0.87	0.99	0.88	0.78	0.93
NVP	0.96	0.80	0.90	0.72	0.94	0.92	0.89	0.87	0.77	0.96
TDF	0.88	0.69	0.86	0.70	0.91	0.60	0.95	0.91	0.69	0.97
Avg *	0.93	0.75	0.91	0.71	0.93	0.90	0.81	0.88	0.72	0.94
**Protease Inhibitors**										
	Sns	Spc	PPV	MCC	AUC	Sns	Spc	PPV	MCC	AUC
FPV	0.96	0.68	0.89	0.69	0.91	0.94	0.61	0.88	0.69	0.91
ATV	0.98	0.68	0.90	0.70	0.92	0.97	0.61	0.90	0.70	0.91
IDV	0.97	0.74	0.89	0.72	0.93	0.98	0.86	0.92	0.77	0.96
LPV	0.96	0.76	0.86	0.70	0.92	0.92	0.71	0.87	0.74	0.93
NFV	0.96	0.89	0.88	0.77	0.96	0.95	0.84	0.91	0.77	0.94
SQV	0.94	0.79	0.89	0.75	0.93	0.96	0.83	0.91	0.77	0.94
TPV	0.96	0.74	0.86	0.72	0.94	0.92	0.64	0.89	0.70	0.92
DRV	0.96	0.78	0.88	0.72	0.91	0.98	0.82	0.84	0.72	0.92
Avg	0.96	0.76	0.88	0.72	0.93	0.95	0.74	0.89	0.73	0.93

* Average value for the set of drugs. Abbreviations are as follows: lamivudine (3TC), abacavir (ABC), zidovudine (AZT), stavudine (D4T), didanosine (DDI), efavirenz (EFV), etravirine (ETR), nevirapine (NVP), rilpivirine (RPV), and tenofovir (TDF). The data on the resistance to protease inhibitors are available for the following eight drugs: fosamprenavir (FPV), azatanavir (ATV), indinavir (IDV), lopinavir (LPV), nelfinavir (NFV), saquinavir (SQV), tipranavir (TPV), and darunavir (DRV).

**Table 2:** The results of the prediction performance for the Protease sequences with wild-type residues in the major drug resistance position (HiglyResPR dataset).

Drug	Nr *	Ns	Sns	Spc	PPV	MCC	AUC
FPV	65	340	0.43	0.94	0.51	0.40	0.91
ATV	96	271	0.84	0.96	0.82	0.81	0.98
IDV	214	184	0.78	0.76	0.69	0.90	0.92
LPV	145	142	0.79	0.94	0.77	0.70	0.93
NFV	248	168	0.94	0.98	0.88	0.96	0.97
SQV	192	223	0.83	0.80	0.80	0.71	0.94
TPV	78	118	0.52	0.96	0.50	0.50	0.96
DRV	64	124	0.65	0.94	0.59	0.76	0.96
Avg			0.725	0.91	0.70	0.72	0.95

* Nr is the number of resistant instances in the dataset; Ns is the number of sensitive instances in the dataset.

**Table 3:** The comparison of the prediction performance of our approach and some earlier developed approaches.

Drug	BA (Our)	BA [3]	BA [6]	AUC (Our)	AUC [7]	MCR * (Our)	MCR [10]
3TC	0.81	0.89	0.9	0.97	0.94	7.29	3.87
ABC	0.85	0.85	0.69	0.92	0.92	6.8	6.53
AZT	0.86	0.89	0.70	0.94	0.91	13.96	36.19
D4T	0.87	0.75	0.76	0.94	0.90	10.01	7.31
DDI	0.82	0.68	0.75	0.91	0.85	10.90	8.05
EFV	0.88	0.902	0.84	0.96	0.93	18.08	16.08
ETR	0.93	N/D	N/D	0.93	N/D	10.01	6.58
NVP	0.91	0.91	0.91	0.94	0.92	12.7	24.87
RPV	N/D	0.89	N/D	N/D	N/D	N/D	1.55
TDF	0.78	N/D	N/D	0.92	0.83	12.3	5.39
FPV	0.78	N/D	N/D	0.92	N/D	15.8	16.08
ATV	0.79	0.87	0.71	0.93	0.93	26.2	26.69
IDV	0.92	0.89	0.75	0.98	0.97	8.2	34.29
LPV	0.82	N/D	0.77	0.94	0.96	23.8	9.79
NFV	0.90	0.89	0.76	0.96	0.94	7.15	25.23
SQV	0.90	0.88	0.75	0.96	0.96	11.15	30.37
TPV	0.78	N/D	N/D	0.87	N/D	4.77	9.07
DRV	0.79	N/D	N/D	0.92	N/D	2.38	2.98
Avg	0.854	0.857	0.78	0.94	0.92	11.85	15.05

* Misclassification Rate: calculated as non-concordant pairs between resistant/susceptible classes, obtained experimentally (Phenosense test system) and classes by prediction (the percentage); N/D: no data available.

**Table 4:** The comparison of the prediction performance of our approach and the earlier developed approach.

Drug	Random Forest (Our)						Decision Trees [3,4]			R *
	Sns	Spc	PPV	BA	AUC	Sns	Spc	PPV	BA	
FPV	0.41	0.97	0.89	0.69	0.94	0.99	0.34	0.52	0.675	32
ATV	0.69	0.99	0.99	0.84	0.97	0.86	0.72	0.70	0.91	89
IDV	0.99	0.96	0.92	0.98	0.98	0.91	0.66	0.89	0.785	190
LPV	0.99	0.83	0.92	0.92	0.92	0.90	0.90	0.99	0.90	96
NFV	0.97	0.97	0.95	0.99	0.97	0.86	0.50	0.86	0.68	215
SQV	0.91	0.82	0.91	0.92	0.96	0.83	0.49	0.82	0.66	162
TPV	0.10	0.99	0.09	0.76	0.78	0.54	0.89	0.53	0.715	16
DRV	0.20	0.99	0.16	0.76	0.80	0.75	0.88	0.75	0.815	24

* R: number of sequences of resistant variants.

**Table 5:** The number of amino acid sequences considered to belong to the resistant and susceptible variants.

Drug	FR *	Total	Susceptible	Resistant
3TC	1.5	1727	635	1092
ABC	4.5	1655	1494	161
AZT	2.2	1747	1002	745
D4T	1.7	1755	1632	123
DDI	1.7	1756	1034	722
EFV	2.5	1378	1278	100
ETR	2.9	1836	1754	82
NVP	2.5	1844	962	882
RPV **	N/D	N/D	N/D	N/D
TDF	1.5	1378	1218	160
FPV	20	1965	1614	351
ATV	2.2	1309	714	595
IDV	2.4	2007	1036	971
LPV	6.7	1693	917	717
NFV	3.6	2102	954	1148
SQV	2.07	2012	925	1087
TPV	1.2	1060	477	583
DRV	5.5	734	147	582

* FR corresponding to the clinical cut-off. ** N/D: no data available.

**Table 6:** The number of nucleotide sequences considered to belong to the resistant and susceptible variants.

Drug	FR *	Total	Susceptible	Resistant
3TC	1.5	720	74	646
ABC	4.5	740	181	563
AZT	2.2	718	272	446
D4T	1.7	723	258	465
DDI	1.7	720	123	597
EFV	2.5	744	353	391
ETR	2.9	193	57	136
NVP	2.5	756	316	440
RPV	N/D	N/D	N/D	N/D
TDF	1.5	423	234	189
FPV	20	774	666	108
ATV	2.2	352	150	202
IDV	2.4	795	367	428
LPV	6.7	614	332	282
NFV	3.6	833	342	491
SQV	2.07	827	445	382
TPV	1.2	196	101	96
DRV	5.5	165	139	26

* FR corresponding to the clinical cut-off. ** N/D: no data available.

## Supplementary Materials

The following are available online. The processed data sets including the data in nucleotide sequences and the data reflecting belonging to a class “resistant” and “susceptible” used in this study are available as the text files. The data sets of external test sets HiglyResPR, TrainingPR, ExtTestSetPR are given in Supplemetary materials.
