# Supplementary material for: A Computational Approach for the Prediction of HIV Resistance Based on Amino Acid and Nucleotide Descriptors
Source: Molecules. 2018 Oct 24;23(11):2751. doi: 10.3390/molecules23112751 (PMC6278491; doi:10.3390/molecules23112751)
Supplement: Supplementary file 1 [file molecules-23-02751-s001.zip › Geno2Pheno-test-set-Predictions-PDF/geno2pheno-results/11.pdf]

geno2pheno®

phenotype prediction from genotype (version 3.4)

I. General information

Patient:		Study Id:	
Birth date:		Viral load:	
Sample received:		Sample collected:	
Sample ID:	11	Predicted subtype:	C (100%)
Sample type:		Report date:	October 6, 2018
Physician:		Reported by:	

II. Substitutions

(relative to the reference strain HXB2)

Protease:	L10Y, I15V, K20T, E35G, M36I, S37D, R41K, M46I, F53L, L63V, I64V, H69K, T74A, N88S, L89M, I93L
Reverse transcriptase:	

III. Phenotype prediction

Drug	Resistance Factor RF (*)	z-score	Scored Positions (**)
ZDV	0	0	
ddI	0	0	
d4T	0	0	
3TC	0	0	
ABC	0	0	
TDF	0	0	
NVP	0	0	
EFV	0	0	
ETR (***)	Susceptible		
RPV (***)	Susceptible		
SQV	7.36	5.658	48G 73G 90L 84I 54I 11V 95C 53L 26T 71A 80T 35G 34E 10Y 63V
IDV	8.232	5.167	54I 82V 29D 73G 21E 65E 84I 46I 11V 71A 85I 30D 90L 88S 89M
NFV	20.483	6.545	54I 30D 82V 97L 73G 68G 90L 71A 46I 88S 31T 75V 84I 5L 89M
APV	1.528	0.988	54I 76L 50I 84I 32V 88S 46I 85I 37D 22A 47I 13I 82V 89M 97L
LPV	4.136	4.236	54I 82V 84I 50I 46I 76L 22A 71A 7Q 10Y 24L 25D 37D 47I 92Q
TPV	0.447	-1.77	46I 48G 84I 33L 54I 47I 89M 71A 72I 91T 69K 15V 90L 82V 58Q
DRV	0.991	-0.222	47I 84I 54I 33L 76L 43K 73G 71A 37D 65E 93L 89M 48G 7Q
ATV	6.879	5.328	54I 48G 73G 84I 82V 4T 88S 71A 90L 35G 7Q 24L 76L 45K 32V

(*) based on LIBSVM, Copyright (c) 2000, Chih-Chung Chang and Chih-Jen Lin

(**) Positions are ordered according to their impact on the phenotype prediction. Differences with respect to HXB2 are underlined. Positions shown in red and in green contribute to an increase or decrease in resistance, repectively. At most 15 positions are shown for each drug.

(***) Resistance predictions and scored mutations for ETR and RPV were performed with rules-based drug-resistance interpretation models by HIV-GRADE (http://www.hiv-grade.de)

geno2pheno®

phenotype prediction from genotype (version 3.4)

IV. Interpretation

Patient:	Birth date:	Sampling date:
Current therapy:		Viral load:

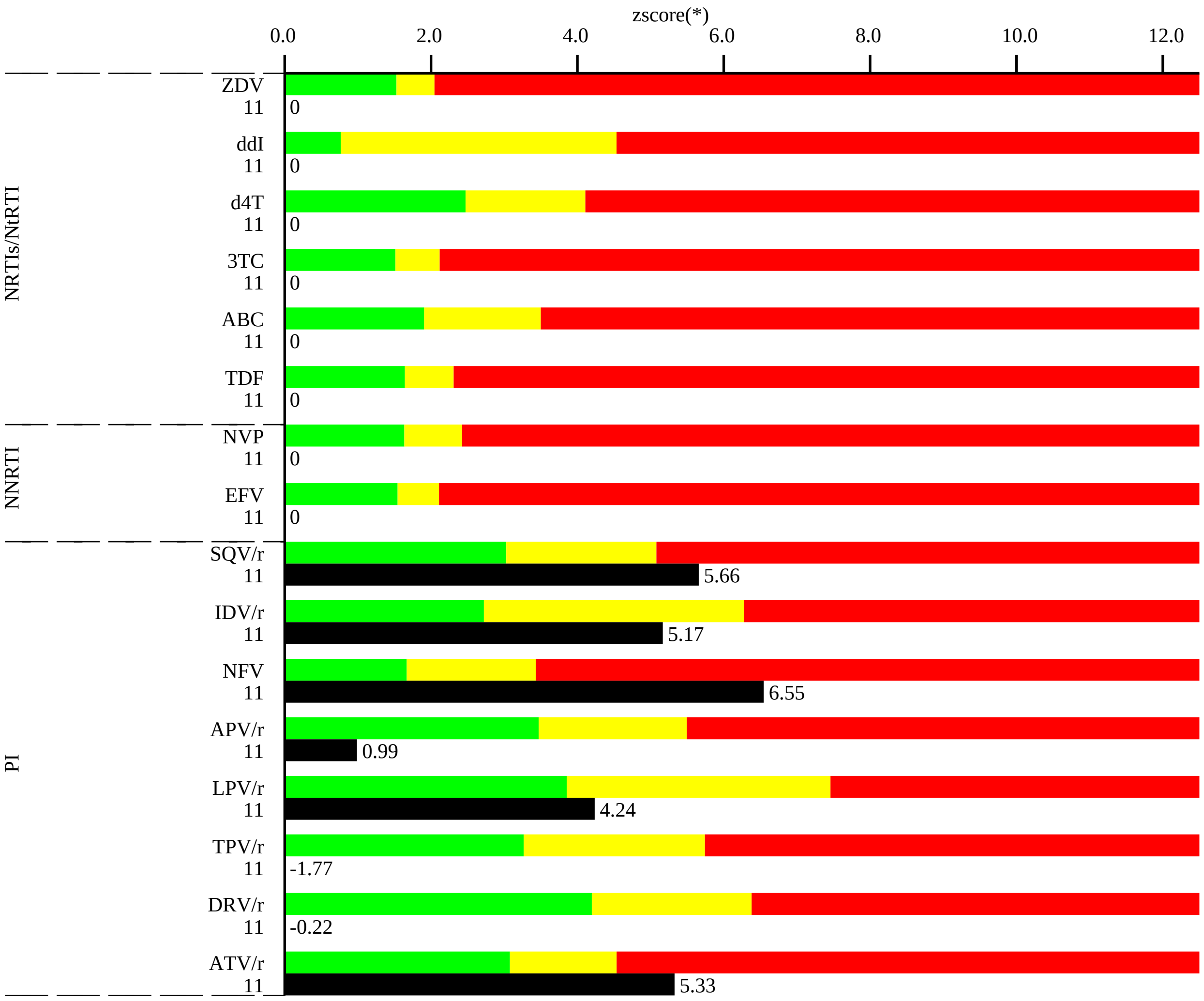

susceptible intermediate resistant

(*)number of standard deviations above mean of drug naive patients. Negative z-scores may indicate hypersusceptibility.

NRTIs/NtRTI:

NNRTIs:

PIs:

Previous genotypes:

date

signature
