# Supplementary material for: A Computational Approach for the Prediction of HIV Resistance Based on Amino Acid and Nucleotide Descriptors
Source: Molecules. 2018 Oct 24;23(11):2751. doi: 10.3390/molecules23112751 (PMC6278491; doi:10.3390/molecules23112751)
Supplement: Supplementary file 1 [file molecules-23-02751-s001.zip › Geno2Pheno-test-set-Predictions-PDF/geno2pheno-results/12.pdf]

geno2pheno®

phenotype prediction from genotype (version 3.4)

I. General information

Patient:	Study Id:
Birth date:	Viral load:
Sample received:	Sample collected:
Sample ID: 12	Predicted subtype: B (100%)
Sample type:	Report date: October 6, 2018
Physician:	Reported by:

II. Substitutions

(relative to the reference strain HXB2)

Protease:	L19I, S37T, M46I/M, I50L, Q58E, L63P, I64L, A71V, I72I/R, V77I, V82A/V, N88N/S
Reverse transcriptase:	

III. Phenotype prediction

Drug	Resistance Factor RF (*)	z-score	Scored Positions (**)
ZDV	0	0	
ddI	0	0	
d4T	0	0	
3TC	0	0	
ABC	0	0	
TDF	0	0	
NVP	0	0	
EFV	0	0	
ETR (***)	Susceptible		
RPV (***)	Susceptible		
SQV	3.456	3.46	48G 73G 90L 84I 54I 11V 74T 53F 95C 26T 80T 34E 47I 97L 24L
IDV	14.904	6.773	54I 29D 73G 21E 65E 84I 46I 11V 85I 30D 90L 88S 78G 98N 76L
NFV	23.858	6.904	54I 30D 97L 20K 73G 68G 90L 36M 46I 88S 31T 75V 74T 84I 5L
APV	5.757	5.186	54I 76L 84I 32V 46I 85I 22A 63P 47I 13I 97L 45K 58E 21E 29D
LPV	8.329	6.262	54I 84I 46I 76L 10L 22A 7Q 63P 24L 20K 25D 82A 47I 92Q 90L
TPV	2.656	2.093	69H 48G 84I 33L 54I 47I 15I 91T 71V 20K 90L 82V 74T 46M 88N
DRV	1.862	1.475	47I 84I 54I 33L 76L 74T 43K 73G 36M 65E 48G 93I 10L 15I
ATV	13.125	7.327	54I 48G 73G 84I 82A 4T 88S 90L 7Q 36M 20K 24L 76L 45K 32V

(*) based on LIBSVM, Copyright (c) 2000, Chih-Chung Chang and Chih-Jen Lin

(**) Positions are ordered according to their impact on the phenotype prediction. Differences with respect to HXB2 are underlined. Positions shown in red and in green contribute to an increase or decrease in resistance, repectively. At most 15 positions are shown for each drug.

(***) Resistance predictions and scored mutations for ETR and RPV were performed with rules-based drug-resistance interpretation models by HIV-GRADE (http://www.hiv-grade.de)

geno2pheno®

phenotype prediction from genotype (version 3.4)

IV. Interpretation

Patient:	Birth date:	Sampling date:
Current therapy:		Viral load:

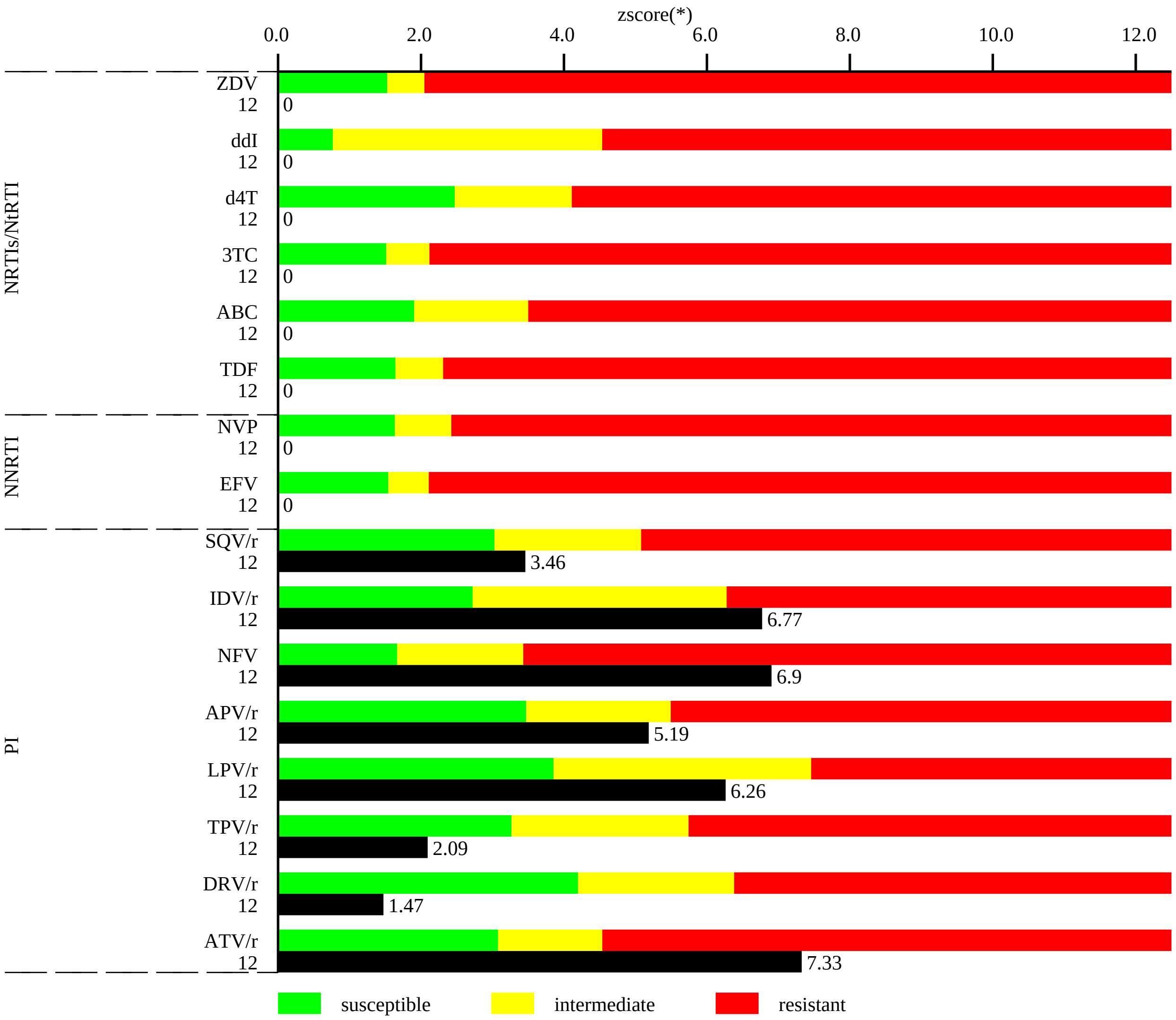

(*)number of standard deviations above mean of drug naive patients. Negative z-scores may indicate hypersusceptibility.

NRTIs/NtRTI:

NNRTIs:

PIs:

Previous genotypes:

date

signature
