# Supplementary material for: A Computational Approach for the Prediction of HIV Resistance Based on Amino Acid and Nucleotide Descriptors
Source: Molecules. 2018 Oct 24;23(11):2751. doi: 10.3390/molecules23112751 (PMC6278491; doi:10.3390/molecules23112751)
Supplement: Supplementary file 1 [file molecules-23-02751-s001.zip › Geno2Pheno-test-set-Predictions-PDF/geno2pheno-results/13.pdf]

# geno2pheno®

phenotype prediction from genotype (version 3.4)

## I. General information

| | | | |
|---|---|---|---|
| Patient: | | Study Id: | |
| Birth date: | | Viral load: | |
| Sample received: | | Sample collected: | |
| Sample ID: | 13 | Predicted subtype: | B (100%) |
| Sample type: | | Report date: | October 6, 2018 |
| Physician: | | Reported by: | |

## II. Substitutions

(relative to the reference strain HXB2)

| | |
|---|---|
| Protease: | V3I, L10I, K20R, L33F, E34T, E35D, M36I, S37D, K43T, M46I, G48V, I50V, I54S, I62V, L63P, A71I/V, I72V, V77I, V82A, I85V, I93L |
| Reverse transcriptase: | |

## III. Phenotype prediction

| Drug | Resistance Factor RF (*) | z-score | Scored Positions (**) |
|---|---|---|---|
| ZDV | 0 | 0 | |
| ddI | 0 | 0 | |
| d4T | 0 | 0 | |
| 3TC | 0 | 0 | |
| ABC | 0 | 0 | |
| TDF | 0 | 0 | |
| NVP | 0 | 0 | |
| EFV | 0 | 0 | |
| ETR (***) | Susceptible | | |
| RPV (***) | Susceptible | | |
| SQV | 177.825 | 14.919 | 48V 73G 90L 84I 11V 74T 88N 53F 95C 26T 1P 80T 47I 97L 24L |
| IDV | 649.319 | 16.983 | 88N 29D 1P 48V 73G 21E 65E 84I 46I 11V 10I 30D 54S 90L 78G |
| NFV | 136.899 | 11.019 | 88N 30D 97L 73G 68G 90L 46I 31T 75V 74T 84I 5L 2Q 54S 1P |
| APV | 227.363 | 16.819 | 33F 76L 50V 84I 32V 46I 37D 48V 22A 43T 63P 1P 47I 13I 97L |
| LPV | 409.877 | 17.541 | 84I 50V 46I 33F 48V 76L 22A 7Q 63P 24L 25D 82A 37D 47I 92Q |
| TPV | 1.677 | 1.096 | 69H 82A 46I 84I 47I 15I 91T 71V 90L 74T 88N 58Q 19L 13I 37D |
| DRV | 20.461 | 7.925 | 47I 33F 84I 76L 74T 73G 37D 65E 43T 93L 82A 85V 15I 7Q 62V |
| ATV | 222.779 | 16.087 | 73G 84I 82A 4T 33F 48V 88N 90L 7Q 24L 76L 45K 32V 93L 46I |

(*) based on LIBSVM, Copyright (c) 2000, Chih-Chung Chang and Chih-Jen Lin

(**) Positions are ordered according to their impact on the phenotype prediction. Differences with respect to HXB2 are underlined. Positions shown in red and in green contribute to an increase or decrease in resistance, repectively. At most 15 positions are shown for each drug.

(***) Resistance predictions and scored mutations for ETR and RPV were performed with rules-based drug-resistance interpretation models by HIV-GRADE (http://www.hiv-grade.de)

# geno2pheno®

phenotype prediction from genotype (version 3.4)

## IV. Interpretation

| Patient: | Birth date: | Sampling date: |
|---|---|---|
| Current therapy: | | Viral load: |

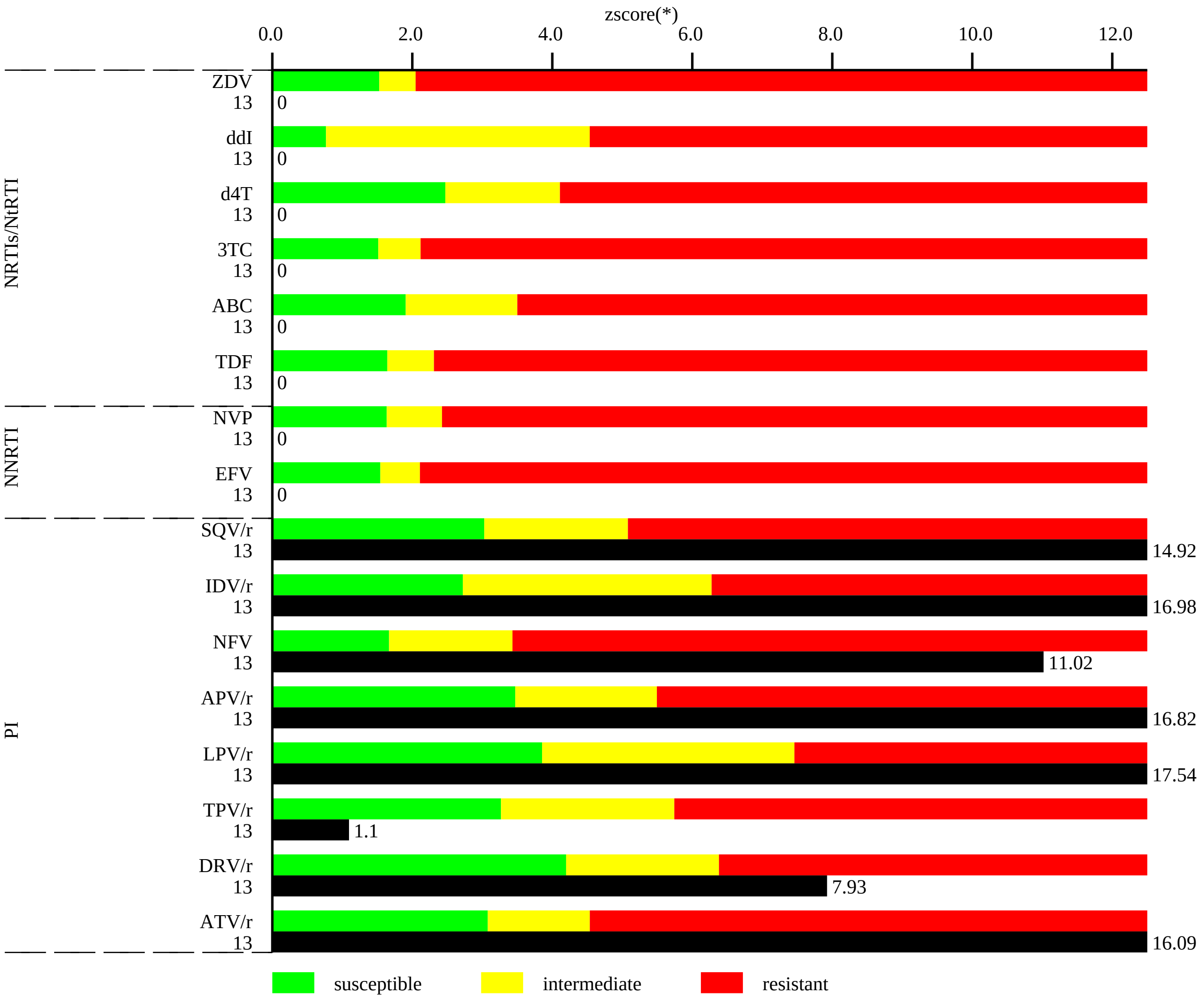


(*)number of standard deviations above mean of drug naive patients. Negative z-scores may indicate hypersusceptibility.

NRTIs/NtRTI:

NNRTIs:

PIs:

Previous genotypes:

date

signature
