# Supplementary material for: A Computational Approach for the Prediction of HIV Resistance Based on Amino Acid and Nucleotide Descriptors
Source: Molecules. 2018 Oct 24;23(11):2751. doi: 10.3390/molecules23112751 (PMC6278491; doi:10.3390/molecules23112751)
Supplement: Supplementary file 1 [file molecules-23-02751-s001.zip › Geno2Pheno-test-set-Predictions-PDF/geno2pheno-results/14.pdf]

geno2pheno®

phenotype prediction from genotype (version 3.4)

I. General information

Patient:	Study Id:
Birth date:	Viral load:
Sample received:	Sample collected:
Sample ID: 14	Predicted subtype: B (100%)
Sample type:	Report date: October 6, 2018
Physician:	Reported by:

II. Substitutions
(relative to the reference strain HXB2)

Protease:	V3I, L10I, V11I, I13I/V, K14R, K20V, S37N, L63P, A71I, G73S, P79A, I84V, L89V, L90M, I93L
Reverse transcriptase:	

III. Phenotype prediction

Drug	Resistance Factor RF (*)	z-score	Scored Positions (**)
ZDV	0	0	
ddI	0	0	
d4T	0	0	
3TC	0	0	
ABC	0	0	
TDF	0	0	
NVP	0	0	
EFV	0	0	
ETR (***)	Susceptible		
RPV (***)	Susceptible		
SQV	33.397	10.056	48G 84V 54I 74T 88N 90M 53F 95C 26T 11I 1P 80T 34E 47I 97L
IDV	16.233	7.004	54I 82V 88N 46M 90M 29D 1P 84V 21E 65E 20V 10I 85I 30D 73S
NFV	34.901	7.8	88N 54I 30D 90M 46M 84V 82V 97L 68G 36M 31T 75V 74T 5L 2Q
APV	12.686	7.686	54I 76L 50I 84V 46M 32V 89V 85I 22A 63P 1P 47I 82V 90M 97L
LPV	10.553	6.948	54I 82V 46M 50I 84V 76L 89V 22A 7Q 63P 24L 25D 47I 92Q 2Q
TPV	5.287	3.586	69H 48G 84V 33L 90M 54I 47I 14R 72I 15I 91T 73S 82V 74T 46M
DRV	5.695	4.483	47I 84V 54I 33L 76L 74T 43K 46M 36M 65E 14R 93L 48G 15I 7Q
ATV	20.118	8.648	54I 48G 84V 82V 90M 4T 88N 7Q 73S 46M 36M 24L 76L 45K 32V

(*) based on LIBSVM, Copyright (c) 2000, Chih-Chung Chang and Chih-Jen Lin

(**) Positions are ordered according to their impact on the phenotype prediction. Differences with respect to HXB2 are underlined. Positions shown in red and in green contribute to an increase or decrease in resistance, repectively. At most 15 positions are shown for each drug.

(***) Resistance predictions and scored mutations for ETR and RPV were performed with rules-based drug-resistance interpretation models by HIV-GRADE (http://www.hiv-grade.de)

geno2pheno®

phenotype prediction from genotype (version 3.4)

IV. Interpretation

Patient:	Birth date:	Sampling date:
Current therapy:		Viral load:

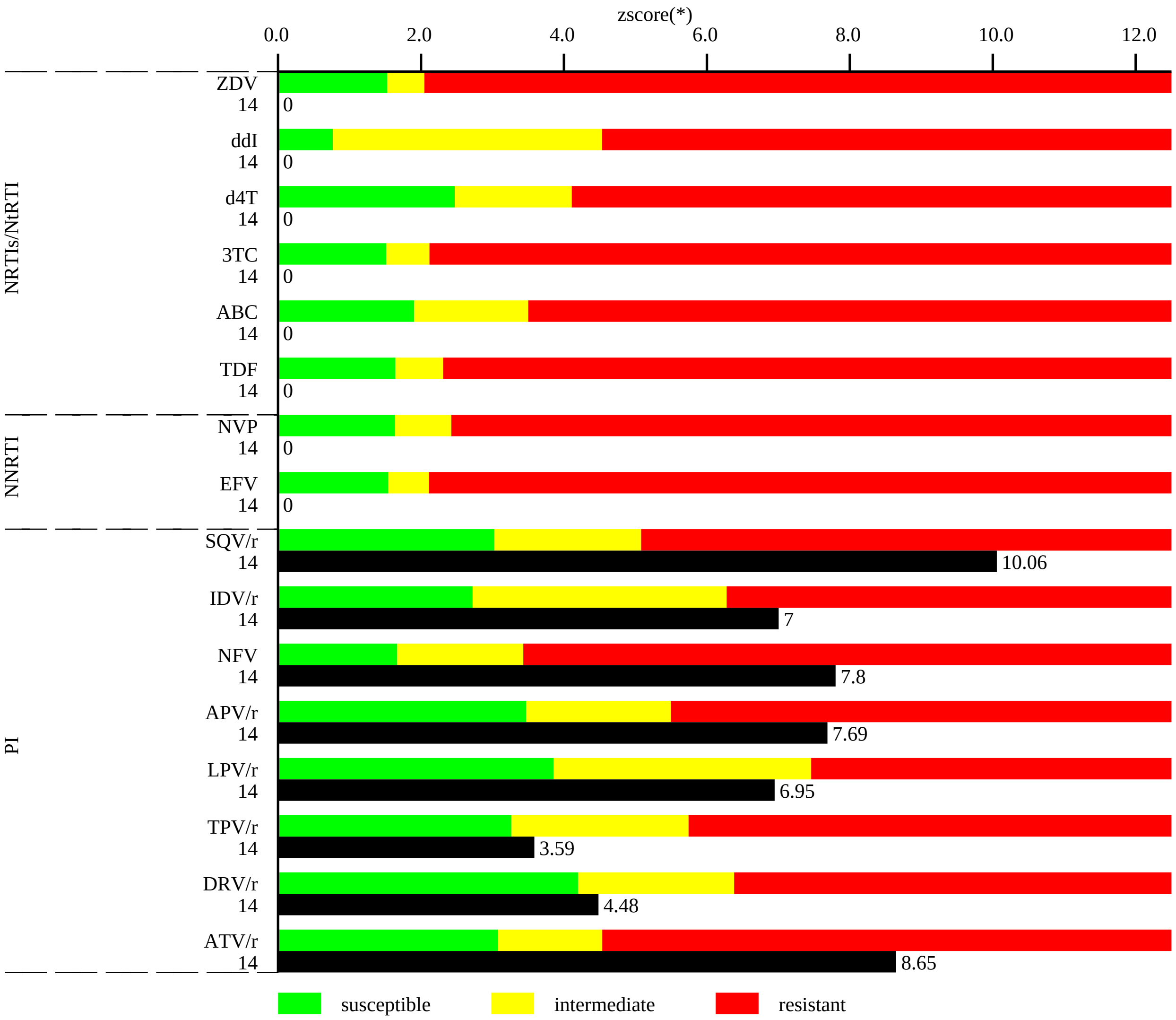

(*)number of standard deviations above mean of drug naive patients. Negative z-scores may indicate hypersusceptibility.

NRTIs/NtRTI:

NNRTIs:

PIs:

Previous genotypes:

date

signature
