# Supplementary material for: A Computational Approach for the Prediction of HIV Resistance Based on Amino Acid and Nucleotide Descriptors
Source: Molecules. 2018 Oct 24;23(11):2751. doi: 10.3390/molecules23112751 (PMC6278491; doi:10.3390/molecules23112751)
Supplement: Supplementary file 1 [file molecules-23-02751-s001.zip › Geno2Pheno-test-set-Predictions-PDF/geno2pheno-results/15.pdf]

geno2pheno®

phenotype prediction from genotype (version 3.4)

I. General information

Patient:		Study Id:	
Birth date:		Viral load:	
Sample received:		Sample collected:	
Sample ID:	15	Predicted subtype:	B (100%)
Sample type:		Report date:	October 6, 2018
Physician:		Reported by:	

II. Substitutions
(relative to the reference strain HXB2)

Protease:	V3I, L10I, L19I, K20R, E34D, E35D, M36I, S37N, R41K, M46I, I47V, I54M, L63P, A71T, I84V, I85V, L90M, I93L
Reverse transcriptase:	

III. Phenotype prediction

Drug	Resistance Factor RF (*)	z-score	Scored Positions (**)
ZDV	0	0	
ddI	0	0	
d4T	0	0	
3TC	0	0	
ABC	0	0	
TDF	0	0	
NVP	0	0	
EFV	0	0	
ETR (***)	Susceptible		
RPV (***)	Susceptible		
SQV	17.445	8.168	48G 84V 73G 11V 74T 88N 90M 47V 53F 95C 26T 1P 80T 97L 24L
IDV	121.323	12.445	82V 88N 90M 29D 1P 84V 71T 73G 21E 65E 46I 11V 10I 30D 78G
NFV	55.698	8.901	88N 30D 90M 84V 82V 97L 73G 68G 46I 31T 75V 74T 5L 2Q 1P
APV	125.769	14.945	76L 50I 84V 54M 32V 46I 47V 22A 63P 1P 13I 82V 90M 97L 45K
LPV	89.531	13.137	82V 50I 46I 84V 76L 22A 7Q 63P 24L 47V 25D 54M 92Q 2Q 48G
TPV	7.992	4.482	69H 46I 48G 84V 33L 90M 47V 72I 15I 91T 82V 74T 88N 58Q 13I
DRV	19.28	7.765	47V 84V 33L 76L 74T 43K 73G 54M 65E 93L 48G 85V 15I 7Q 8R
ATV	62.225	12.141	48G 73G 84V 82V 90M 71T 4T 88N 7Q 24L 76L 54M 45K 32V 41K

(*) based on LIBSVM, Copyright (c) 2000, Chih-Chung Chang and Chih-Jen Lin

(**) Positions are ordered according to their impact on the phenotype prediction. Differences with respect to HXB2 are underlined. Positions shown in red and in green contribute to an increase or decrease in resistance, repectively. At most 15 positions are shown for each drug.

(***) Resistance predictions and scored mutations for ETR and RPV were performed with rules-based drug-resistance interpretation models by HIV-GRADE (http://www.hiv-grade.de)

geno2pheno®

phenotype prediction from genotype (version 3.4)

IV. Interpretation

Patient:	Birth date:	Sampling date:
Current therapy:		Viral load:

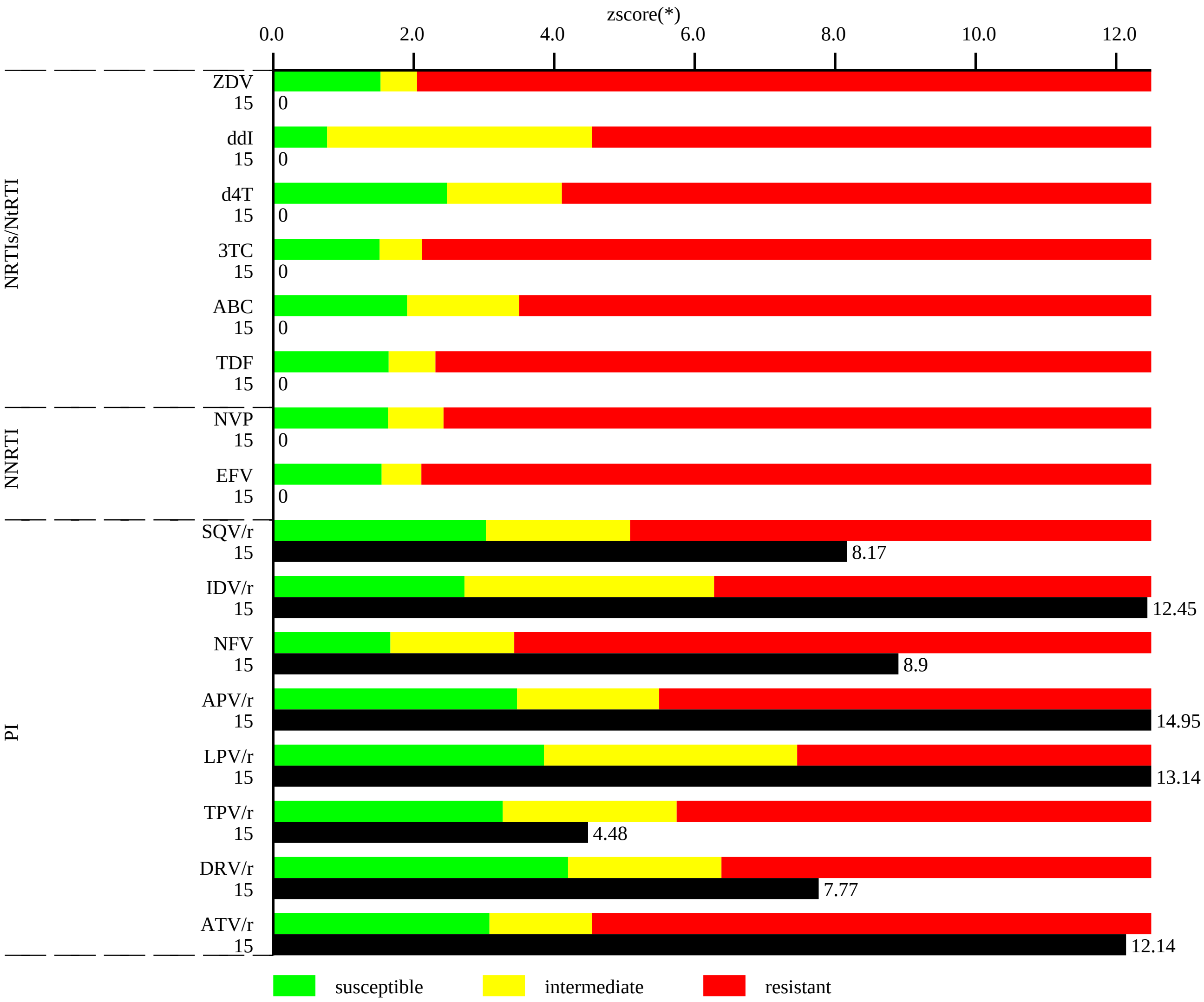

(*)number of standard deviations above mean of drug naive patients. Negative z-scores may indicate hypersusceptibility.

NRTIs/NtRTI:

NNRTIs:

PIs:

Previous genotypes:

date

signature
