# Supplementary material for: A Computational Approach for the Prediction of HIV Resistance Based on Amino Acid and Nucleotide Descriptors
Source: Molecules. 2018 Oct 24;23(11):2751. doi: 10.3390/molecules23112751 (PMC6278491; doi:10.3390/molecules23112751)
Supplement: Supplementary file 1 [file molecules-23-02751-s001.zip › Geno2Pheno-test-set-Predictions-PDF/geno2pheno-results/16.pdf]

geno2pheno®

phenotype prediction from genotype (version 3.4)

I. General information

Patient:	Study Id:
Birth date:	Viral load:
Sample received:	Sample collected:
Sample ID: 16	Predicted subtype: B (100%)
Sample type:	Report date: October 6, 2018
Physician:	Reported by:

II. Substitutions
(relative to the reference strain HXB2)

Protease:	V3I, L10I, K20R, E35D, M36I, S37N, K43T, I47V, G48M, F53L, I54V, I62V, L63P, I66F, A71V, V82T, I84V, Q92R
Reverse transcriptase:	

III. Phenotype prediction

Drug	Resistance Factor RF (*)	z-score	Scored Positions (**)
ZDV	0	0	
ddI	0	0	
d4T	0	0	
3TC	0	0	
ABC	0	0	
TDF	0	0	
NVP	0	0	
EFV	0	0	
ETR (***)	Susceptible		
RPV (***)	Susceptible		
SQV	184.139	15.021	84V 73G 90L 11V 74T 88N 47V 95C 53L 26T 1P 80T 34E 97L 24L
IDV	210.29	13.933	88N 46M 29D 1P 84V 73G 21E 65E 92R 11V 10I 85I 30D 90L 78G
NFV	153.731	11.292	88N 48M 30D 46M 84V 97L 73G 68G 90L 31T 75V 74T 92R 5L 2Q
APV	45.548	11.731	76L 50I 84V 48M 46M 32V 85I 82T 47V 22A 43T 63P 1P 13I 97L
LPV	121.938	14.031	46M 50I 54V 84V 76L 22A 7Q 63P 24L 47V 25D 48M 90L 2Q 74T
TPV	9.925	4.951	69H 84V 33L 82T 47V 72I 15I 91T 71V 90L 74T 46M 88N 58Q 19L
DRV	39.96	9.727	47V 84V 33L 76L 74T 73G 46M 65E 43T 93I 48M 15I 7Q 62V 8R
ATV	145.211	14.763	73G 84V 48M 4T 88N 90L 7Q 46M 24L 76L 45K 32V 71V 41R 2Q

(*) based on LIBSVM, Copyright (c) 2000, Chih-Chung Chang and Chih-Jen Lin

(**) Positions are ordered according to their impact on the phenotype prediction. Differences with respect to HXB2 are underlined. Positions shown in red and in green contribute to an increase or decrease in resistance, repectively. At most 15 positions are shown for each drug.

(***) Resistance predictions and scored mutations for ETR and RPV were performed with rules-based drug-resistance interpretation models by HIV-GRADE (http://www.hiv-grade.de)

geno2pheno®

phenotype prediction from genotype (version 3.4)

IV. Interpretation

Patient:	Birth date:	Sampling date:
Current therapy:		Viral load:

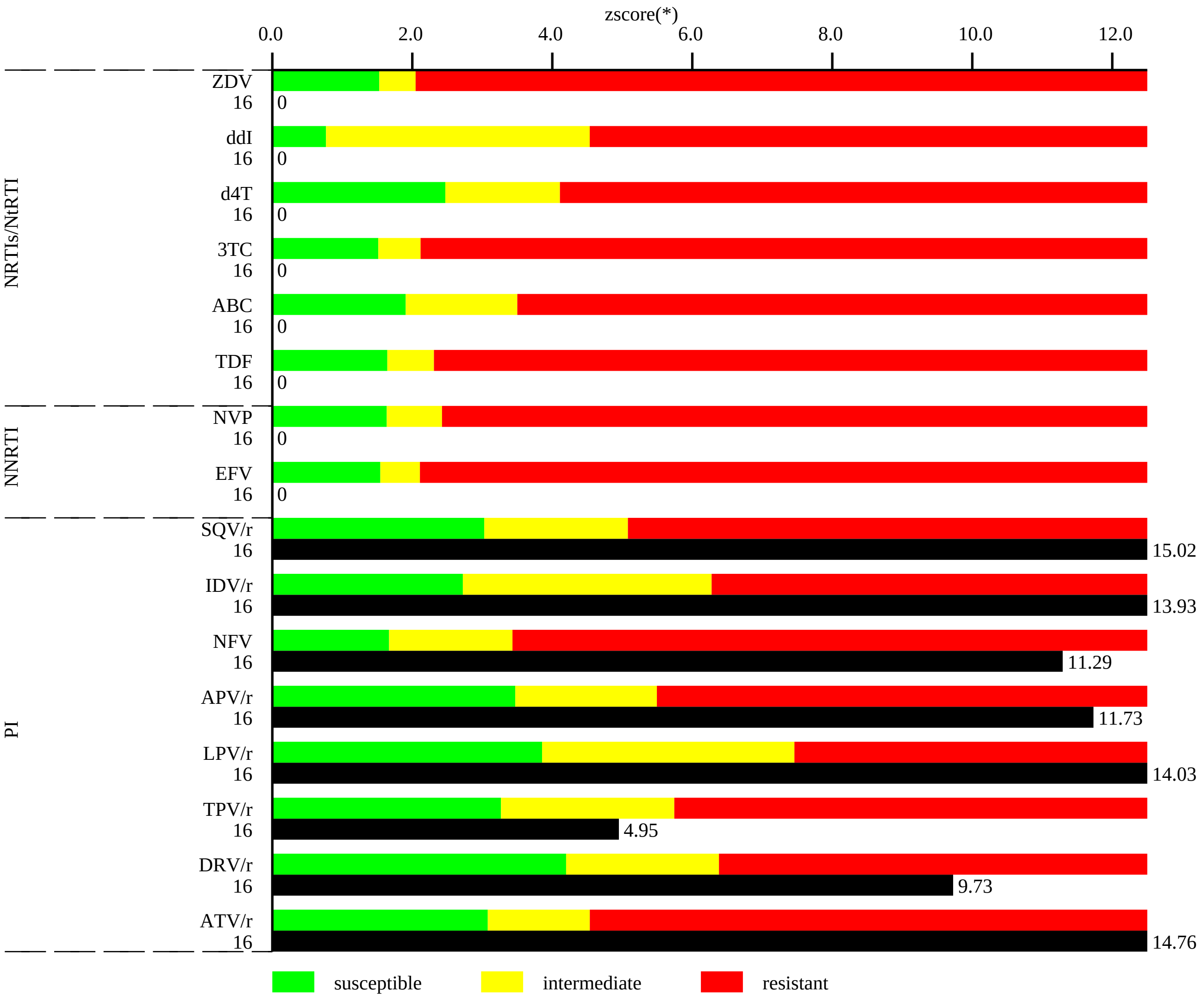

(*)number of standard deviations above mean of drug naive patients. Negative z-scores may indicate hypersusceptibility.

NRTIs/NtRTI:

NNRTIs:

PIs:

Previous genotypes:

date

signature
