# Supplementary material for: A Computational Approach for the Prediction of HIV Resistance Based on Amino Acid and Nucleotide Descriptors
Source: Molecules. 2018 Oct 24;23(11):2751. doi: 10.3390/molecules23112751 (PMC6278491; doi:10.3390/molecules23112751)
Supplement: Supplementary file 1 [file molecules-23-02751-s001.zip › Geno2Pheno-test-set-Predictions-PDF/geno2pheno-results/17.pdf]

geno2pheno®

phenotype prediction from genotype (version 3.4)

I. General information

Patient:	Study Id:
Birth date:	Viral load:
Sample received:	Sample collected:
Sample ID: 17	Predicted subtype: B (100%)
Sample type:	Report date: October 6, 2018
Physician:	Reported by:

II. Substitutions
(relative to the reference strain HXB2)

Protease:	V3I, L10I, I15V, L19I, K20R, E35D, M36I, S37E, R41K, I54V, Q58E, D60N, I62V, L63P, K70R, A71V, G73S, V82A, L90M, I93L
Reverse transcriptase:	

III. Phenotype prediction

Drug	Resistance Factor RF (*)	z-score	Scored Positions (**)
ZDV	0	0	
ddI	0	0	
d4T	0	0	
3TC	0	0	
ABC	0	0	
TDF	0	0	
NVP	0	0	
EFV	0	0	
ETR (***)	Susceptible		
RPV (***)	Susceptible		
SQV	55.098	11.512	48G 84I 11V 74T 88N 90M 53F 95C 26T 1P 80T 34E 47I 97L 73S
IDV	60.656	10.57	88N 46M 90M 29D 1P 21E 65E 84I 11V 10I 85I 30D 73S 78G 98N
NFV	58.247	9.006	88N 30D 90M 46M 97L 68G 31T 75V 74T 84I 5L 2Q 1P 29D 48G
APV	12.24	7.573	76L 50I 84I 46M 32V 85I 22A 63P 1P 47I 13I 90M 97L 45K 10I
LPV	37.046	10.582	46M 84I 50I 54V 76L 22A 7Q 63P 24L 25D 82A 47I 92Q 2Q 48G
TPV	5.604	3.712	69H 82A 48G 84I 33L 90M 47I 72I 91T 71V 15V 73S 74T 46M 88N
DRV	2.577	2.35	47I 84I 33L 76L 74T 43K 46M 65E 93L 48G 82A 7Q 62V 8R 16G
ATV	85.516	13.125	48G 84I 90M 82A 4T 88N 7Q 73S 46M 24L 76L 45K 32V 41K 93L

(*) based on LIBSVM, Copyright (c) 2000, Chih-Chung Chang and Chih-Jen Lin

(**) Positions are ordered according to their impact on the phenotype prediction. Differences with respect to HXB2 are underlined. Positions shown in red and in green contribute to an increase or decrease in resistance, repectively. At most 15 positions are shown for each drug.

(***) Resistance predictions and scored mutations for ETR and RPV were performed with rules-based drug-resistance interpretation models by HIV-GRADE (http://www.hiv-grade.de)

geno2pheno®

phenotype prediction from genotype (version 3.4)

IV. Interpretation

Patient:	Birth date:	Sampling date:
Current therapy:		Viral load:

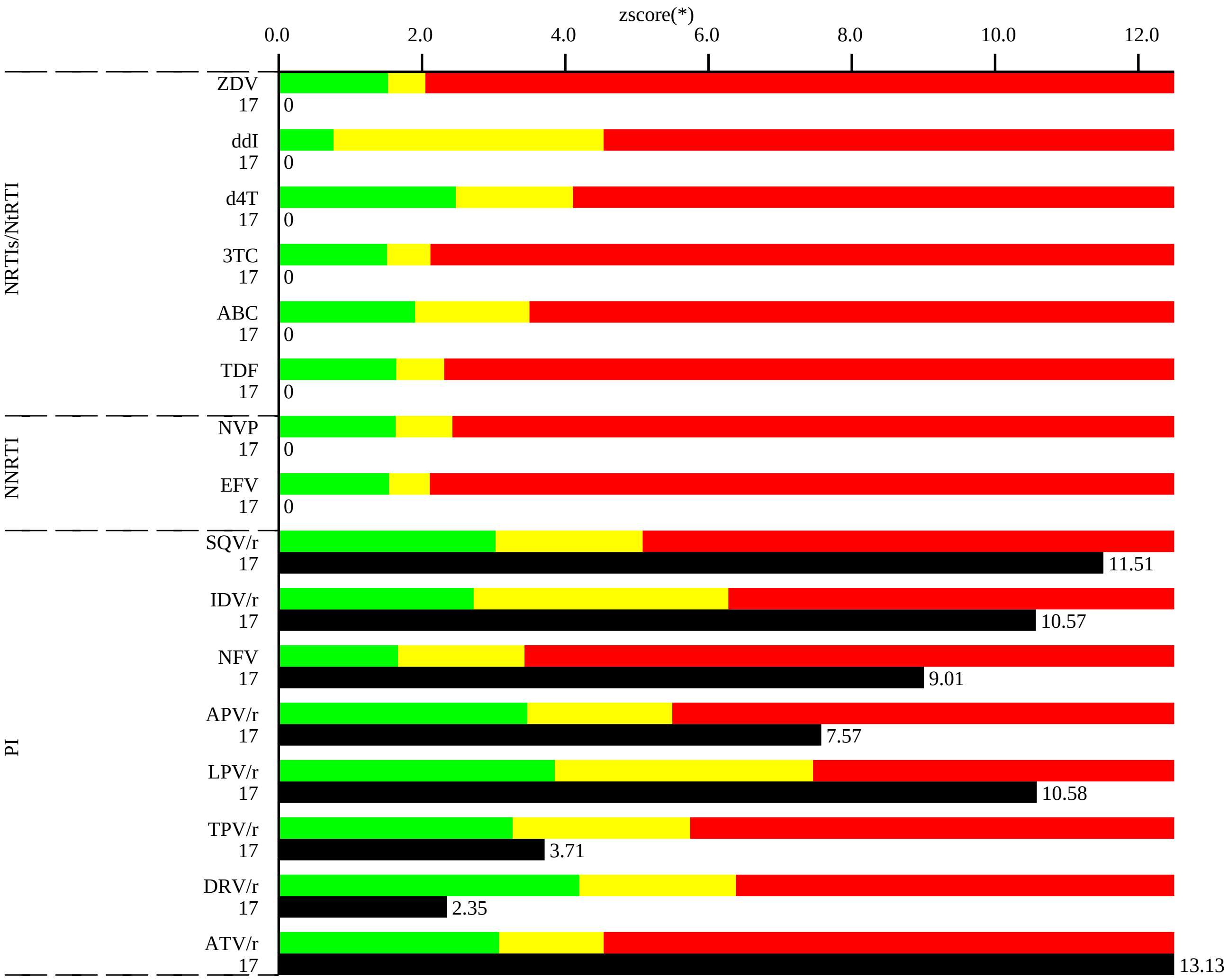

susceptible intermediate resistant

(*)number of standard deviations above mean of drug naive patients. Negative z-scores may indicate hypersusceptibility.

NRTIs/NtRTI:

NNRTIs:

PIs:

Previous genotypes:

date

signature
