# Supplementary material for: A Computational Approach for the Prediction of HIV Resistance Based on Amino Acid and Nucleotide Descriptors
Source: Molecules. 2018 Oct 24;23(11):2751. doi: 10.3390/molecules23112751 (PMC6278491; doi:10.3390/molecules23112751)
Supplement: Supplementary file 1 [file molecules-23-02751-s001.zip › Geno2Pheno-test-set-Predictions-PDF/geno2pheno-results/18.pdf]

geno2pheno®

phenotype prediction from genotype (version 3.4)

I. General information

Patient:		Study Id:	
Birth date:		Viral load:	
Sample received:		Sample collected:	
Sample ID:	18	Predicted subtype:	B (100%)
Sample type:		Report date:	October 6, 2018
Physician:		Reported by:	

II. Substitutions

(relative to the reference strain HXB2)

Protease:	V3I, L10I, I13V, K20I, L33V, E35D, M36I, S37T, M46I, I54V, Q58E/Q, I62V, L63P, I64I/V, A71V, T74P, V82L, L90M, I93L
Reverse transcriptase:	

III. Phenotype prediction

Drug	Resistance Factor RF (*)	z-score	Scored Positions (**)
ZDV	0	0	
ddI	0	0	
d4T	0	0	
3TC	0	0	
ABC	0	0	
TDF	0	0	
NVP	0	0	
EFV	0	0	
ETR (***)	Susceptible		
RPV (***)	Susceptible		
SQV	80.027	12.597	48G 73G 84I 11V 88N 90M 53F 95C 26T 1P 80T 34E 47I 97L 24L
IDV	171.058	13.374	88N 90M 29D 1P 73G 21E 65E 84I 46I 11V 10I 85I 30D 78G 98N
NFV	389.547	13.481	88N 30D 90M 97L 73G 68G 46I 31T 75V 84I 5L 2Q 1P 29D 48G
APV	23.245	9.603	76L 50I 84I 32V 46I 85I 22A 63P 1P 47I 90M 97L 45K 10I 58E
LPV	68.867	12.377	84I 50I 46I 54V 76L 22A 33V 7Q 63P 24L 25D 47I 92Q 2Q 48G
TPV	10.653	5.105	69H 46I 48G 84I 90M 47I 72I 15I 91T 71V 88N 19L 39P 89L 41R
DRV	7.88	5.357	47I 84I 76L 43K 73G 65E 93L 48G 15I 7Q 62V 8R 16G 2Q 32V
ATV	86.244	13.151	48G 73G 84I 90M 4T 88N 7Q 24L 76L 45K 32V 93L 46I 71V 53F

(*) based on LIBSVM, Copyright (c) 2000, Chih-Chung Chang and Chih-Jen Lin

(**) Positions are ordered according to their impact on the phenotype prediction. Differences with respect to HXB2 are underlined. Positions shown in red and in green contribute to an increase or decrease in resistance, repectively. At most 15 positions are shown for each drug.

(***) Resistance predictions and scored mutations for ETR and RPV were performed with rules-based drug-resistance interpretation models by HIV-GRADE (http://www.hiv-grade.de)

geno2pheno®

phenotype prediction from genotype (version 3.4)

IV. Interpretation

Patient:	Birth date:	Sampling date:
Current therapy:		Viral load:

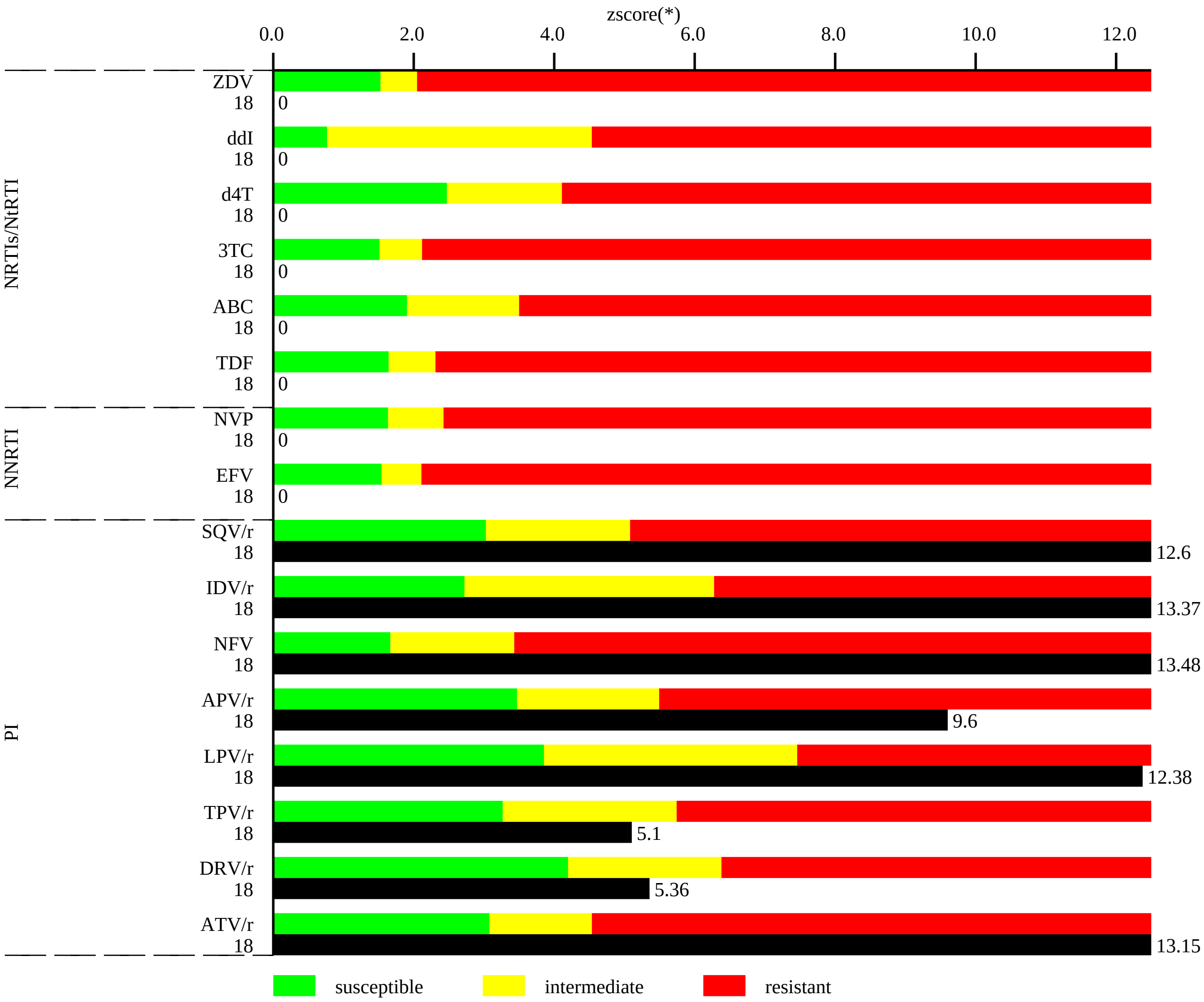

(*)number of standard deviations above mean of drug naive patients. Negative z-scores may indicate hypersusceptibility.

NRTIs/NtRTI:

NNRTIs:

PIs:

Previous genotypes:

date

signature
