# Supplementary material for: A Computational Approach for the Prediction of HIV Resistance Based on Amino Acid and Nucleotide Descriptors
Source: Molecules. 2018 Oct 24;23(11):2751. doi: 10.3390/molecules23112751 (PMC6278491; doi:10.3390/molecules23112751)
Supplement: Supplementary file 1 [file molecules-23-02751-s001.zip › Geno2Pheno-test-set-Predictions-PDF/geno2pheno-results/19.pdf]

geno2pheno®

phenotype prediction from genotype (version 3.4)

I. General information

Patient:		Study Id:	
Birth date:		Viral load:	
Sample received:		Sample collected:	
Sample ID:	19	Predicted subtype:	B (100%)
Sample type:		Report date:	October 6, 2018
Physician:		Reported by:	

II. Substitutions
(relative to the reference strain HXB2)

Protease:	V3I, L10I/T, I13V, V32I, L33F, M36I, S37N, R41K/R, M46I/L/M, I47A, L63P, K70E, A71V, V82A, L90M, I93L
Reverse transcriptase:	

III. Phenotype prediction

Drug	Resistance Factor RF (*)	z-score	Scored Positions (**)
ZDV	0	0	
ddI	0	0	
d4T	0	0	
3TC	0	0	
ABC	0	0	
TDF	0	0	
NVP	0	0	
EFV	0	0	
ETR (***)	Susceptible		
RPV (***)	Susceptible		
SQV	8.326	6.017	48G 73G 84I 54I 11V 74T 88N 90M 53F 95C 26T 1P 80T 34E 97L
IDV	38.694	9.354	54I 88N 90M 29D 1P 73G 21E 65E 84I 32I 46I 11V 10I 85I 30D
NFV	31.358	7.548	88N 54I 30D 90M 97L 20K 73G 68G 46I 31T 75V 74T 84I 5L 2Q
APV	49.489	11.994	54I 33F 76L 50I 84I 32I 46I 85I 22A 63P 1P 90M 97L 45K 10I
LPV	26.31	9.592	54I 84I 50I 46I 33F 76L 22A 7Q 63P 24L 20K 25D 82A 92Q 2Q
TPV	4.496	3.234	69H 82A 48G 84I 90M 54I 72I 15I 91T 71V 20K 74T 46M 88N 58Q
DRV	6.63	4.893	33F 84I 54I 76L 74T 43K 73G 65E 93L 48G 82A 32I 15I 7Q 8R
ATV	43.579	11.039	54I 48G 73G 84I 90M 82A 4T 33F 46L 88N 7Q 20K 24L 76L 45K

(*) based on LIBSVM, Copyright (c) 2000, Chih-Chung Chang and Chih-Jen Lin

(**) Positions are ordered according to their impact on the phenotype prediction. Differences with respect to HXB2 are underlined. Positions shown in red and in green contribute to an increase or decrease in resistance, repectively. At most 15 positions are shown for each drug.

(***) Resistance predictions and scored mutations for ETR and RPV were performed with rules-based drug-resistance interpretation models by HIV-GRADE (http://www.hiv-grade.de)

geno2pheno®

phenotype prediction from genotype (version 3.4)

IV. Interpretation

Patient:	Birth date:	Sampling date:
Current therapy:		Viral load:

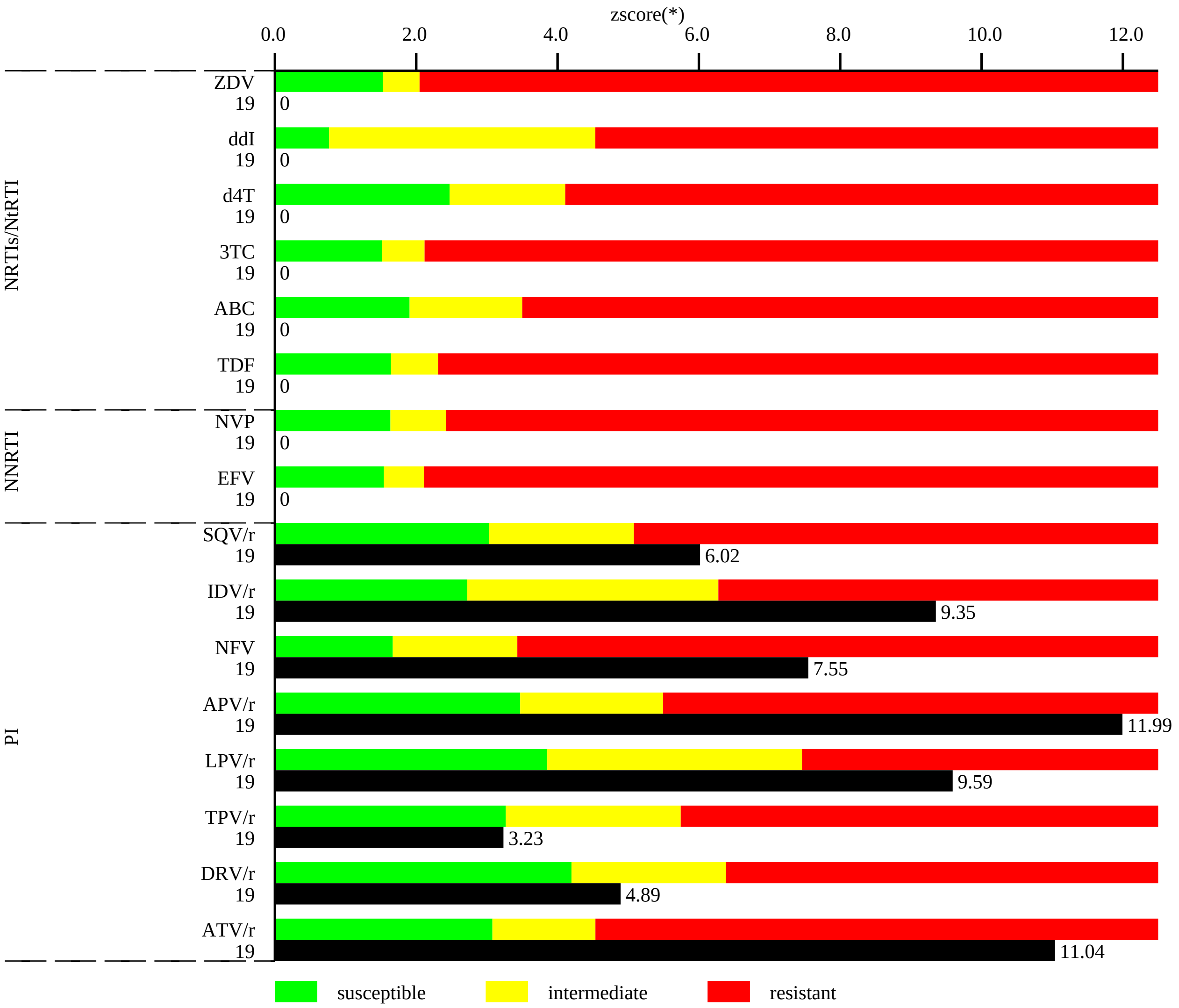

(*)number of standard deviations above mean of drug naive patients. Negative z-scores may indicate hypersusceptibility.

NRTIs/NtRTI:
NNRTIs:
PIs:
Previous genotypes:

date

signature
