# Supplementary material for: A Computational Approach for the Prediction of HIV Resistance Based on Amino Acid and Nucleotide Descriptors
Source: Molecules. 2018 Oct 24;23(11):2751. doi: 10.3390/molecules23112751 (PMC6278491; doi:10.3390/molecules23112751)
Supplement: Supplementary file 1 [file molecules-23-02751-s001.zip › Geno2Pheno-test-set-Predictions-PDF/geno2pheno-results/2.pdf]

geno2pheno®

phenotype prediction from genotype (version 3.4)

I. General information

Patient:	Study Id:
Birth date:	Viral load:
Sample received:	Sample collected:
Sample ID: 2	Predicted subtype: 07_BC (100%)
Sample type:	Report date: October 6, 2018
Physician:	Reported by:

II. Substitutions
(relative to the reference strain HXB2)

Protease:	L10I, I13V, G16A, K20K/M, S37N, M46I, I54V, L63P, I66F, C67Y, H69K, A71V, I72F, G73T, I84V, L90M, I93L
Reverse transcriptase:	

III. Phenotype prediction

Drug	Resistance Factor RF (*)	z-score	Scored Positions (**)
ZDV	0	0	
ddI	0	0	
d4T	0	0	
3TC	0	0	
ABC	0	0	
TDF	0	0	
NVP	0	0	
EFV	0	0	
ETR (***)	Susceptible		
RPV (***)	Susceptible		
SQV	72.095	12.294	48G 84V 11V 74T 88N 90M 53F 95C 26T 73T 80T 34E 47I 97L 24L
IDV	299.658	14.891	82V 88N 90M 29D 84V 21E 65E 46I 67Y 73T 11V 10I 85I 30D 78G
NFV	221.514	12.152	88N 30D 90M 84V 82V 97L 68G 36M 46I 31T 75V 74T 5L 29D 48G
APV	25.91	9.946	76L 50I 84V 32V 46I 85I 22A 63P 47I 82V 90M 97L 45K 10I 21E
LPV	33.129	10.259	82V 50I 46I 54V 84V 76L 22A 7Q 63P 24L 25D 47I 92Q 48G 74T
TPV	4.262	3.118	46I 48G 84V 33L 90M 47I 15I 91T 71V 69K 82V 74T 88N 58Q 19L
DRV	5.29	4.285	47I 84V 33L 76L 74T 43K 36M 65E 93L 48G 15I 7Q 8R 32V
ATV	69.993	12.505	48G 84V 82V 90M 73T 4T 88N 7Q 36M 24L 76L 45K 32V 93L 46I

(*) based on LIBSVM, Copyright (c) 2000, Chih-Chung Chang and Chih-Jen Lin

(**) Positions are ordered according to their impact on the phenotype prediction. Differences with respect to HXB2 are underlined. Positions shown in red and in green contribute to an increase or decrease in resistance, repectively. At most 15 positions are shown for each drug.

(***) Resistance predictions and scored mutations for ETR and RPV were performed with rules-based drug-resistance interpretation models by HIV-GRADE (http://www.hiv-grade.de)

geno2pheno®

phenotype prediction from genotype (version 3.4)

IV. Interpretation

Patient:	Birth date:	Sampling date:
Current therapy:		Viral load:

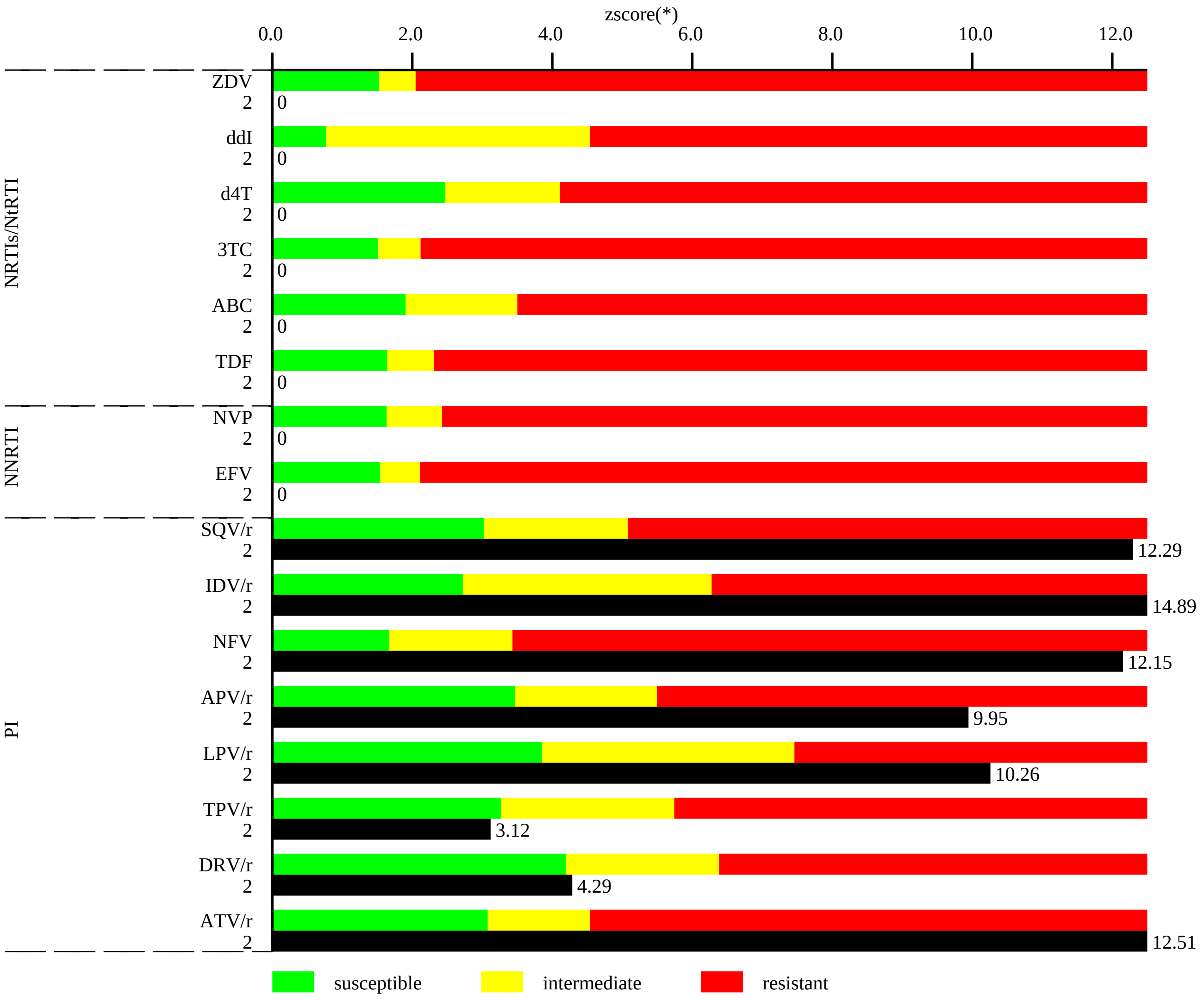

(*)number of standard deviations above mean of drug naive patients. Negative z-scores may indicate hypersusceptibility.

NRTIs/NtRTI:

NNRTIs:

PIs:

Previous genotypes:

date

signature
