# Supplementary material for: A Computational Approach for the Prediction of HIV Resistance Based on Amino Acid and Nucleotide Descriptors
Source: Molecules. 2018 Oct 24;23(11):2751. doi: 10.3390/molecules23112751 (PMC6278491; doi:10.3390/molecules23112751)
Supplement: Supplementary file 1 [file molecules-23-02751-s001.zip › Geno2Pheno-test-set-Predictions-PDF/geno2pheno-results/24.pdf]

geno2pheno®

phenotype prediction from genotype (version 3.4)

I. General information

Patient:		Study Id:	
Birth date:		Viral load:	
Sample received:		Sample collected:	
Sample ID:	24	Predicted subtype:	06_CPX (100%)
Sample type:		Report date:	October 6, 2018
Physician:		Reported by:	

II. Substitutions

(relative to the reference strain HXB2)

Protease:	V3I, L10F, V11I, T12P, I13V, I15V, L19P, K20I, E35G, M36I, S37N, M46I, I54V, L63P, A71V, I72L, G73S, I84V, L90M, I93L
Reverse transcriptase:	

III. Phenotype prediction

Drug	Resistance Factor RF (*)	z-score	Scored Positions (**)
ZDV	0	0	
ddI	0	0	
d4T	0	0	
3TC	0	0	
ABC	0	0	
TDF	0	0	
NVP	0	0	
EFV	0	0	
ETR (***)	Susceptible		
RPV (***)	Susceptible		
SQV	173.461	14.847	48G 84V 74T 88N 90M 53F 95C 26T 11I 1P 80T 35G 34E 72L 47I
IDV	199.52	13.791	82V 88N 90M 29D 1P 84V 21E 65E 46I 72L 85I 30D 73S 78G 98N
NFV	291.072	12.795	88N 30D 90M 84V 82V 97L 68G 46I 31T 75V 74T 5L 2Q 1P 29D
APV	68.235	13.01	76L 50I 84V 32V 46I 85I 10F 22A 63P 1P 47I 82V 90M 97L 45K
LPV	95.561	13.326	82V 50I 46I 54V 84V 76L 22A 10F 7Q 63P 24L 25D 47I 92Q 2Q
TPV	18.552	6.307	69H 46I 48G 84V 33L 90M 47I 91T 71V 15V 73S 82V 74T 88N 58Q
DRV	9.735	5.926	47I 84V 33L 76L 74T 43K 65E 10F 93L 48G 7Q 8R 16G 2Q 32V
ATV	239.788	16.315	48G 84V 82V 90M 4T 72L 88N 35G 7Q 73S 24L 76L 45K 19P 32V

(*) based on LIBSVM, Copyright (c) 2000, Chih-Chung Chang and Chih-Jen Lin

(**) Positions are ordered according to their impact on the phenotype prediction. Differences with respect to HXB2 are underlined. Positions shown in red and in green contribute to an increase or decrease in resistance, repectively. At most 15 positions are shown for each drug.

(***) Resistance predictions and scored mutations for ETR and RPV were performed with rules-based drug-resistance interpretation models by HIV-GRADE (http://www.hiv-grade.de)

geno2pheno®

phenotype prediction from genotype (version 3.4)

IV. Interpretation

Patient:	Birth date:	Sampling date:
Current therapy:		Viral load:

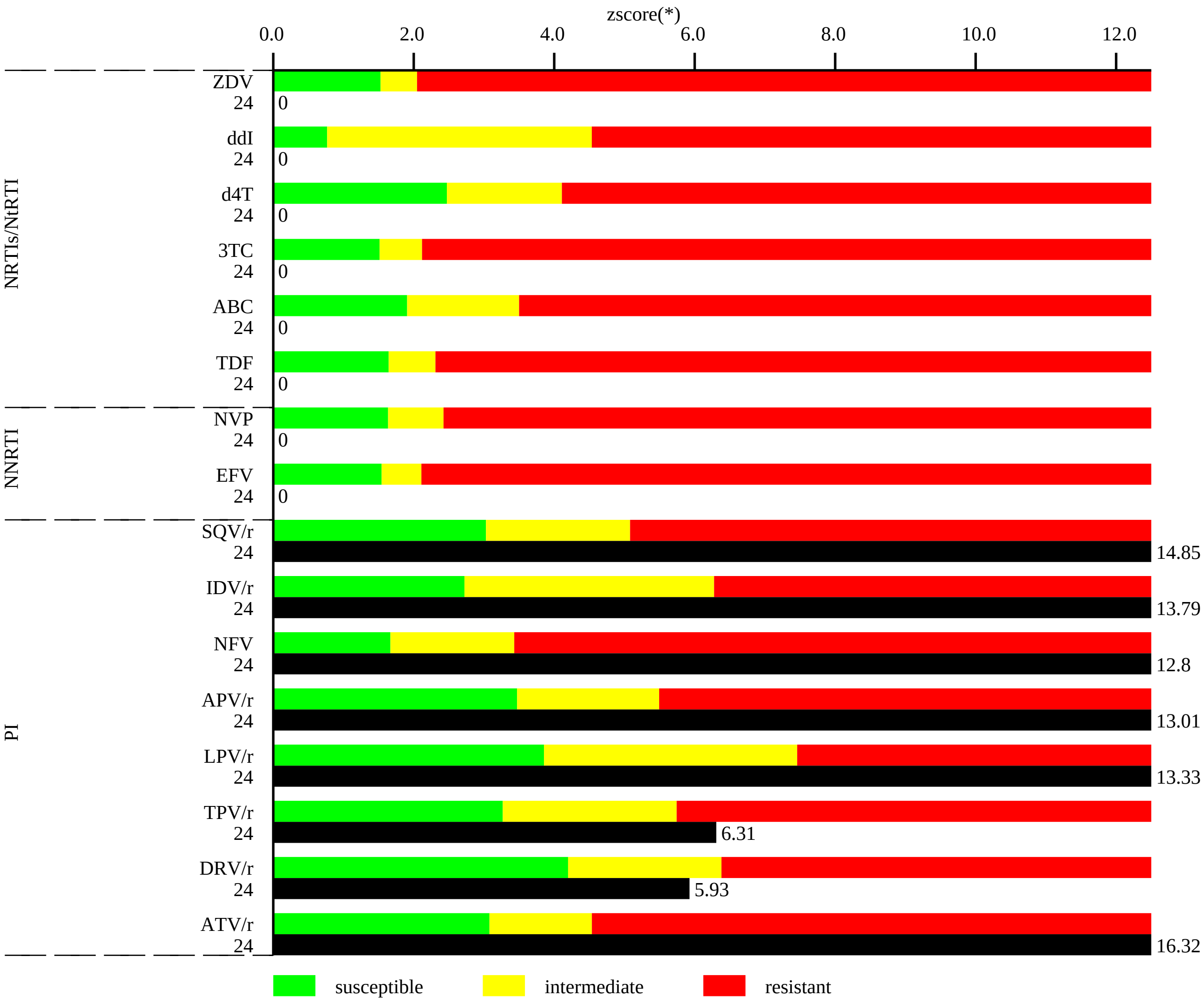

(*)number of standard deviations above mean of drug naive patients. Negative z-scores may indicate hypersusceptibility.

NRTIs/NtRTI:

NNRTIs:

PIs:

Previous genotypes:

date

signature
