# Supplementary material for: A Computational Approach for the Prediction of HIV Resistance Based on Amino Acid and Nucleotide Descriptors
Source: Molecules. 2018 Oct 24;23(11):2751. doi: 10.3390/molecules23112751 (PMC6278491; doi:10.3390/molecules23112751)
Supplement: Supplementary file 1 [file molecules-23-02751-s001.zip › Geno2Pheno-test-set-Predictions-PDF/geno2pheno-results/25.pdf]

geno2pheno®

phenotype prediction from genotype (version 3.4)

I. General information

Patient:	Study Id:
Birth date:	Viral load:
Sample received:	Sample collected:
Sample ID: 25	Predicted subtype: B (100%)
Sample type:	Report date: October 6, 2018
Physician:	Reported by:

II. Substitutions

(relative to the reference strain HXB2)

Protease:	V3I, L10F, V11I, T12P, I13V, I15V, L19P, K20T, V32I, L33F, E35G, M36I, S37N, I54I/V, I62V, L63P, K70T, A71I, G73S, P79A, I84V, L89V, L90M
Reverse transcriptase:	

III. Phenotype prediction

Drug	Resistance Factor RF (*)	z-score	Scored Positions (**)
ZDV	0	0	
ddI	0	0	
d4T	0	0	
3TC	0	0	
ABC	0	0	
TDF	0	0	
NVP	0	0	
EFV	0	0	
ETR (***)	Susceptible		
RPV (***)	Susceptible		
SQV	117.905	13.724	48G 84V 74T 88N 90M 53F 95C 26T 11I 1P 80T 35G 34E 47I 97L
IDV	68.937	10.916	82V 88N 46M 90M 29D 1P 84V 21E 65E 32I 85I 30D 73S 78G 98N
NFV	202.891	11.945	88N 30D 90M 46M 84V 82V 97L 68G 31T 75V 74T 5L 2Q 1P 29D
APV	271.857	17.385	33F 76L 50I 84V 46M 32I 89V 85I 10F 22A 63P 1P 47I 82V 90M
LPV	93.157	13.252	82V 46M 50I 54V 84V 33F 76L 89V 22A 10F 7Q 63P 24L 25D 47I
TPV	18.574	6.31	69H 48G 84V 90M 47I 72I 91T 15V 73S 82V 74T 46M 88N 58Q 39P
DRV	31.223	9.063	47I 33F 84V 76L 74T 43K 46M 65E 10F 48G 32I 93I 7Q 62V 8R
ATV	171.332	15.275	48G 84V 82V 90M 4T 33F 88N 35G 7Q 73S 46M 24L 76L 45K 19P

(*) based on LIBSVM, Copyright (c) 2000, Chih-Chung Chang and Chih-Jen Lin

(**) Positions are ordered according to their impact on the phenotype prediction. Differences with respect to HXB2 are underlined. Positions shown in red and in green contribute to an increase or decrease in resistance, repectively. At most 15 positions are shown for each drug.

(***) Resistance predictions and scored mutations for ETR and RPV were performed with rules-based drug-resistance interpretation models by HIV-GRADE (http://www.hiv-grade.de)

geno2pheno®

phenotype prediction from genotype (version 3.4)

IV. Interpretation

Patient:	Birth date:	Sampling date:
Current therapy:		Viral load:

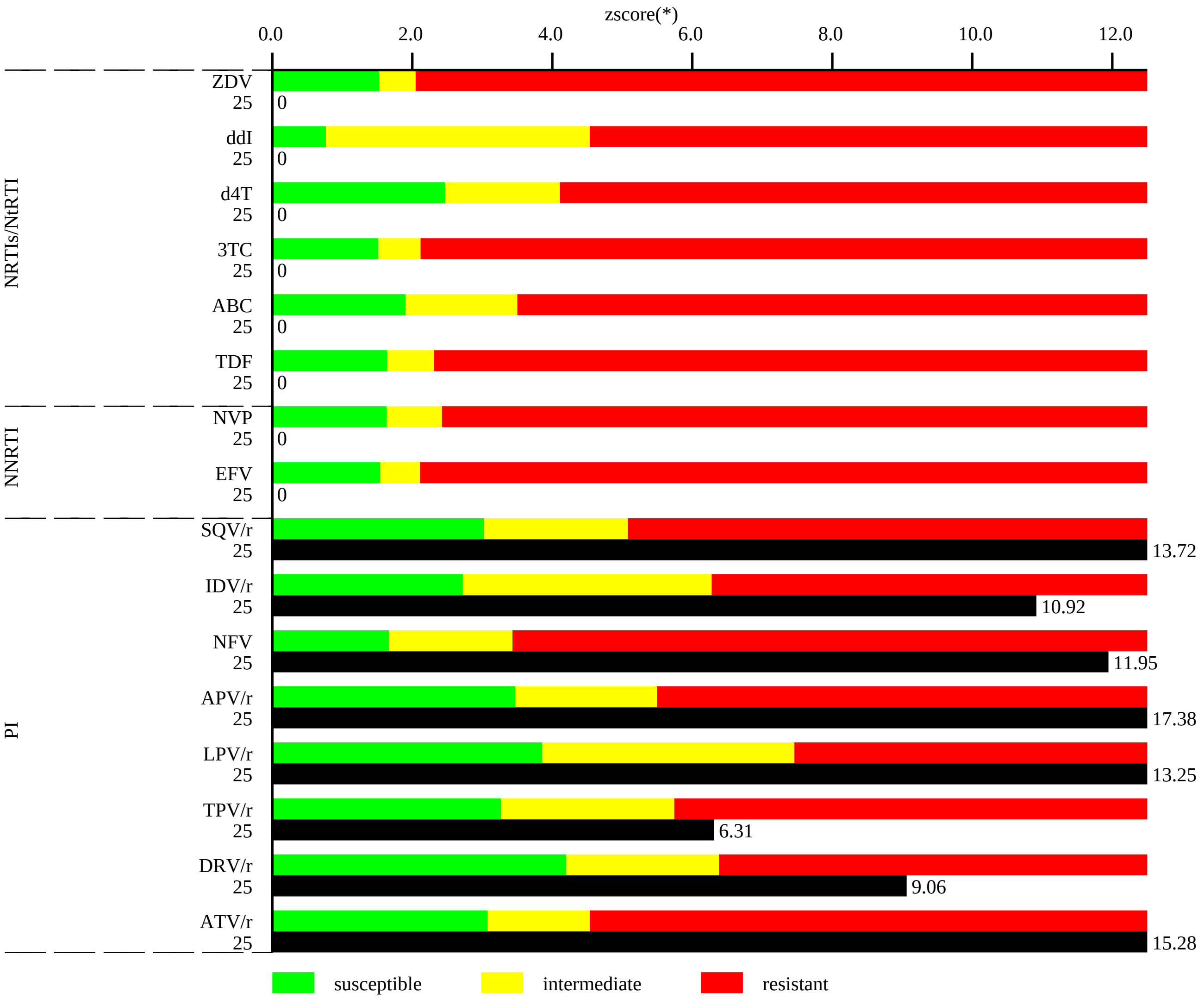

(*)number of standard deviations above mean of drug naive patients. Negative z-scores may indicate hypersusceptibility.

NRTIs/NtRTI:

NNRTIs:

PIs:

Previous genotypes:

date

signature
