# Supplementary material for: A Computational Approach for the Prediction of HIV Resistance Based on Amino Acid and Nucleotide Descriptors
Source: Molecules. 2018 Oct 24;23(11):2751. doi: 10.3390/molecules23112751 (PMC6278491; doi:10.3390/molecules23112751)
Supplement: Supplementary file 1 [file molecules-23-02751-s001.zip › Geno2Pheno-test-set-Predictions-PDF/geno2pheno-results/26.pdf]

geno2pheno®

phenotype prediction from genotype (version 3.4)

I. General information

Patient:	Study Id:
Birth date:	Viral load:
Sample received:	Sample collected:
Sample ID: 26	Predicted subtype: B (100%)
Sample type:	Report date: October 6, 2018
Physician:	Reported by:

II. Substitutions
(relative to the reference strain HXB2)

Protease:	V3I, L10F, I13M, K20I, M36I, S37D, M46I, I54V, I62V, L63P, I66I/V, C67F, A71T, I72T, G73T, I84V, L90M, I93L
Reverse transcriptase:	

III. Phenotype prediction

Drug	Resistance Factor RF (*)	z-score	Scored Positions (**)
ZDV	0	0	
ddI	0	0	
d4T	0	0	
3TC	0	0	
ABC	0	0	
TDF	0	0	
NVP	0	0	
EFV	0	0	
ETR (***)	Susceptible		
RPV (***)	Susceptible		
SQV	172.644	14.833	48G 84V 11V 74T 88N 90M 53F 95C 26T 1P 73T 80T 34E 47I 97L
IDV	195.164	13.731	82V 88N 90M 29D 1P 84V 71T 21E 65E 46I 73T 11V 85I 30D 78G
NFV	252.375	12.459	88N 30D 90M 84V 82V 97L 68G 46I 31T 75V 74T 5L 2Q 1P 29D
APV	47.805	11.884	76L 50I 84V 32V 46I 85I 37D 10F 22A 63P 1P 47I 82V 90M 97L
LPV	88.259	13.095	82V 50I 46I 54V 84V 76L 22A 10F 7Q 63P 24L 25D 37D 47I 92Q
TPV	7.955	4.471	69H 46I 48G 84V 33L 90M 47I 15I 91T 82V 74T 88N 58Q 19L 37D
DRV	18.03	7.585	47I 84V 33L 76L 74T 43K 37D 65E 10F 93L 48G 72T 15I 7Q 62V
ATV	151.579	14.896	48G 84V 82V 90M 73T 71T 4T 88N 7Q 24L 76L 45K 32V 93L 46I

(*) based on LIBSVM, Copyright (c) 2000, Chih-Chung Chang and Chih-Jen Lin

(**) Positions are ordered according to their impact on the phenotype prediction. Differences with respect to HXB2 are underlined. Positions shown in red and in green contribute to an increase or decrease in resistance, repectively. At most 15 positions are shown for each drug.

(***) Resistance predictions and scored mutations for ETR and RPV were performed with rules-based drug-resistance interpretation models by HIV-GRADE (http://www.hiv-grade.de)

geno2pheno®

phenotype prediction from genotype (version 3.4)

IV. Interpretation

Patient:	Birth date:	Sampling date:
Current therapy:		Viral load:

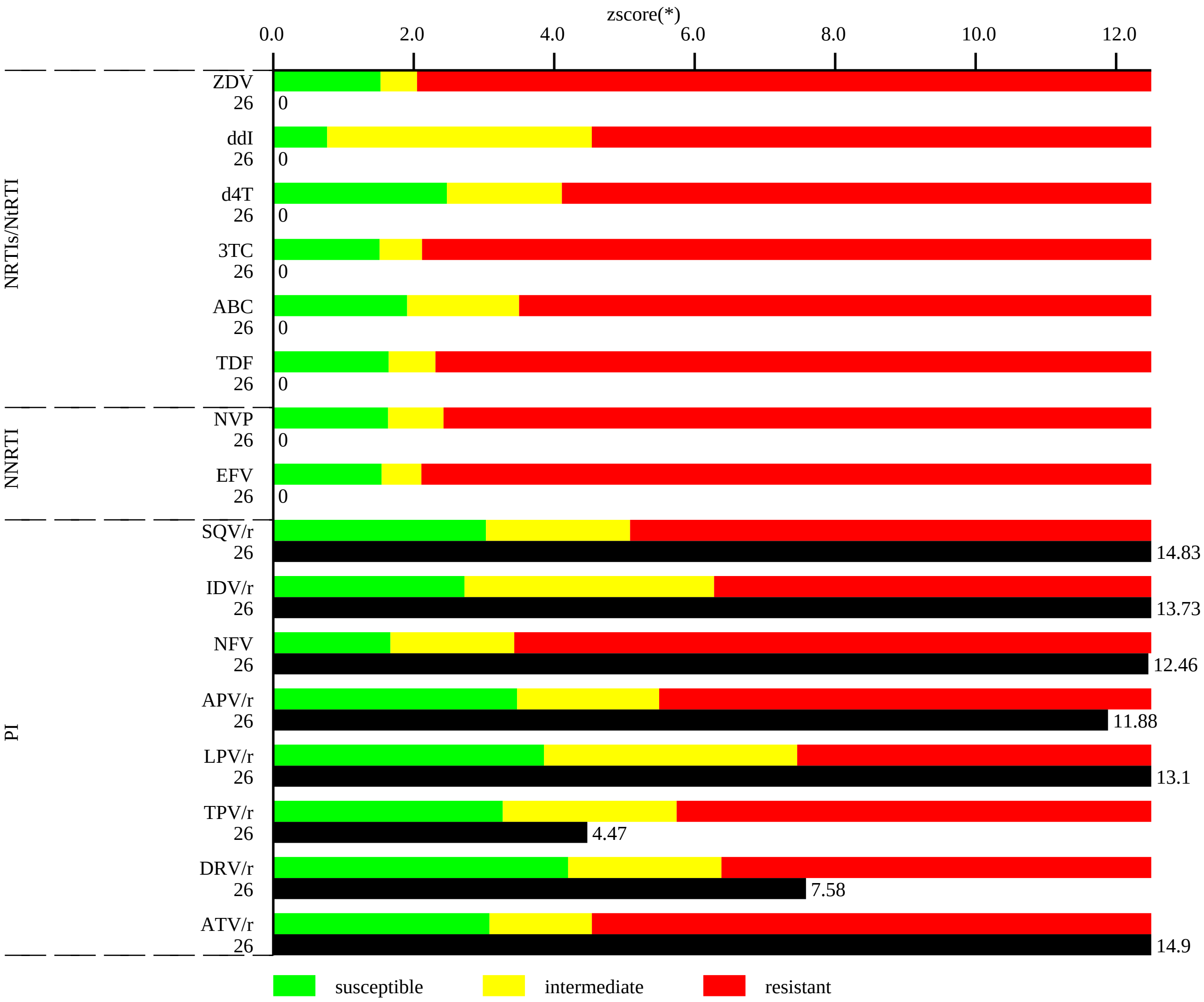

(*)number of standard deviations above mean of drug naive patients. Negative z-scores may indicate hypersusceptibility.

NRTIs/NtRTI:

NNRTIs:

PIs:

Previous genotypes:

date

signature
