# Supplementary material for: A Computational Approach for the Prediction of HIV Resistance Based on Amino Acid and Nucleotide Descriptors
Source: Molecules. 2018 Oct 24;23(11):2751. doi: 10.3390/molecules23112751 (PMC6278491; doi:10.3390/molecules23112751)
Supplement: Supplementary file 1 [file molecules-23-02751-s001.zip › Geno2Pheno-test-set-Predictions-PDF/geno2pheno-results/27.pdf]

geno2pheno®

phenotype prediction from genotype (version 3.4)

I. General information

Patient:	Study Id:
Birth date:	Viral load:
Sample received:	Sample collected:
Sample ID: 27	Predicted subtype: B (100%)
Sample type:	Report date: October 6, 2018
Physician:	Reported by:

II. Substitutions
(relative to the reference strain HXB2)

Protease:	V3I, L10F, I13V, K20I, L24I, M36I, S37N, M46I, I54V, D60E, L63P, I64V, I66V, A71T, I72T, G73C, I84V, T91S
Reverse transcriptase:	

III. Phenotype prediction

Drug	Resistance Factor RF (*)	z-score	Scored Positions (**)
ZDV	0	0	
ddI	0	0	
d4T	0	0	
3TC	0	0	
ABC	0	0	
TDF	0	0	
NVP	0	0	
EFV	0	0	
ETR (***)	Susceptible		
RPV (***)	Susceptible		
SQV	46.656	11.028	48G 84V 90L 11V 74T 88N 53F 95C 26T 1P 80T 34E 47I 97L 78G
IDV	107.585	12.12	82V 88N 29D 1P 84V 71T 21E 65E 46I 24I 11V 85I 30D 90L 78G
NFV	88.356	9.988	88N 30D 84V 82V 97L 68G 90L 46I 31T 75V 74T 5L 2Q 1P 29D
APV	29.255	10.33	76L 50I 84V 32V 46I 85I 10F 22A 63P 1P 47I 82V 97L 45K 21E
LPV	66.308	12.268	82V 50I 46I 54V 84V 76L 22A 10F 7Q 63P 25D 47I 92Q 90L 2Q
TPV	2.839	2.237	69H 46I 48G 84V 33L 47I 91S 15I 90L 82V 74T 24I 88N 58Q 19L
DRV	18.124	7.599	47I 84V 33L 76L 74T 43K 65E 10F 48G 72T 93I 15I 7Q 8R 16G
ATV	54.029	11.704	48G 84V 82V 71T 4T 88N 90L 7Q 76L 45K 32V 46I 53F 41R 2Q

(*) based on LIBSVM, Copyright (c) 2000, Chih-Chung Chang and Chih-Jen Lin

(**) Positions are ordered according to their impact on the phenotype prediction. Differences with respect to HXB2 are underlined. Positions shown in red and in green contribute to an increase or decrease in resistance, repectively. At most 15 positions are shown for each drug.

(***) Resistance predictions and scored mutations for ETR and RPV were performed with rules-based drug-resistance interpretation models by HIV-GRADE (http://www.hiv-grade.de)

geno2pheno®

phenotype prediction from genotype (version 3.4)

IV. Interpretation

Patient:	Birth date:	Sampling date:
Current therapy:		Viral load:

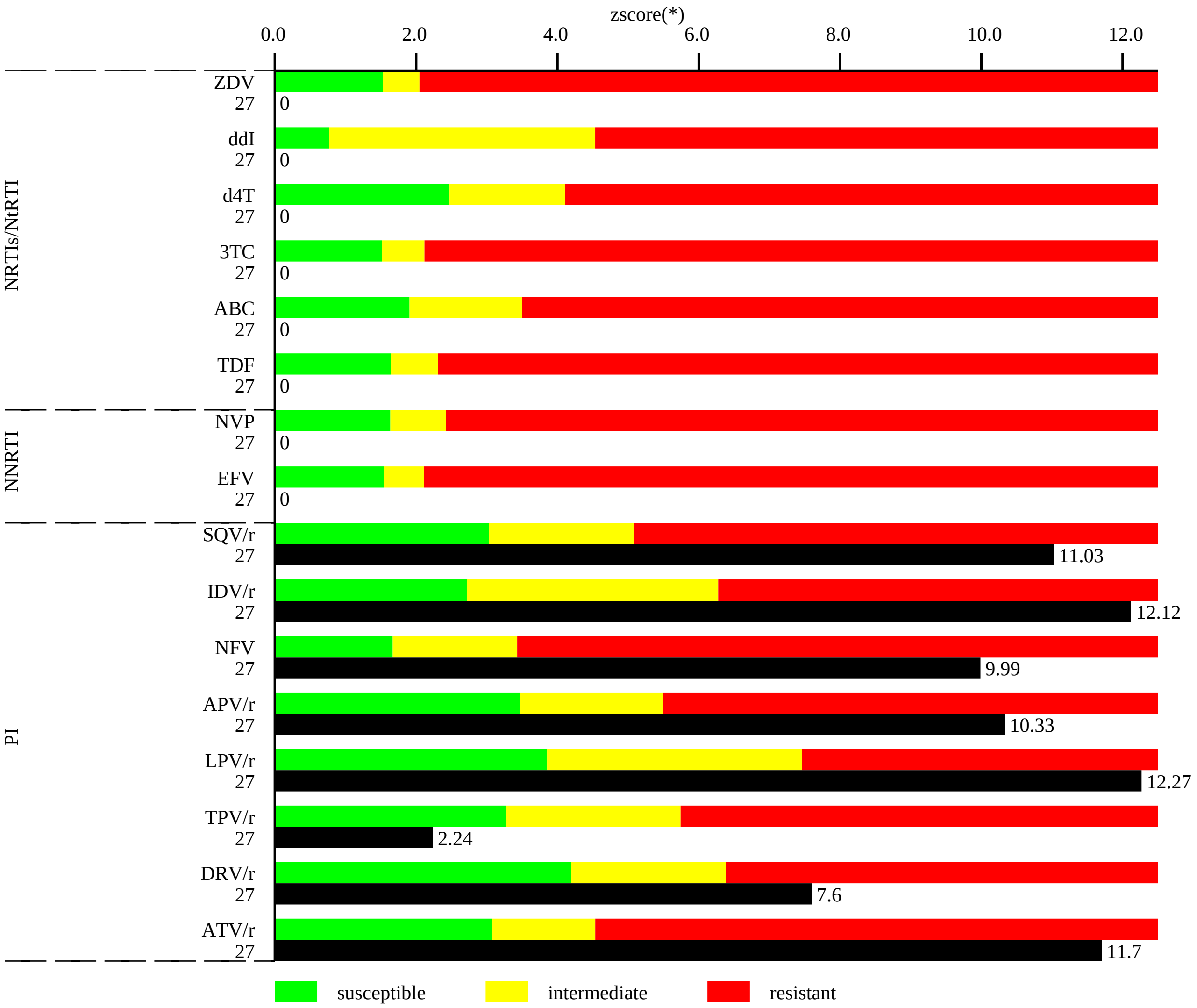

(*)number of standard deviations above mean of drug naive patients. Negative z-scores may indicate hypersusceptibility.

NRTIs/NtRTI:

NNRTIs:

PIs:

Previous genotypes:

date

signature
