# Supplementary material for: A Computational Approach for the Prediction of HIV Resistance Based on Amino Acid and Nucleotide Descriptors
Source: Molecules. 2018 Oct 24;23(11):2751. doi: 10.3390/molecules23112751 (PMC6278491; doi:10.3390/molecules23112751)
Supplement: Supplementary file 1 [file molecules-23-02751-s001.zip › Geno2Pheno-test-set-Predictions-PDF/geno2pheno-results/28.pdf]

geno2pheno®

phenotype prediction from genotype (version 3.4)

I. General information

Patient:	Study Id:
Birth date:	Viral load:
Sample received:	Sample collected:
Sample ID: 28	Predicted subtype: B (100%)
Sample type:	Report date: October 6, 2018
Physician:	Reported by:

II. Substitutions
(relative to the reference strain HXB2)

Protease:	V3I, L10F, I13V, K14R, I15V, K20T, D30N, L33F, E35D, M36I, S37N, R41I, K43R/S, I54L, R57K, I62V, L63P, I66V, A71V, I72T, V77I, N88D, L90M, Q92K, I93L, C95F, T96S
Reverse transcriptase:	

III. Phenotype prediction

Drug	Resistance Factor RF (*)	z-score	Scored Positions (**)
ZDV	0	0	
ddI	0	0	
d4T	0	0	
3TC	0	0	
ABC	0	0	
TDF	0	0	
NVP	0	0	
EFV	0	0	
ETR (***)	Susceptible		
RPV (***)	Susceptible		
SQV	38.283	10.453	48G 73G 84I 11V 74T 88D 90M 53F 26T 1P 80T 34E 30N 54L 47I
IDV	28.693	8.545	82V 54L 46M 90M 29D 1P 73G 21E 65E 84I 11V 85I 78G 98N 76L
NFV	772.515	15.094	30N 90M 46M 82V 97L 73G 68G 88D 31T 75V 74T 54L 84I 5L 2Q
APV	16.223	8.465	33F 76L 50I 84I 46M 32V 54L 85I 10F 22A 63P 1P 47I 82V 90M
LPV	10.761	7.004	82V 46M 84I 50I 33F 76L 54L 22A 10F 7Q 63P 24L 25D 47I 2Q
TPV	7.778	4.423	69H 48G 84I 90M 47I 14R 91T 71V 15V 54L 82V 74T 46M 58Q 19L
DRV	5.254	4.266	47I 33F 84I 76L 74T 73G 46M 65E 10F 14R 93L 48G 72T 7Q 62V
ATV	30.366	9.922	48G 73G 84I 82V 90M 4T 33F 7Q 46M 24L 76L 45K 32V 54L 20T

(*) based on LIBSVM, Copyright (c) 2000, Chih-Chung Chang and Chih-Jen Lin

(**) Positions are ordered according to their impact on the phenotype prediction. Differences with respect to HXB2 are underlined. Positions shown in red and in green contribute to an increase or decrease in resistance, repectively. At most 15 positions are shown for each drug.

(***) Resistance predictions and scored mutations for ETR and RPV were performed with rules-based drug-resistance interpretation models by HIV-GRADE (http://www.hiv-grade.de)

geno2pheno®

phenotype prediction from genotype (version 3.4)

IV. Interpretation

Patient:	Birth date:	Sampling date:
Current therapy:		Viral load:

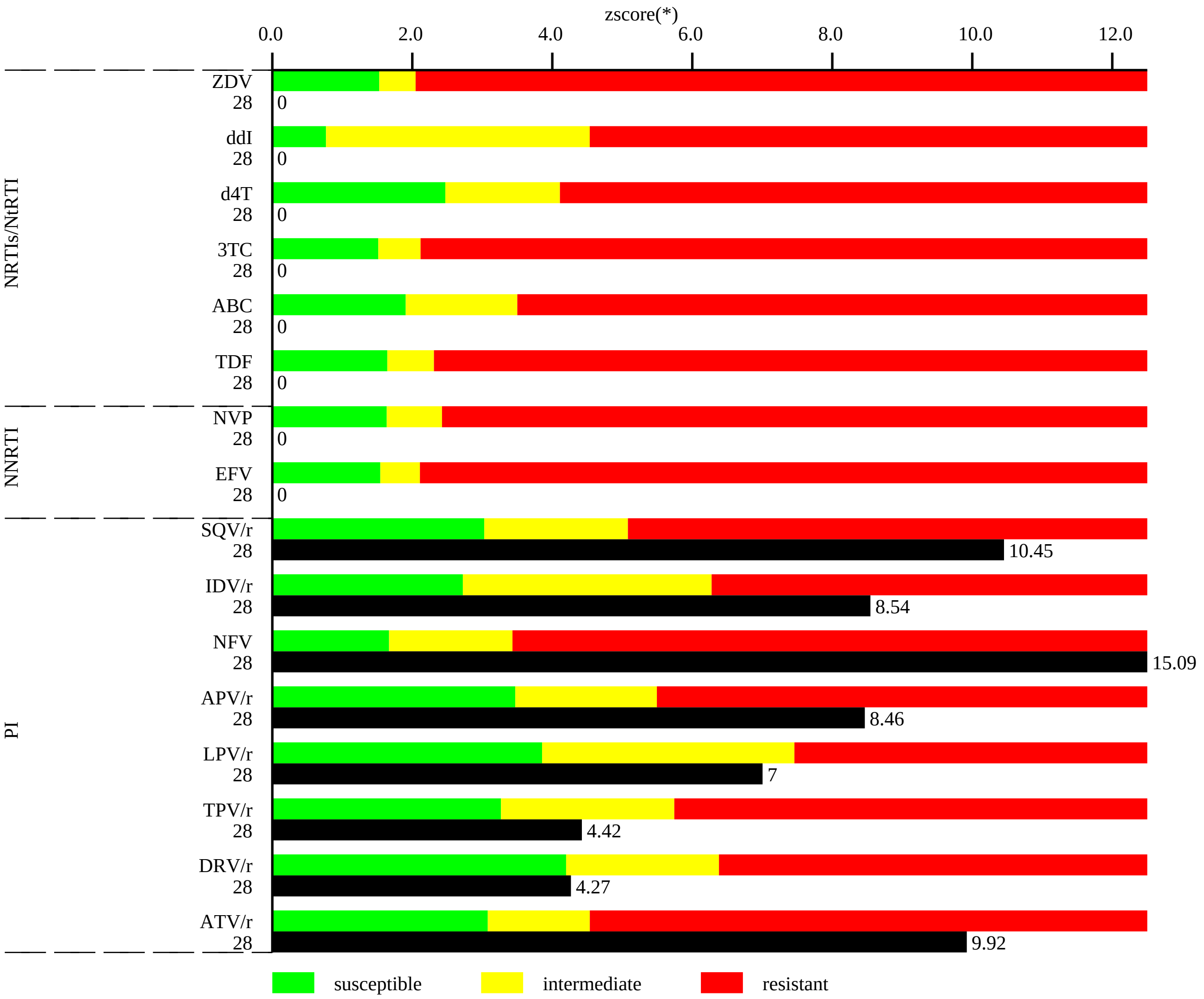

(*)number of standard deviations above mean of drug naive patients. Negative z-scores may indicate hypersusceptibility.

NRTIs/NtRTI:

NNRTIs:

PIs:

Previous genotypes:

date

signature
