# Supplementary material for: A Computational Approach for the Prediction of HIV Resistance Based on Amino Acid and Nucleotide Descriptors
Source: Molecules. 2018 Oct 24;23(11):2751. doi: 10.3390/molecules23112751 (PMC6278491; doi:10.3390/molecules23112751)
Supplement: Supplementary file 1 [file molecules-23-02751-s001.zip › Geno2Pheno-test-set-Predictions-PDF/geno2pheno-results/29.pdf]

geno2pheno®

phenotype prediction from genotype (version 3.4)

I. General information

Patient:	Study Id:
Birth date:	Viral load:
Sample received:	Sample collected:
Sample ID: 29	Predicted subtype: B (100%)
Sample type:	Report date: October 6, 2018
Physician:	Reported by:

II. Substitutions
(relative to the reference strain HXB2)

Protease:	V3I, L10F/I, V11I/V, I13V, I15V, Q18H/Q, K20I, M36I, S37D, M46L, I54M, L63P, A71I/V, V82A, L90M, I93L
Reverse transcriptase:	

III. Phenotype prediction

Drug	Resistance Factor RF (*)	z-score	Scored Positions (**)
ZDV	0	0	
ddI	0	0	
d4T	0	0	
3TC	0	0	
ABC	0	0	
TDF	0	0	
NVP	0	0	
EFV	0	0	
ETR (***)	Susceptible		
RPV (***)	Susceptible		
SQV	28.511	9.596	48G 73G 84I 11V 74T 88N 90M 53F 95C 26T 1P 80T 34E 47I 97L
IDV	188.193	13.633	88N 90M 29D 1P 73G 21E 65E 84I 11V 10I 85I 30D 78G 98N 76L
NFV	203.72	11.955	88N 30D 90M 97L 73G 68G 31T 75V 74T 84I 5L 18H 2Q 1P 29D
APV	35.192	10.915	76L 50I 84I 54M 32V 85I 37D 10F 22A 63P 1P 47I 90M 97L 45K
LPV	63.44	12.14	84I 50I 76L 22A 10F 7Q 63P 24L 25D 82A 54M 37D 47I 92Q 2Q
TPV	9.227	4.793	69H 82A 48G 84I 33L 90M 47I 72I 91T 71V 15V 74T 88N 58Q 19L
DRV	4.317	3.738	47I 84I 33L 76L 74T 43K 73G 54M 37D 65E 10F 93L 48G 82A 7Q
ATV	119.762	14.167	48G 73G 84I 90M 82A 4T 46L 88N 7Q 24L 76L 54M 45K 32V 93L

(*) based on LIBSVM, Copyright (c) 2000, Chih-Chung Chang and Chih-Jen Lin

(**) Positions are ordered according to their impact on the phenotype prediction. Differences with respect to HXB2 are underlined. Positions shown in red and in green contribute to an increase or decrease in resistance, repectively. At most 15 positions are shown for each drug.

(***) Resistance predictions and scored mutations for ETR and RPV were performed with rules-based drug-resistance interpretation models by HIV-GRADE (http://www.hiv-grade.de)

geno2pheno®

phenotype prediction from genotype (version 3.4)

IV. Interpretation

Patient:	Birth date:	Sampling date:
Current therapy:		Viral load:

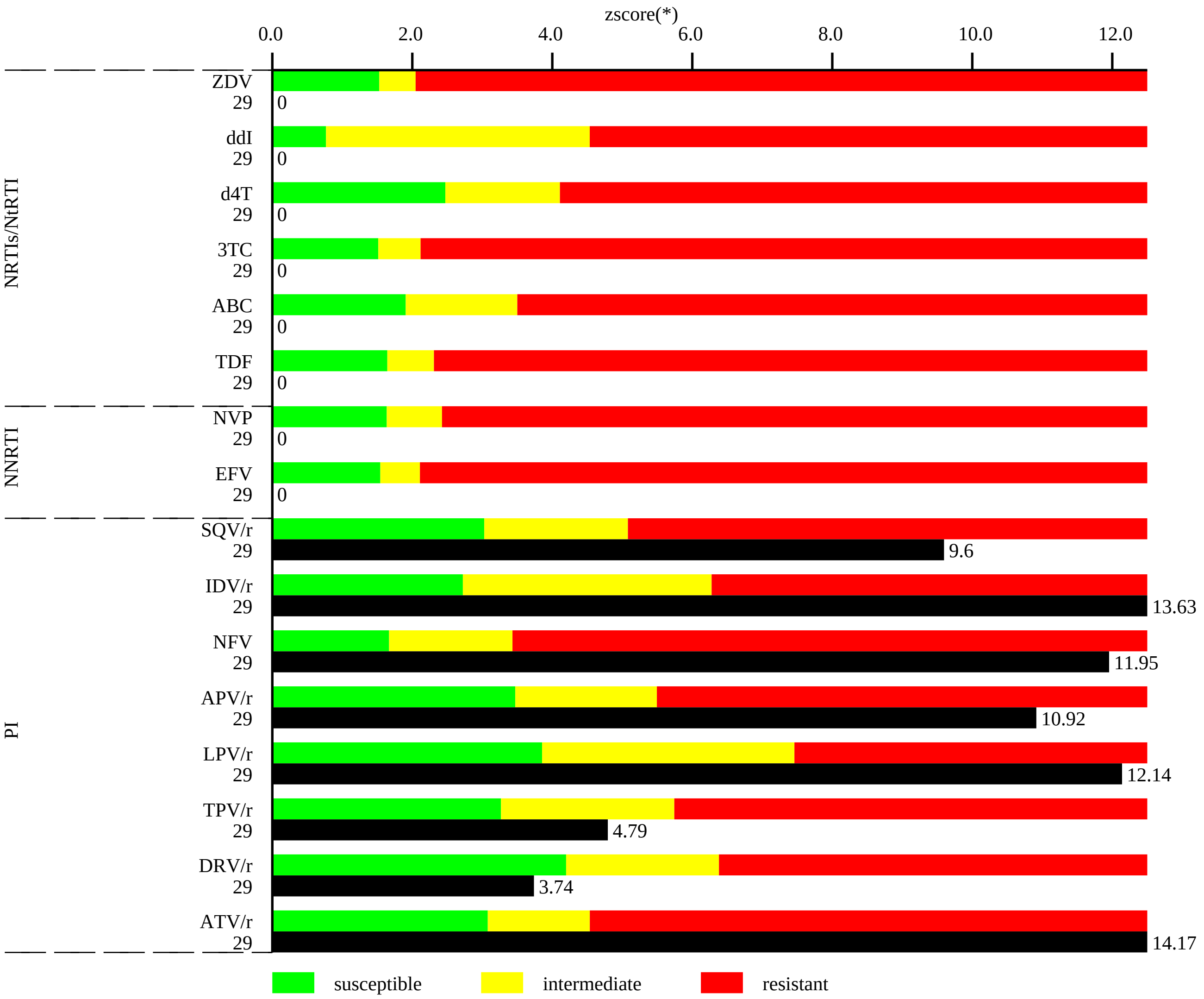

(*)number of standard deviations above mean of drug naive patients. Negative z-scores may indicate hypersusceptibility.

NRTIs/NtRTI:

NNRTIs:

PIs:

Previous genotypes:

date

signature
