# Supplementary material for: A Computational Approach for the Prediction of HIV Resistance Based on Amino Acid and Nucleotide Descriptors
Source: Molecules. 2018 Oct 24;23(11):2751. doi: 10.3390/molecules23112751 (PMC6278491; doi:10.3390/molecules23112751)
Supplement: Supplementary file 1 [file molecules-23-02751-s001.zip › Geno2Pheno-test-set-Predictions-PDF/geno2pheno-results/3.pdf]

geno2pheno®

phenotype prediction from genotype (version 3.4)

I. General information

Patient:	Study Id:
Birth date:	Viral load:
Sample received:	Sample collected:
Sample ID: 3	Predicted subtype: B (100%)
Sample type:	Report date: October 6, 2018
Physician:	Reported by:

II. Substitutions

(relative to the reference strain HXB2)

Protease:	E35D, S37D, R41K/R, D60E
Reverse transcriptase:	

III. Phenotype prediction

Drug	Resistance Factor RF (*)	z-score	Scored Positions (**)
ZDV	0	0	
ddI	0	0	
d4T	0	0	
3TC	0	0	
ABC	0	0	
TDF	0	0	
NVP	0	0	
EFV	0	0	
ETR (***)	Susceptible		
RPV (***)	Susceptible		
SQV	0.794	-0.816	48G 73G 90L 84I 54I 11V 74T 88N 53F 95C 26T 71A 80T 34E 47I
IDV	1.093	-0.294	54I 82V 88N 46M 29D 73G 21E 65E 84I 11V 71A 85I 30D 90L 78G
NFV	1.576	0.505	88N 54I 30D 46M 82V 97L 20K 73G 68G 90L 71A 36M 31T 75V 74T
APV	1.322	0.53	54I 76L 50I 84I 46M 32V 85I 37D 22A 47I 13I 82V 97L 45K 21E
LPV	1.22	0.702	54I 82V 46M 84I 50I 76L 10L 22A 71A 7Q 24L 20K 25D 37D 47I
TPV	1.436	0.759	69H 48G 84I 33L 54I 47I 71A 72I 15I 91T 20K 90L 82V 74T 46M
DRV	1.276	0.458	47I 84I 54I 33L 76L 74T 43K 73G 46M 71A 36M 37D 65E 48G 93I
ATV	1.273	0.108	54I 48G 73G 84I 82V 4T 71A 88N 90L 7Q 46M 36M 20K 24L 76L

(*) based on LIBSVM, Copyright (c) 2000, Chih-Chung Chang and Chih-Jen Lin

(**) Positions are ordered according to their impact on the phenotype prediction. Differences with respect to HXB2 are underlined. Positions shown in red and in green contribute to an increase or decrease in resistance, repectively. At most 15 positions are shown for each drug.

(***) Resistance predictions and scored mutations for ETR and RPV were performed with rules-based drug-resistance interpretation models by HIV-GRADE (http://www.hiv-grade.de)

geno2pheno®

phenotype prediction from genotype (version 3.4)

IV. Interpretation

Patient:	Birth date:	Sampling date:
Current therapy:		Viral load:

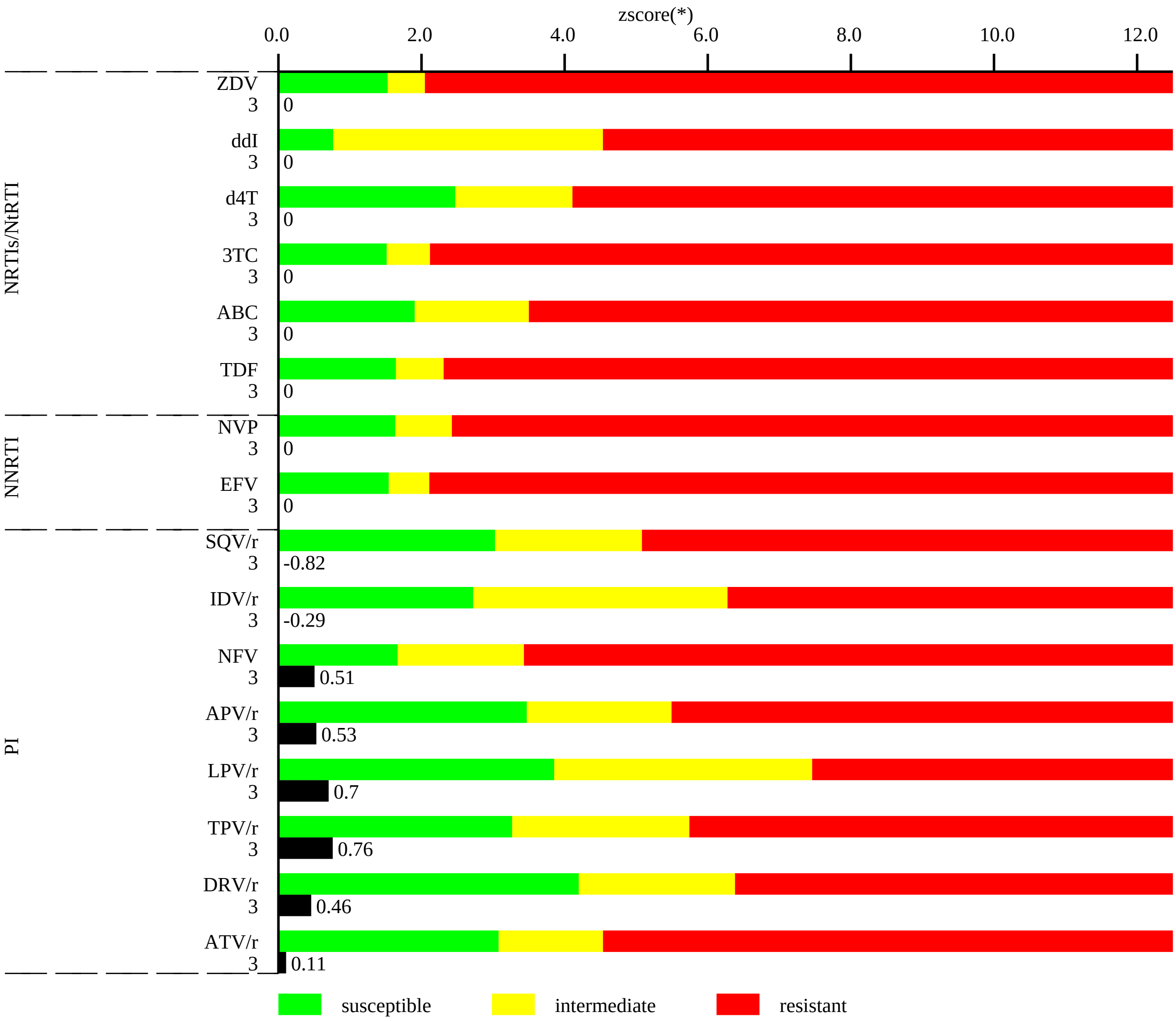

(*)number of standard deviations above mean of drug naive patients. Negative z-scores may indicate hypersusceptibility.

NRTIs/NtRTI:

NNRTIs:

PIs:

Previous genotypes:

date

signature
