# Supplementary material for: A Computational Approach for the Prediction of HIV Resistance Based on Amino Acid and Nucleotide Descriptors
Source: Molecules. 2018 Oct 24;23(11):2751. doi: 10.3390/molecules23112751 (PMC6278491; doi:10.3390/molecules23112751)
Supplement: Supplementary file 1 [file molecules-23-02751-s001.zip › Geno2Pheno-test-set-Predictions-PDF/geno2pheno-results/30.pdf]

geno2pheno®

phenotype prediction from genotype (version 3.4)

I. General information

Patient:		Study Id:	
Birth date:		Viral load:	
Sample received:		Sample collected:	
Sample ID:	30	Predicted subtype:	B (100%)
Sample type:		Report date:	October 6, 2018
Physician:		Reported by:	

II. Substitutions

(relative to the reference strain HXB2)

Protease:	V3I, L10F, I13V, D30N, L33F, S37N, K45R, I54L, L63P, I66I/V, P79P/S, I84V, N88D, L89F/L, L90M, I93L, C95C/F
Reverse transcriptase:	

III. Phenotype prediction

Drug	Resistance Factor RF (*)	z-score	Scored Positions (**)
ZDV	0	0	
ddI	0	0	
d4T	0	0	
3TC	0	0	
ABC	0	0	
TDF	0	0	
NVP	0	0	
EFV	0	0	
ETR (***)	Susceptible		
RPV (***)	Susceptible		
SQV	98.63	13.205	48G 84V 73G 11V 74T 88D 90M 53F 26T 1P 71A 80T 34E 30N 54L
IDV	26.999	8.38	82V 54L 46M 90M 29D 1P 84V 73G 21E 65E 11V 71A 85I 78G 98N
NFV	357.229	13.277	30N 90M 46M 84V 82V 97L 20K 73G 68G 88D 71A 36M 31T 75V 74T
APV	69.706	13.078	33F 76L 50I 84V 46M 32V 54L 85I 10F 22A 63P 1P 47I 82V 90M
LPV	14.349	7.837	82V 46M 50I 84V 33F 76L 54L 22A 71A 10F 7Q 63P 24L 20K 25D
TPV	3.152	2.464	69H 48G 84V 90M 47I 71A 72I 15I 91T 20K 54L 82V 74T 46M 58Q
DRV	5.896	4.577	47I 33F 84V 76L 74T 43K 73G 46M 71A 36M 65E 10F 93L 48G 15I
ATV	39.085	10.703	48G 73G 84V 82V 90M 4T 33F 71A 7Q 46M 36M 20K 24L 76L 32V

(*) based on LIBSVM, Copyright (c) 2000, Chih-Chung Chang and Chih-Jen Lin

(**) Positions are ordered according to their impact on the phenotype prediction. Differences with respect to HXB2 are underlined. Positions shown in red and in green contribute to an increase or decrease in resistance, repectively. At most 15 positions are shown for each drug.

(***) Resistance predictions and scored mutations for ETR and RPV were performed with rules-based drug-resistance interpretation models by HIV-GRADE (http://www.hiv-grade.de)

geno2pheno®

phenotype prediction from genotype (version 3.4)

IV. Interpretation

Patient:	Birth date:	Sampling date:
Current therapy:		Viral load:

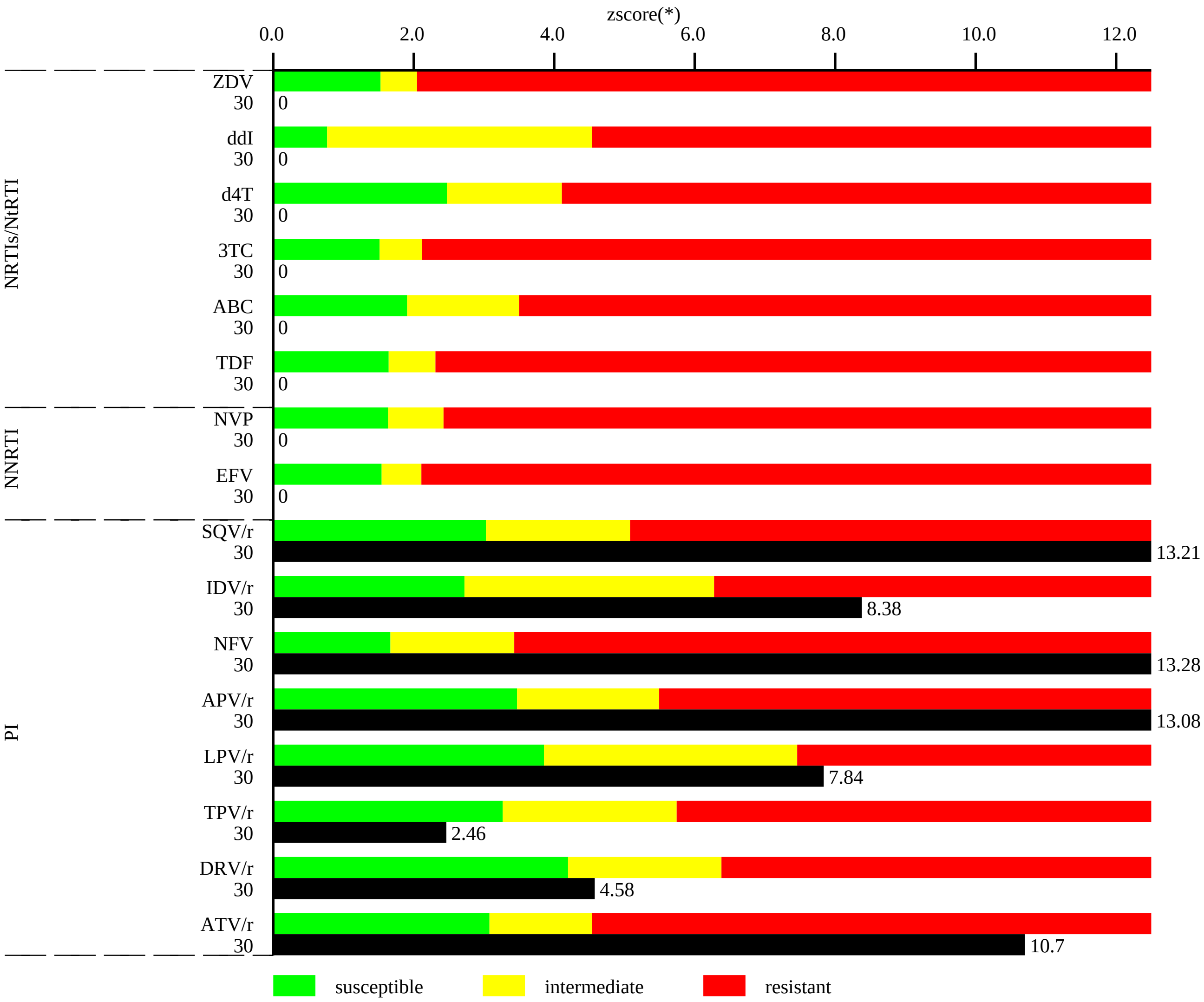

(*)number of standard deviations above mean of drug naive patients. Negative z-scores may indicate hypersusceptibility.

NRTIs/NtRTI:

NNRTIs:

PIs:

Previous genotypes:

date

signature
