# Supplementary material for: A Computational Approach for the Prediction of HIV Resistance Based on Amino Acid and Nucleotide Descriptors
Source: Molecules. 2018 Oct 24;23(11):2751. doi: 10.3390/molecules23112751 (PMC6278491; doi:10.3390/molecules23112751)
Supplement: Supplementary file 1 [file molecules-23-02751-s001.zip › Geno2Pheno-test-set-Predictions-PDF/geno2pheno-results/31.pdf]

geno2pheno®

phenotype prediction from genotype (version 3.4)

I. General information

Patient:		Study Id:	
Birth date:		Viral load:	
Sample received:		Sample collected:	
Sample ID:	31	Predicted subtype:	B (100%)
Sample type:		Report date:	October 6, 2018
Physician:		Reported by:	

II. Substitutions

(relative to the reference strain HXB2)

Protease:	V3I, T4A, L10I, I13V, I15V, K20I, E35D/N, M36I, S37N, M46I, I54V, R57K, Q58E, D60E, Q61N, I62V, L63P, I72T, G73C, T74S, V82F, L90M, Q92K
Reverse transcriptase:	

III. Phenotype prediction

Drug	Resistance Factor RF (*)	z-score	Scored Positions (**)
ZDV	0	0	
ddI	0	0	
d4T	0	0	
3TC	0	0	
ABC	0	0	
TDF	0	0	
NVP	0	0	
EFV	0	0	
ETR (***)	Susceptible		
RPV (***)	Susceptible		
SQV	36.028	10.277	48G 84I 11V 88N 82F 90M 53F 95C 26T 1P 71A 80T 34E 47I 97L
IDV	409.699	15.737	88N 82F 90M 29D 1P 21E 65E 84I 46I 11V 71A 10I 85I 30D 78G
NFV	854.596	15.331	88N 30D 90M 97L 68G 71A 82F 46I 31T 75V 84I 5L 2Q 1P 29D
APV	76.179	13.359	76L 50I 84I 82F 32V 46I 85I 22A 63P 1P 47I 90M 97L 45K 10I
LPV	98.114	13.402	84I 50I 46I 54V 76L 82F 22A 71A 7Q 63P 24L 25D 74S 47I 2Q
TPV	22.128	6.69	69H 46I 48G 84I 33L 90M 47I 71A 91T 15V 74S 88N 19L 39P 89L
DRV	13.065	6.718	47I 84I 33L 76L 43K 74S 71A 65E 48G 72T 93I 7Q 62V 8R 16G
ATV	82.487	13.014	48G 84I 90M 71A 88N 7Q 24L 76L 45K 32V 46I 53F 41R 2Q 73C

(*) based on LIBSVM, Copyright (c) 2000, Chih-Chung Chang and Chih-Jen Lin

(**) Positions are ordered according to their impact on the phenotype prediction. Differences with respect to HXB2 are underlined. Positions shown in red and in green contribute to an increase or decrease in resistance, repectively. At most 15 positions are shown for each drug.

(***) Resistance predictions and scored mutations for ETR and RPV were performed with rules-based drug-resistance interpretation models by HIV-GRADE (http://www.hiv-grade.de)

geno2pheno®

phenotype prediction from genotype (version 3.4)

IV. Interpretation

Patient:	Birth date:	Sampling date:
Current therapy:		Viral load:

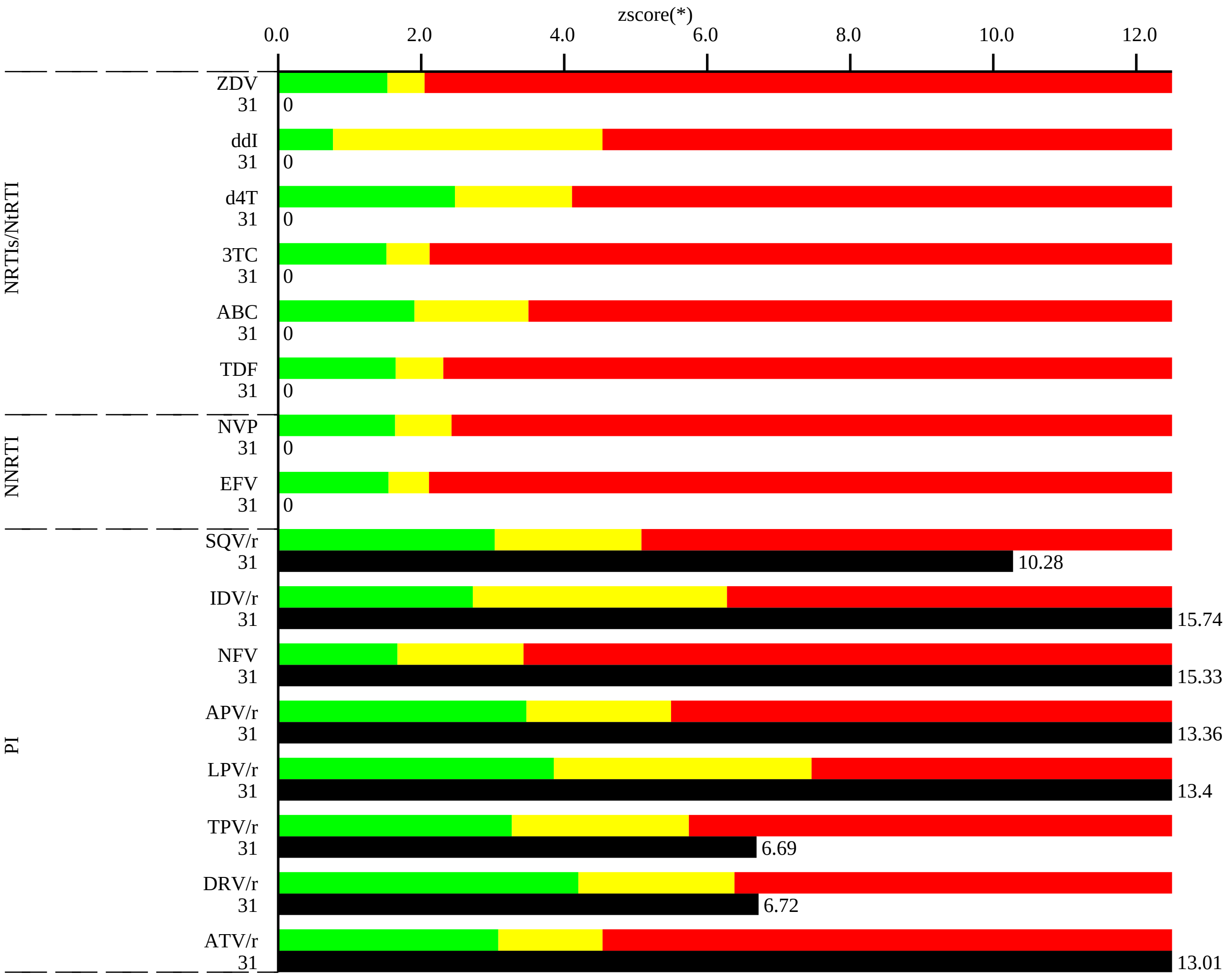

susceptible intermediate resistant

(*)number of standard deviations above mean of drug naive patients. Negative z-scores may indicate hypersusceptibility.

NRTIs/NtRTI:

NNRTIs:

PIs:

Previous genotypes:

date

signature
