# Supplementary material for: A Computational Approach for the Prediction of HIV Resistance Based on Amino Acid and Nucleotide Descriptors
Source: Molecules. 2018 Oct 24;23(11):2751. doi: 10.3390/molecules23112751 (PMC6278491; doi:10.3390/molecules23112751)
Supplement: Supplementary file 1 [file molecules-23-02751-s001.zip › Geno2Pheno-test-set-Predictions-PDF/geno2pheno-results/32.pdf]

geno2pheno®

phenotype prediction from genotype (version 3.4)

I. General information

Patient:		Study Id:	
Birth date:		Viral load:	
Sample received:		Sample collected:	
Sample ID:	32	Predicted subtype:	B (100%)
Sample type:		Report date:	October 6, 2018
Physician:		Reported by:	

II. Substitutions

(relative to the reference strain HXB2)

Protease:	V3I, L10I, V11I, T12K, I13V, K20V, V32I, L33F/L, E35G, M36I, S37D/N, M46I, I47V, I54M, R57K, Q58E, L63P, I64V, A71I/V, G73S, I84I/V, L89V, L90M
Reverse transcriptase:	

III. Phenotype prediction

Drug	Resistance Factor RF (*)	z-score	Scored Positions (**)
ZDV	0	0	
ddI	0	0	
d4T	0	0	
3TC	0	0	
ABC	0	0	
TDF	0	0	
NVP	0	0	
EFV	0	0	
ETR (***)	Susceptible		
RPV (***)	Susceptible		
SQV	141.209	14.249	48G 84V 74T 88N 90M 47V 53F 95C 26T 11I 1P 80T 35G 34E 97L
IDV	315.352	15.029	82V 88N 90M 29D 1P 84V 21E 65E 20V 32I 46I 10I 85I 30D 73S
NFV	665.846	14.744	88N 30D 90M 84V 82V 97L 68G 46I 31T 75V 74T 5L 2Q 1P 29D
APV	1801.621	23.369	33F 76L 50I 84V 54M 32I 89V 46I 85I 47V 37D 22A 63P 1P 82V
LPV	221.878	15.764	82V 50I 46I 84V 33F 76L 89V 22A 7Q 63P 24L 47V 25D 54M 37D
TPV	27.664	7.174	69H 46I 48G 84V 90M 47V 72I 15I 91T 71V 73S 82V 74T 88N 19L
DRV	149.725	13.281	47V 33F 84V 76L 74T 43K 54M 37D 65E 48G 32I 93I 15I 7Q 8R
ATV	468.782	18.389	48G 84V 82V 90M 4T 33F 88N 35G 7Q 73S 24L 76L 54M 45K 32I

(*) based on LIBSVM, Copyright (c) 2000, Chih-Chung Chang and Chih-Jen Lin

(**) Positions are ordered according to their impact on the phenotype prediction. Differences with respect to HXB2 are underlined. Positions shown in red and in green contribute to an increase or decrease in resistance, repectively. At most 15 positions are shown for each drug.

(***) Resistance predictions and scored mutations for ETR and RPV were performed with rules-based drug-resistance interpretation models by HIV-GRADE (http://www.hiv-grade.de)

geno2pheno®

phenotype prediction from genotype (version 3.4)

IV. Interpretation

Patient:	Birth date:	Sampling date:
Current therapy:		Viral load:

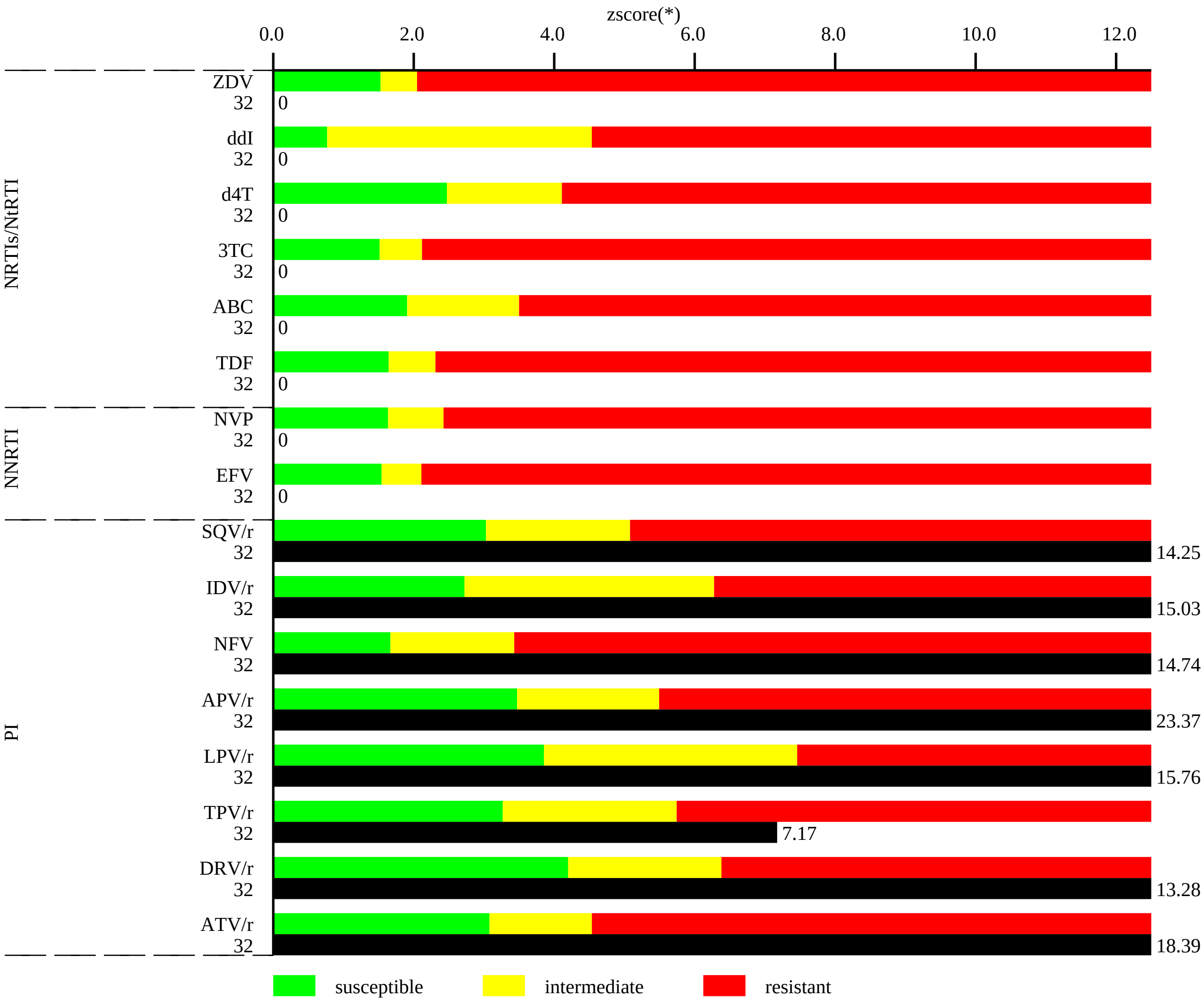

(*)number of standard deviations above mean of drug naive patients. Negative z-scores may indicate hypersusceptibility.

NRTIs/NtRTI:

NNRTIs:

PIs:

Previous genotypes:

date

signature
