# Supplementary material for: A Computational Approach for the Prediction of HIV Resistance Based on Amino Acid and Nucleotide Descriptors
Source: Molecules. 2018 Oct 24;23(11):2751. doi: 10.3390/molecules23112751 (PMC6278491; doi:10.3390/molecules23112751)
Supplement: Supplementary file 1 [file molecules-23-02751-s001.zip › Geno2Pheno-test-set-Predictions-PDF/geno2pheno-results/33.pdf]

geno2pheno®

phenotype prediction from genotype (version 3.4)

I. General information

Patient:		Study Id:	
Birth date:		Viral load:	
Sample received:		Sample collected:	
Sample ID:	33	Predicted subtype:	B (100%)
Sample type:		Report date:	October 6, 2018
Physician:		Reported by:	

II. Substitutions

(relative to the reference strain HXB2)

Protease:	V3I, L10I, K20R, E35D, M36I, S37D, R41K, M46I, I54V, I62V, L63P, V82A, L90M, I93L, C95F
Reverse transcriptase:	

III. Phenotype prediction

Drug	Resistance Factor RF (*)	z-score	Scored Positions (**)
ZDV	0	0	
ddI	0	0	
d4T	0	0	
3TC	0	0	
ABC	0	0	
TDF	0	0	
NVP	0	0	
EFV	0	0	
ETR (***)	Susceptible		
RPV (***)	Susceptible		
SQV	22.034	8.847	48G 73G 84I 11V 74T 88N 90M 53F 26T 1P 71A 80T 34E 47I 97L
IDV	124.053	12.505	88N 90M 29D 1P 73G 21E 65E 84I 46I 11V 71A 10I 85I 30D 78G
NFV	109.671	10.496	88N 30D 90M 97L 73G 68G 71A 46I 31T 75V 74T 84I 5L 2Q 1P
APV	17.546	8.713	76L 50I 84I 32V 46I 85I 37D 22A 63P 1P 47I 13I 90M 97L 45K
LPV	82.224	12.89	84I 50I 46I 54V 76L 22A 71A 7Q 63P 24L 25D 82A 37D 47I 92Q
TPV	1.176	0.326	69H 82A 46I 48G 84I 33L 90M 47I 71A 72I 15I 91T 74T 88N 58Q
DRV	1.567	1.011	47I 84I 33L 76L 74T 43K 73G 71A 37D 65E 93L 48G 82A 15I 7Q
ATV	39.053	10.7	48G 73G 84I 90M 82A 4T 71A 88N 7Q 24L 76L 45K 32V 41K 93L

(*) based on LIBSVM, Copyright (c) 2000, Chih-Chung Chang and Chih-Jen Lin

(**) Positions are ordered according to their impact on the phenotype prediction. Differences with respect to HXB2 are underlined. Positions shown in red and in green contribute to an increase or decrease in resistance, repectively. At most 15 positions are shown for each drug.

(***) Resistance predictions and scored mutations for ETR and RPV were performed with rules-based drug-resistance interpretation models by HIV-GRADE (http://www.hiv-grade.de)

geno2pheno®

phenotype prediction from genotype (version 3.4)

IV. Interpretation

Patient:	Birth date:	Sampling date:
Current therapy:		Viral load:

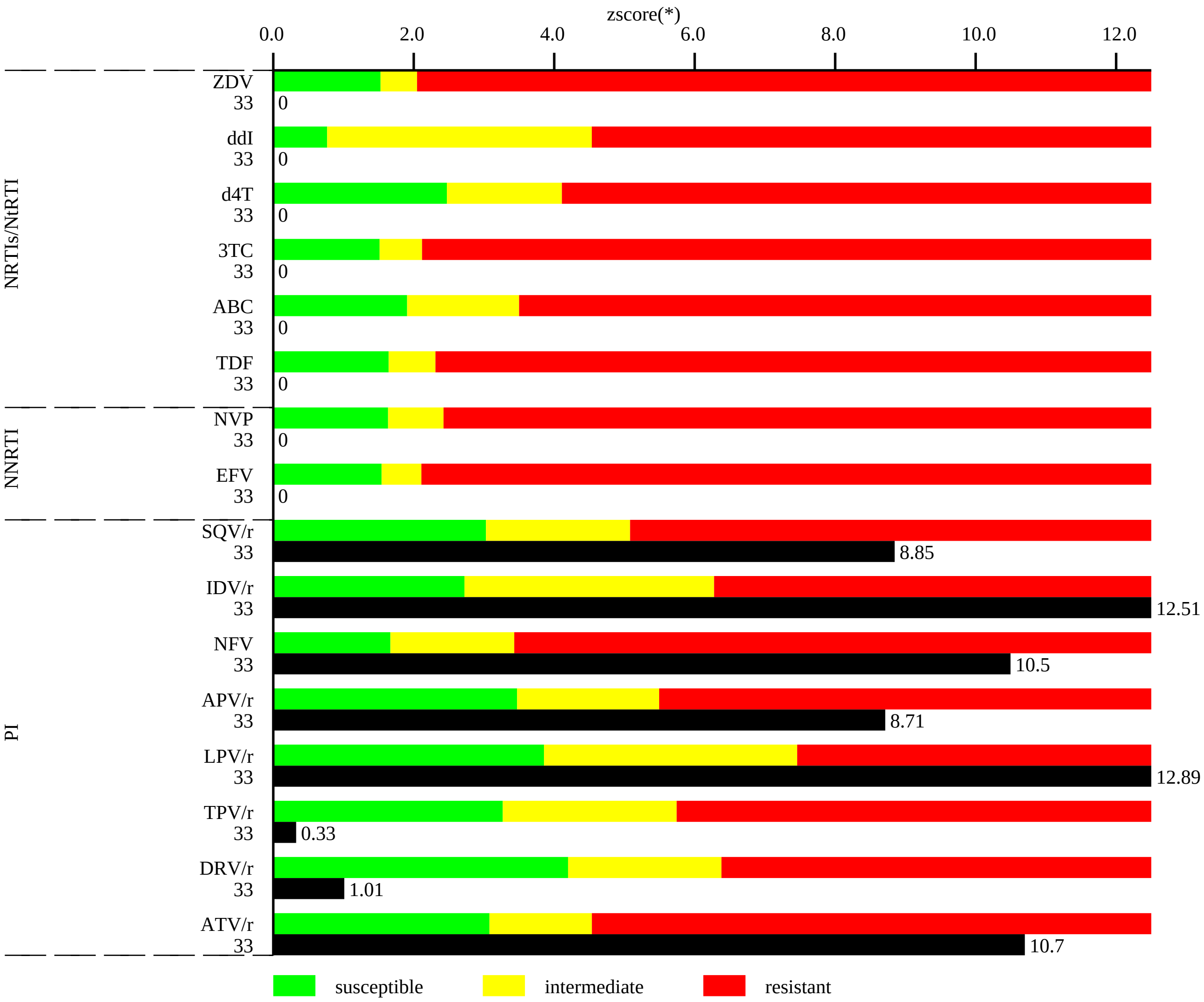

(*)number of standard deviations above mean of drug naive patients. Negative z-scores may indicate hypersusceptibility.

NRTIs/NtRTI:

NNRTIs:

PIs:

Previous genotypes:

date

signature
