# Supplementary material for: A Computational Approach for the Prediction of HIV Resistance Based on Amino Acid and Nucleotide Descriptors
Source: Molecules. 2018 Oct 24;23(11):2751. doi: 10.3390/molecules23112751 (PMC6278491; doi:10.3390/molecules23112751)
Supplement: Supplementary file 1 [file molecules-23-02751-s001.zip › Geno2Pheno-test-set-Predictions-PDF/geno2pheno-results/34.pdf]

geno2pheno®

phenotype prediction from genotype (version 3.4)

I. General information

Patient:	Study Id:
Birth date:	Viral load:
Sample received:	Sample collected:
Sample ID: 34	Predicted subtype: B (100%)
Sample type:	Report date: October 6, 2018
Physician:	Reported by:

II. Substitutions
(relative to the reference strain HXB2)

Protease:	V3I, L10I, I15V, L23I, S37N, M46L, F53L, I54V, I62V, L63V, H69R, A71V, I72K, L76V, V82A, L89M, I93L
Reverse transcriptase:	

III. Phenotype prediction

Drug	Resistance Factor RF (*)	z-score	Scored Positions (**)
ZDV	0	0	
ddI	0	0	
d4T	0	0	
3TC	0	0	
ABC	0	0	
TDF	0	0	
NVP	0	0	
EFV	0	0	
ETR (***)	Susceptible		
RPV (***)	Susceptible		
SQV	5.227	4.663	48G 73G 90L 84I 11V 74T 88N 95C <u>53L</u> 26T 1P 80T 34E <u>63V</u> 47I
IDV	40.03	9.446	88N <u>76V</u> 29D 1P 73G 21E 65E 84I 11V <u>10I</u> 85I 30D 90L <u>89M</u> 78G
NFV	9.95	4.845	88N 30D 97L 20K 73G 68G 90L 36M 31T 75V 74T 84I 5L <u>89M</u> 2Q
APV	31.189	10.533	<u>76V</u> 50I 84I 32V 85I 22A 1P 47I 13I <u>89M</u> 97L 45K <u>10I</u> <u>46L</u> 21E
LPV	41.502	10.911	84I 50I <u>54V</u> <u>76V</u> 22A 7Q 24L 20K 25D <u>82A</u> 47I 92Q 90L 2Q 48G
TPV	0.665	-0.91	<u>82A</u> 48G 84I 33L 47I <u>89M</u> 91T <u>71V</u> 20K <u>15V</u> 90L 74T 88N 58Q 19L
DRV	3.519	3.188	47I 84I 33L 74T 43K 73G <u>76V</u> 36M 65E <u>93L</u> <u>89M</u> 48G <u>82A</u> 7Q <u>62V</u>
ATV	14.061	7.54	48G 73G 84I <u>82A</u> 4T <u>46L</u> <u>76V</u> 88N 90L 7Q 36M 20K 24L 45K 32V

(*) based on LIBSVM, Copyright (c) 2000, Chih-Chung Chang and Chih-Jen Lin

(**) Positions are ordered according to their impact on the phenotype prediction. Differences with respect to HXB2 are underlined. Positions shown in red and in green contribute to an increase or decrease in resistance, repectively. At most 15 positions are shown for each drug.

(***) Resistance predictions and scored mutations for ETR and RPV were performed with rules-based drug-resistance interpretation models by HIV-GRADE (http://www.hiv-grade.de)

geno2pheno®

phenotype prediction from genotype (version 3.4)

IV. Interpretation

Patient:	Birth date:	Sampling date:
Current therapy:		Viral load:

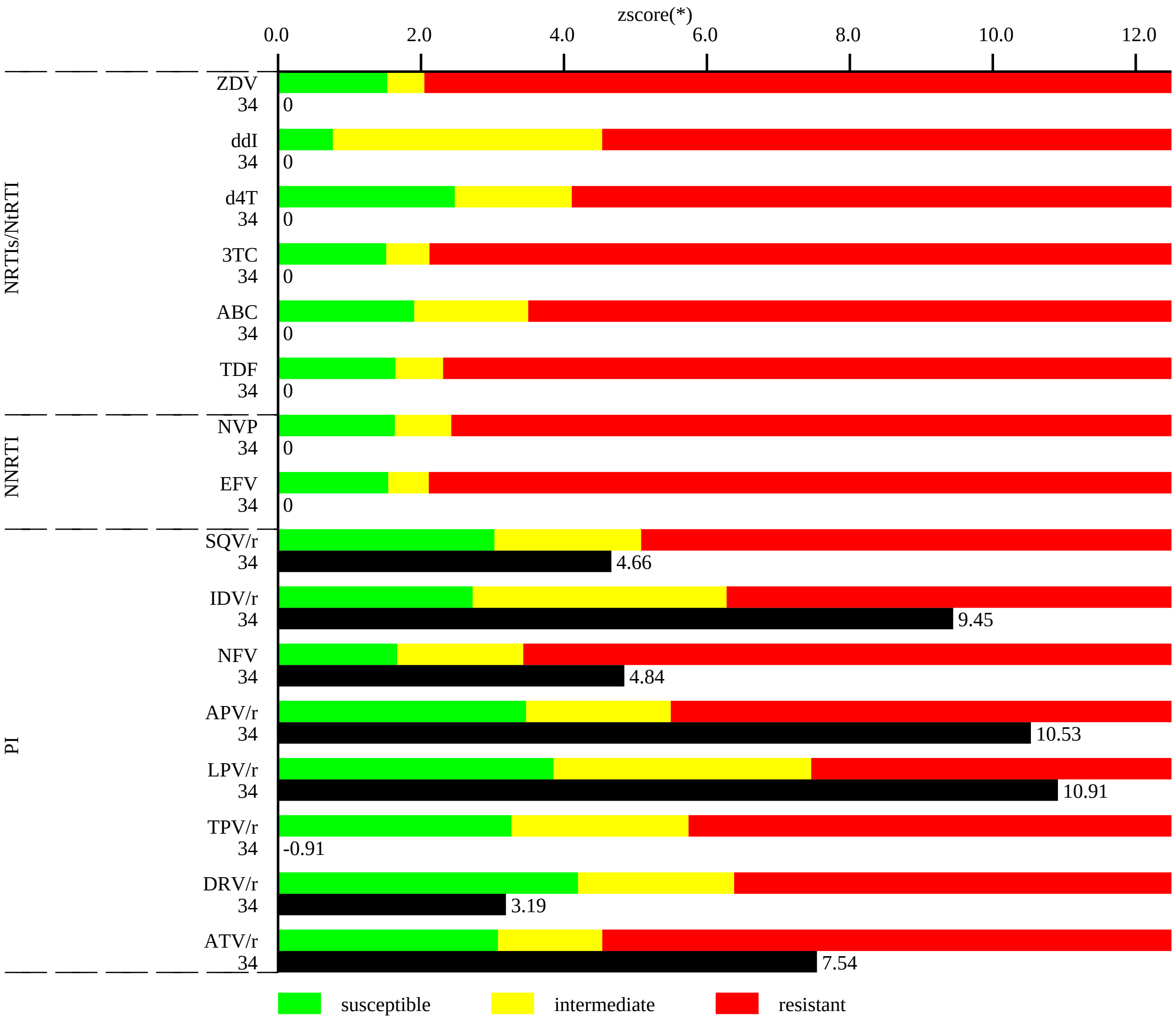

(*)number of standard deviations above mean of drug naive patients. Negative z-scores may indicate hypersusceptibility.

NRTIs/NtRTI:

NNRTIs:

PIs:

Previous genotypes:

date

signature
