# Supplementary material for: A Computational Approach for the Prediction of HIV Resistance Based on Amino Acid and Nucleotide Descriptors
Source: Molecules. 2018 Oct 24;23(11):2751. doi: 10.3390/molecules23112751 (PMC6278491; doi:10.3390/molecules23112751)
Supplement: Supplementary file 1 [file molecules-23-02751-s001.zip › Geno2Pheno-test-set-Predictions-PDF/geno2pheno-results/35.pdf]

geno2pheno®

phenotype prediction from genotype (version 3.4)

I. General information

Patient:		Study Id:	
Birth date:		Viral load:	
Sample received:		Sample collected:	
Sample ID:	35	Predicted subtype:	07_BC (100%)
Sample type:		Report date:	October 6, 2018
Physician:		Reported by:	

II. Substitutions

(relative to the reference strain HXB2)

Protease:	V3I, L10I, L33F, S37N, G48V, I54S, Q58E/Q, D60E, L63P, A71V, V77I, V82A, T91A
Reverse transcriptase:	

III. Phenotype prediction

Drug	Resistance Factor RF (*)	z-score	Scored Positions (**)
ZDV	0	0	
ddI	0	0	
d4T	0	0	
3TC	0	0	
ABC	0	0	
TDF	0	0	
NVP	0	0	
EFV	0	0	
ETR (***)	Susceptible		
RPV (***)	Susceptible		
SQV	64.367	11.964	48V 73G 90L 84I 11V 74T 88N 53F 95C 26T 1P 80T 34E 47I 97L
IDV	26.767	8.357	88N 46M 29D 1P 48V 73G 21E 65E 84I 11V 10I 85I 30D 54S 90L
NFV	21.393	6.648	88N 30D 46M 97L 20K 73G 68G 90L 36M 31T 75V 74T 84I 5L 2Q
APV	13.582	7.902	33F 76L 50I 84I 46M 32V 85I 48V 22A 63P 1P 47I 13I 97L 45K
LPV	31.534	10.116	46M 84I 50I 33F 48V 76L 22A 7Q 63P 24L 20K 25D 82A 47I 92Q
TPV	1.58	0.967	69H 82A 84I 47I 72I 15I 71V 20K 90L 74T 46M 88N 19L 13I 39P
DRV	6.515	4.845	47I 33F 84I 76L 74T 43K 73G 46M 36M 65E 82A 93I 15I 7Q 8R
ATV	61.973	12.129	73G 84I 82A 4T 33F 48V 88N 90L 7Q 46M 36M 20K 24L 76L 45K

(*) based on LIBSVM, Copyright (c) 2000, Chih-Chung Chang and Chih-Jen Lin

(**) Positions are ordered according to their impact on the phenotype prediction. Differences with respect to HXB2 are underlined. Positions shown in red and in green contribute to an increase or decrease in resistance, repectively. At most 15 positions are shown for each drug.

(***) Resistance predictions and scored mutations for ETR and RPV were performed with rules-based drug-resistance interpretation models by HIV-GRADE (http://www.hiv-grade.de)

geno2pheno®

phenotype prediction from genotype (version 3.4)

IV. Interpretation

Patient:	Birth date:	Sampling date:
Current therapy:		Viral load:

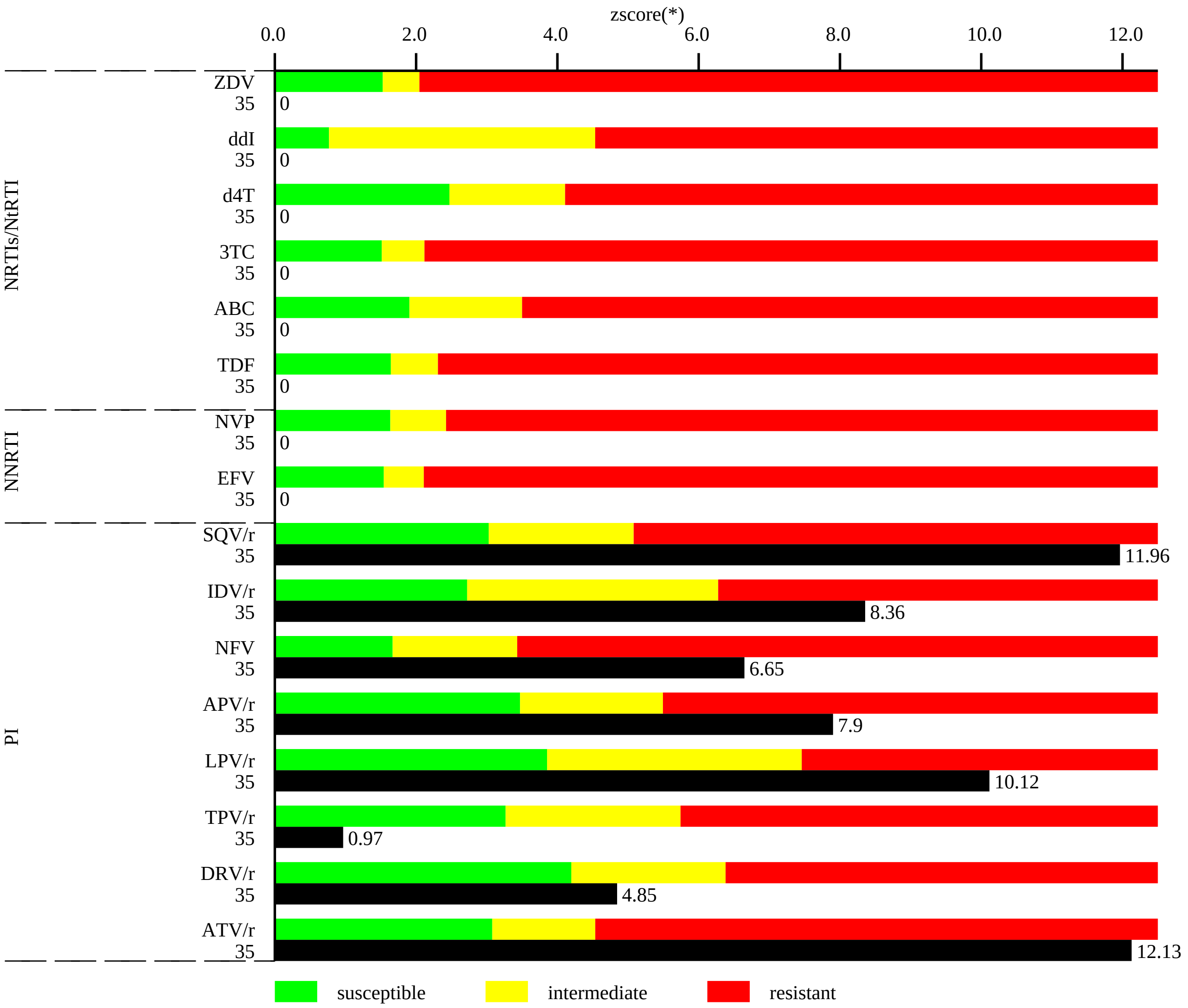

(*)number of standard deviations above mean of drug naive patients. Negative z-scores may indicate hypersusceptibility.

NRTIs/NtRTI:

NNRTIs:

PIs:

Previous genotypes:

date

signature
