# Supplementary material for: A Computational Approach for the Prediction of HIV Resistance Based on Amino Acid and Nucleotide Descriptors
Source: Molecules. 2018 Oct 24;23(11):2751. doi: 10.3390/molecules23112751 (PMC6278491; doi:10.3390/molecules23112751)
Supplement: Supplementary file 1 [file molecules-23-02751-s001.zip › Geno2Pheno-test-set-Predictions-PDF/geno2pheno-results/37.pdf]

geno2pheno®

phenotype prediction from genotype (version 3.4)

I. General information

Patient:	Study Id:
Birth date:	Viral load:
Sample received:	Sample collected:
Sample ID: 37	Predicted subtype: B (100%)
Sample type:	Report date: October 6, 2018
Physician:	Reported by:

II. Substitutions

(relative to the reference strain HXB2)

Protease:	V3I, L10I, I13V, K20R, L33F, E35D, M36I, S37E, K43T, M46I, I54V, R57K, D60E, Q61E, I62V, L63P, I64I/V, G68E, A71T, I72T, G73A/T, V82F, L90M, I93L
Reverse transcriptase:	

III. Phenotype prediction

Drug	Resistance Factor RF (*)	z-score	Scored Positions (**)
ZDV	0	0	
ddI	0	0	
d4T	0	0	
3TC	0	0	
ABC	0	0	
TDF	0	0	
NVP	0	0	
EFV	0	0	
ETR (***)	Susceptible		
RPV (***)	Susceptible		
SQV	38.405	10.463	48G 84I 11V 74T 88N 82F 90M 53F 95C 26T 1P 73T 80T 34E 47I
IDV	278.271	14.691	88N 82F 90M 29D 1P 71T 21E 65E 84I 46I 73T 11V 10I 85I 30D
NFV	241.62	12.357	88N 30D 90M 97L 82F 46I 31T 75V 74T 84I 5L 2Q 1P 29D 48G
APV	107.509	14.449	33F 76L 50I 84I 82F 32V 46I 85I 22A 43T 63P 1P 47I 90M 97L
LPV	132.417	14.27	84I 50I 46I 54V 33F 76L 82F 22A 7Q 63P 24L 25D 47I 92Q 2Q
TPV	10.361	5.044	69H 46I 48G 84I 90M 47I 15I 91T 74T 88N 58Q 19L 39P 89L 41R
DRV	21.84	8.101	47I 33F 84I 76L 74T 65E 43T 93L 48G 72T 15I 7Q 62V 8R 16G
ATV	109.959	13.903	48G 84I 90M 73T 71T 4T 33F 88N 7Q 24L 76L 45K 32V 93L 46I

(*) based on LIBSVM, Copyright (c) 2000, Chih-Chung Chang and Chih-Jen Lin

(**) Positions are ordered according to their impact on the phenotype prediction. Differences with respect to HXB2 are underlined. Positions shown in red and in green contribute to an increase or decrease in resistance, repectively. At most 15 positions are shown for each drug.

(***) Resistance predictions and scored mutations for ETR and RPV were performed with rules-based drug-resistance interpretation models by HIV-GRADE (http://www.hiv-grade.de)

geno2pheno®

phenotype prediction from genotype (version 3.4)

IV. Interpretation

Patient:	Birth date:	Sampling date:
Current therapy:		Viral load:

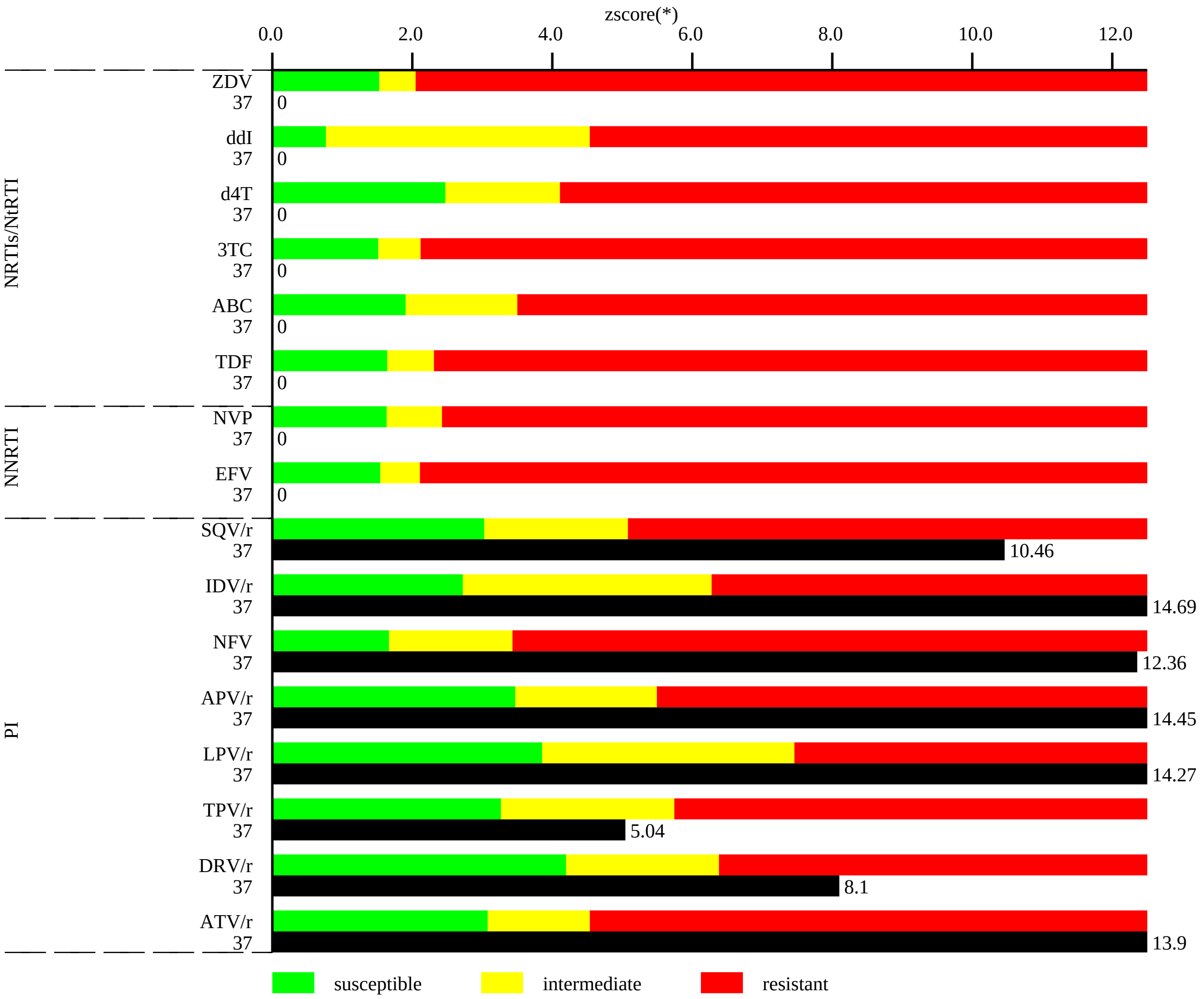

(*)number of standard deviations above mean of drug naive patients. Negative z-scores may indicate hypersusceptibility.

NRTIs/NtRTI:

NNRTIs:

PIs:

Previous genotypes:

date

signature
