# Supplementary material for: A Computational Approach for the Prediction of HIV Resistance Based on Amino Acid and Nucleotide Descriptors
Source: Molecules. 2018 Oct 24;23(11):2751. doi: 10.3390/molecules23112751 (PMC6278491; doi:10.3390/molecules23112751)
Supplement: Supplementary file 1 [file molecules-23-02751-s001.zip › Geno2Pheno-test-set-Predictions-PDF/geno2pheno-results/39.pdf]

geno2pheno®

phenotype prediction from genotype (version 3.4)

I. General information

Patient:		Study Id:	
Birth date:		Viral load:	
Sample received:		Sample collected:	
Sample ID:	39	Predicted subtype:	B (100%)
Sample type:		Report date:	October 6, 2018
Physician:		Reported by:	

II. Substitutions
(relative to the reference strain HXB2)

Protease:	V3I, L10I, T12P, I15V, K20R, V32I/V, M36I, S37D, M46L, I54L, L63P, A71V, T74S/T, P79S, V82A/V, I84V, I85V, L90M, I93L
Reverse transcriptase:	

III. Phenotype prediction

Drug	Resistance Factor RF (*)	z-score	Scored Positions (**)
ZDV	0	0	
ddI	0	0	
d4T	0	0	
3TC	0	0	
ABC	0	0	
TDF	0	0	
NVP	0	0	
EFV	0	0	
ETR (***)	Susceptible		
RPV (***)	Susceptible		
SQV	113.167	13.605	48G 84V 73G 11V 88N 90M 53F 95C 26T 1P 80T 34E 54L 47I 97L
IDV	401.033	15.679	54L 88N 90M 29D 1P 84V 73G 21E 65E 32I 11V 10I 30D 78G 98N
NFV	202.613	11.942	88N 30D 90M 84V 97L 73G 68G 31T 75V 54L 5L 2Q 1P 29D 48G
APV	198.8	16.394	76L 50I 84V 32I 54L 37D 22A 63P 1P 47I 13I 90M 97L 45K 10I
LPV	182.575	15.2	50I 84V 76L 54L 22A 7Q 63P 24L 25D 82A 74S 37D 47I 92Q 2Q
TPV	13.891	5.68	69H 48G 84V 33L 90M 47I 72I 91T 71V 15V 54L 82V 74S 88N 58Q
DRV	19.195	7.753	47I 84V 33L 76L 43K 73G 74S 37D 65E 93L 48G 32I 85V 7Q 8R
ATV	371.154	17.667	48G 73G 84V 90M 82A 4T 46L 88N 7Q 24L 76L 45K 32I 54L 93L

(*) based on LIBSVM, Copyright (c) 2000, Chih-Chung Chang and Chih-Jen Lin

(**) Positions are ordered according to their impact on the phenotype prediction. Differences with respect to HXB2 are underlined. Positions shown in red and in green contribute to an increase or decrease in resistance, repectively. At most 15 positions are shown for each drug.

(***) Resistance predictions and scored mutations for ETR and RPV were performed with rules-based drug-resistance interpretation models by HIV-GRADE (http://www.hiv-grade.de)

geno2pheno®

phenotype prediction from genotype (version 3.4)

IV. Interpretation

Patient:	Birth date:	Sampling date:
Current therapy:		Viral load:

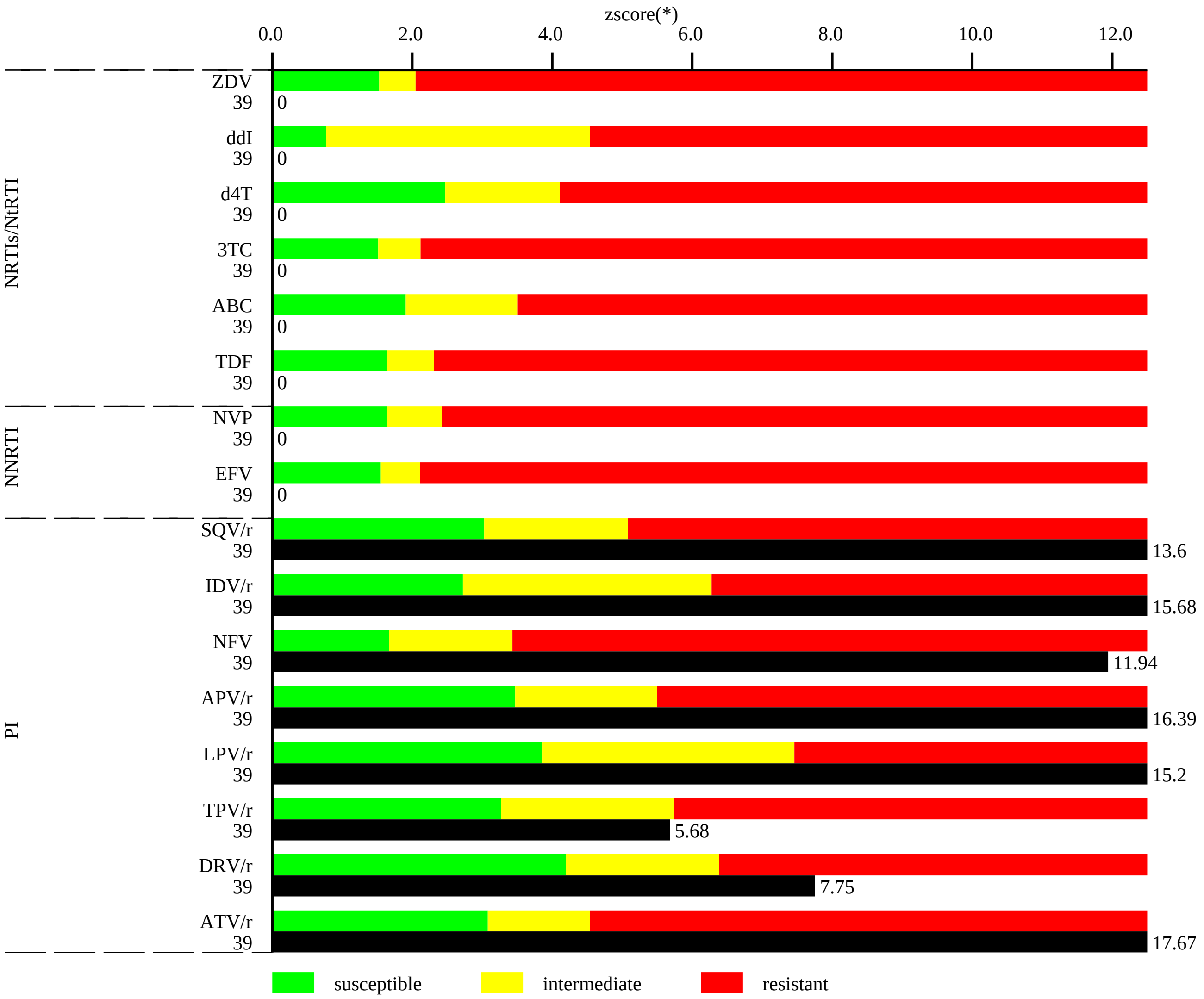

(*)number of standard deviations above mean of drug naive patients. Negative z-scores may indicate hypersusceptibility.

NRTIs/NtRTI:

NNRTIs:

PIs:

Previous genotypes:

date

signature
