# Supplementary material for: A Computational Approach for the Prediction of HIV Resistance Based on Amino Acid and Nucleotide Descriptors
Source: Molecules. 2018 Oct 24;23(11):2751. doi: 10.3390/molecules23112751 (PMC6278491; doi:10.3390/molecules23112751)
Supplement: Supplementary file 1 [file molecules-23-02751-s001.zip › Geno2Pheno-test-set-Predictions-PDF/geno2pheno-results/40.pdf]

geno2pheno®

phenotype prediction from genotype (version 3.4)

I. General information

Patient:		Study Id:	
Birth date:		Viral load:	
Sample received:		Sample collected:	
Sample ID:	40	Predicted subtype:	B (100%)
Sample type:		Report date:	October 6, 2018
Physician:		Reported by:	

II. Substitutions

(relative to the reference strain HXB2)

Protease:	V3I, K20V, S37N, F53L, L63P, A71V, G73S, V77I, I85I/V, L90M
Reverse transcriptase:	

III. Phenotype prediction

Drug	Resistance Factor RF (*)	z-score	Scored Positions (**)
ZDV	0	0	
ddI	0	0	
d4T	0	0	
3TC	0	0	
ABC	0	0	
TDF	0	0	
NVP	0	0	
EFV	0	0	
ETR (***)	Susceptible		
RPV (***)	Susceptible		
SQV	30.386	9.781	48G 84I 54I 11V 74T 88N 90M 95C 53L 26T 1P 80T 34E 47I 97L
IDV	10.458	5.815	54I 82V 88N 46M 90M 29D 1P 21E 65E 84I 20V 11V 30D 73S 78G
NFV	11.009	5.083	88N 54I 30D 90M 46M 82V 97L 68G 36M 31T 75V 74T 84I 5L 2Q
APV	2.798	2.903	54I 76L 50I 84I 46M 32V 22A 63P 1P 47I 13I 82V 90M 97L 45K
LPV	3.439	3.702	54I 82V 46M 84I 50I 76L 10L 22A 7Q 63P 24L 25D 47I 92Q 2Q
TPV	2.17	1.655	69H 48G 84I 33L 90M 54I 47I 72I 15I 91T 71V 73S 82V 74T 46M
DRV	2.564	2.336	47I 84I 54I 33L 76L 74T 43K 46M 36M 65E 48G 93I 10L 85V 15I
ATV	17.373	8.194	54I 48G 84I 82V 90M 4T 88N 7Q 73S 46M 36M 24L 76L 45K 32V

(*) based on LIBSVM, Copyright (c) 2000, Chih-Chung Chang and Chih-Jen Lin

(**) Positions are ordered according to their impact on the phenotype prediction. Differences with respect to HXB2 are underlined. Positions shown in red and in green contribute to an increase or decrease in resistance, repectively. At most 15 positions are shown for each drug.

(***) Resistance predictions and scored mutations for ETR and RPV were performed with rules-based drug-resistance interpretation models by HIV-GRADE (http://www.hiv-grade.de)

Contact: Info@geno2pheno.org
www.geno2pheno.org

geno2pheno®

phenotype prediction from genotype (version 3.4)

IV. Interpretation

Patient:	Birth date:	Sampling date:
Current therapy:		Viral load:

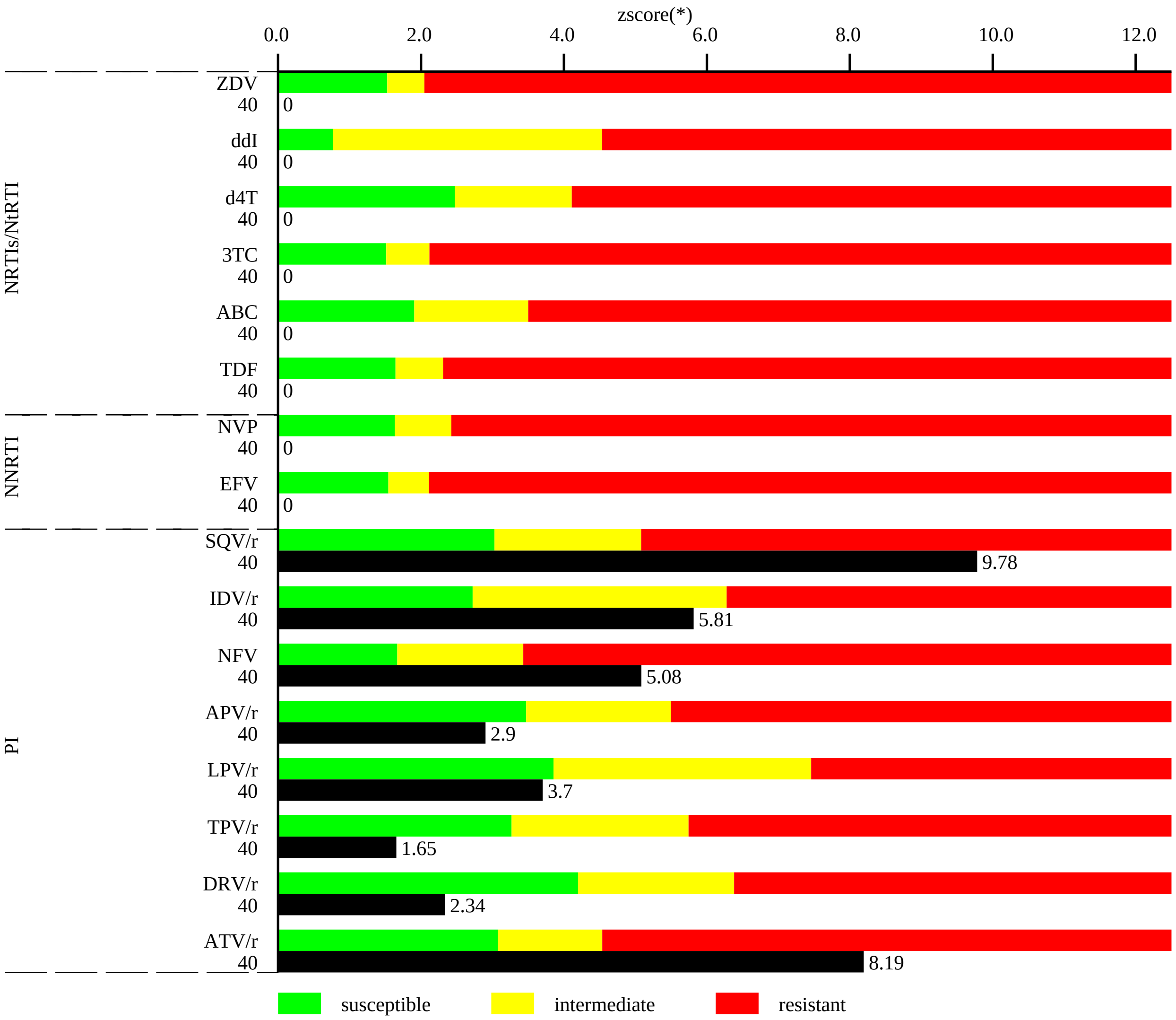

(*)number of standard deviations above mean of drug naive patients. Negative z-scores may indicate hypersusceptibility.

NRTIs/NtRTI:

NNRTIs:

PIs:

Previous genotypes:

date

signature
