# Supplementary material for: A Computational Approach for the Prediction of HIV Resistance Based on Amino Acid and Nucleotide Descriptors
Source: Molecules. 2018 Oct 24;23(11):2751. doi: 10.3390/molecules23112751 (PMC6278491; doi:10.3390/molecules23112751)
Supplement: Supplementary file 1 [file molecules-23-02751-s001.zip › Geno2Pheno-test-set-Predictions-PDF/geno2pheno-results/41.pdf]

geno2pheno®

phenotype prediction from genotype (version 3.4)

I. General information

Patient:	Study Id:
Birth date:	Viral load:
Sample received:	Sample collected:
Sample ID: 41	Predicted subtype: B (100%)
Sample type:	Report date: October 6, 2018
Physician:	Reported by:

II. Substitutions

(relative to the reference strain HXB2)

Protease:	V3I, I15V, K20R, V32I, M36I, S37N, M46I, I47I/V, F53L, I54V, L63P, A71I, V82A, L90M, I93L
Reverse transcriptase:	

III. Phenotype prediction

Drug	Resistance Factor RF (*)	z-score	Scored Positions (**)
ZDV	0	0	
ddI	0	0	
d4T	0	0	
3TC	0	0	
ABC	0	0	
TDF	0	0	
NVP	0	0	
EFV	0	0	
ETR (***)	Susceptible		
RPV (***)	Susceptible		
SQV	49.335	11.191	48G 73G 84I 11V 74T 88N 90M 95C 53L 26T 1P 80T 34E 47I 97L
IDV	160.612	13.204	88N 90M 29D 1P 73G 21E 65E 84I 32I 46I 11V 85I 30D 78G 98N
NFV	113.362	10.574	88N 30D 90M 97L 73G 68G 46I 31T 75V 74T 84I 5L 2Q 1P 29D
APV	44.229	11.638	76L 50I 84I 32I 46I 85I 47V 22A 63P 1P 13I 90M 97L 45K 21E
LPV	111.761	13.779	84I 50I 46I 54V 76L 10L 22A 7Q 63P 24L 47V 25D 82A 92Q 2Q
TPV	3.613	2.76	69H 82A 46I 48G 84I 33L 90M 47V 72I 91T 15V 74T 88N 58Q 19L
DRV	4.122	3.614	47V 84I 33L 76L 74T 43K 73G 65E 93L 48G 82A 32I 10L 7Q 8R
ATV	111.108	13.935	48G 73G 84I 90M 82A 4T 88N 7Q 24L 76L 45K 32I 93L 46I 41R

(*) based on LIBSVM, Copyright (c) 2000, Chih-Chung Chang and Chih-Jen Lin

(**) Positions are ordered according to their impact on the phenotype prediction. Differences with respect to HXB2 are underlined. Positions shown in red and in green contribute to an increase or decrease in resistance, repectively. At most 15 positions are shown for each drug.

(***) Resistance predictions and scored mutations for ETR and RPV were performed with rules-based drug-resistance interpretation models by HIV-GRADE (http://www.hiv-grade.de)

geno2pheno®

phenotype prediction from genotype (version 3.4)

IV. Interpretation

Patient:	Birth date:	Sampling date:
Current therapy:		Viral load:

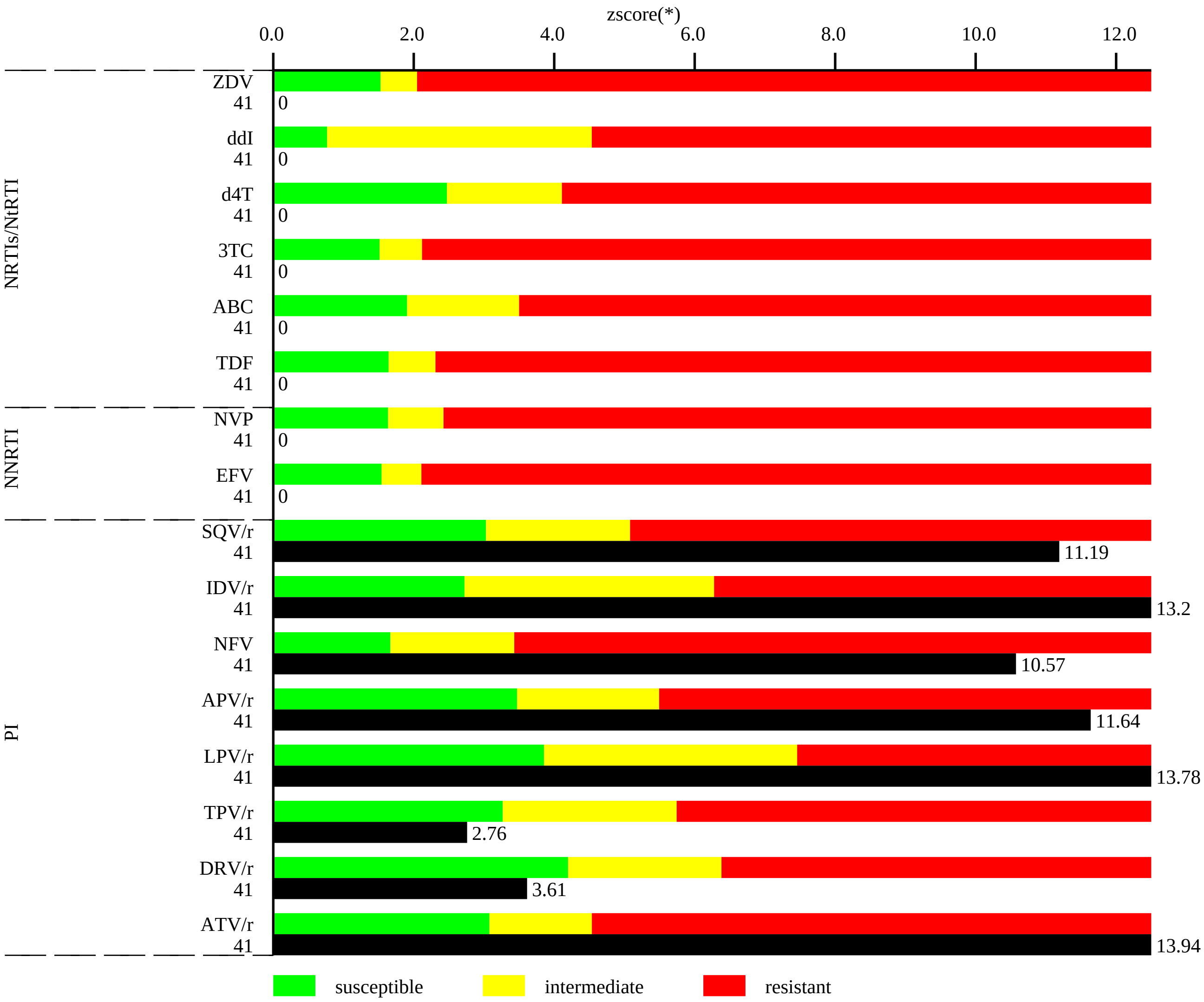

(*)number of standard deviations above mean of drug naive patients. Negative z-scores may indicate hypersusceptibility.

NRTIs/NtRTI:

NNRTIs:

PIs:

Previous genotypes:

date

signature
