# Supplementary material for: A Computational Approach for the Prediction of HIV Resistance Based on Amino Acid and Nucleotide Descriptors
Source: Molecules. 2018 Oct 24;23(11):2751. doi: 10.3390/molecules23112751 (PMC6278491; doi:10.3390/molecules23112751)
Supplement: Supplementary file 1 [file molecules-23-02751-s001.zip › Geno2Pheno-test-set-Predictions-PDF/geno2pheno-results/44.pdf]

geno2pheno®

phenotype prediction from genotype (version 3.4)

I. General information

Patient:	Study Id:
Birth date:	Viral load:
Sample received:	Sample collected:
Sample ID: 44	Predicted subtype: B (100%)
Sample type:	Report date: October 6, 2018
Physician:	Reported by:

II. Substitutions

(relative to the reference strain HXB2)

Protease:	V3I, L10F, V11I, I13V, G16A, L19L/V, L33F, E34Q, S37N, K43I, M46L, G51A, I54M, L63P, I64M, C67Y, A71V, I72M, G73A, I84V, L90M
Reverse transcriptase:	

III. Phenotype prediction

Drug	Resistance Factor RF (*)	z-score	Scored Positions (**)
ZDV	0	0	
ddI	0	0	
d4T	0	0	
3TC	0	0	
ABC	0	0	
TDF	0	0	
NVP	0	0	
EFV	0	0	
ETR (***)	Susceptible		
RPV (***)	Susceptible		
SQV	54.816	11.497	48G 84V 74T 88N 90M 53F 95C 26T 11I 1P 80T 19V 47I 97L 24L
IDV	52.081	10.158	82V 88N 90M 29D 1P 84V 21E 65E 67Y 85I 30D 78G 98N 76L 48G
NFV	58.268	9.007	88N 30D 90M 84V 82V 97L 20K 68G 36M 31T 75V 74T 5L 2Q 1P
APV	136.071	15.195	33F 76L 50I 84V 54M 32V 85I 10F 22A 63P 1P 47I 82V 90M 97L
LPV	74.272	12.596	82V 50I 84V 33F 76L 22A 10F 7Q 63P 24L 20K 25D 54M 47I 92Q
TPV	19.779	6.446	69H 48G 84V 90M 47I 15I 91T 71V 20K 82V 74T 88N 58Q 39P 89L
DRV	91.309	11.95	47I 33F 84V 76L 74T 54M 36M 65E 10F 48G 93I 15I 7Q 8R 72M
ATV	90.864	13.313	48G 84V 82V 90M 4T 33F 46L 88N 7Q 36M 20K 24L 76L 54M 45K

(*) based on LIBSVM, Copyright (c) 2000, Chih-Chung Chang and Chih-Jen Lin

(**) Positions are ordered according to their impact on the phenotype prediction. Differences with respect to HXB2 are underlined. Positions shown in red and in green contribute to an increase or decrease in resistance, repectively. At most 15 positions are shown for each drug.

(***) Resistance predictions and scored mutations for ETR and RPV were performed with rules-based drug-resistance interpretation models by HIV-GRADE (http://www.hiv-grade.de)

geno2pheno®

phenotype prediction from genotype (version 3.4)

IV. Interpretation

Patient:	Birth date:	Sampling date:
Current therapy:		Viral load:

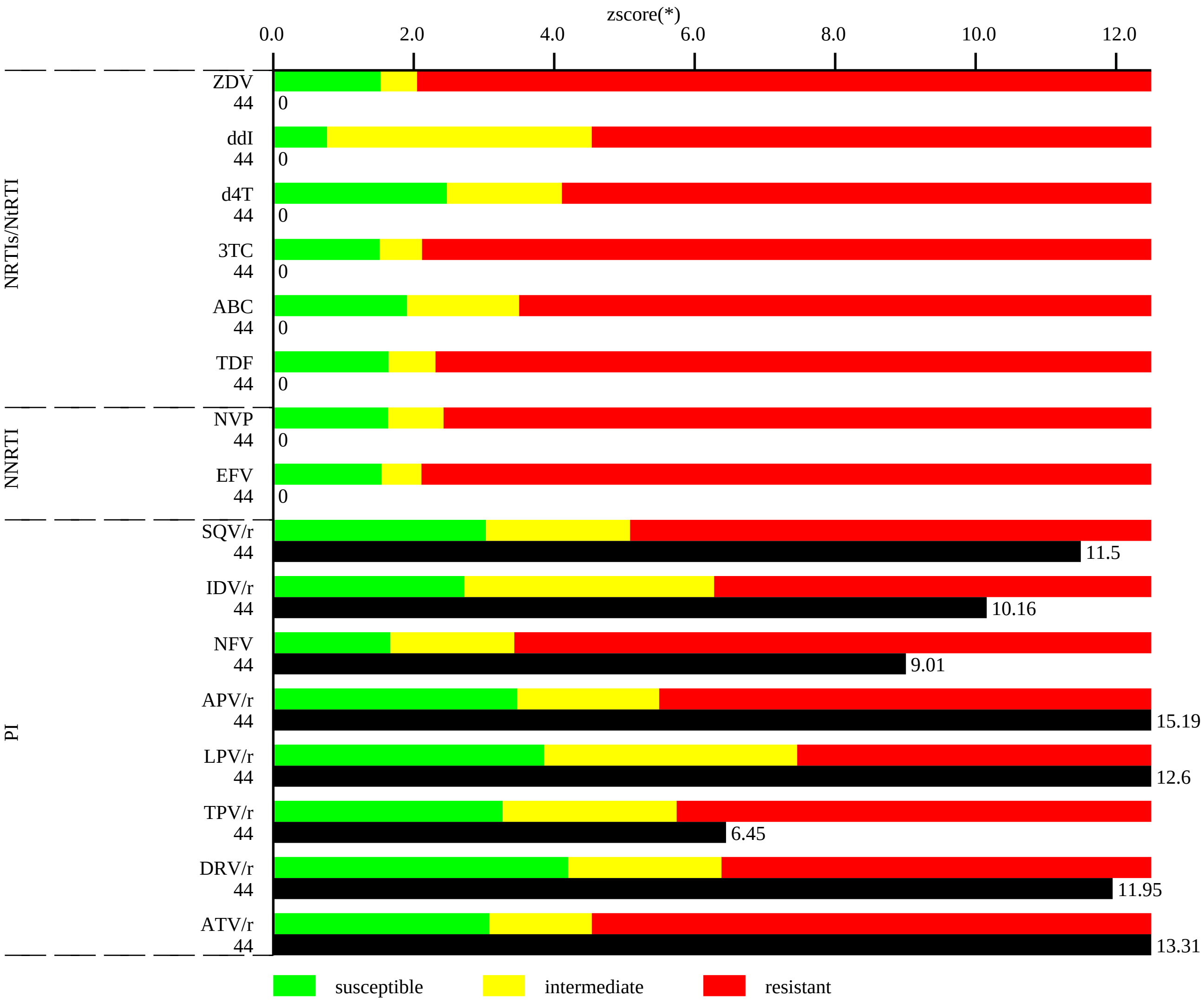

(*)number of standard deviations above mean of drug naive patients. Negative z-scores may indicate hypersusceptibility.

NRTIs/NtRTI:

NNRTIs:

PIs:

Previous genotypes:

date

signature
