# Supplementary material for: A Computational Approach for the Prediction of HIV Resistance Based on Amino Acid and Nucleotide Descriptors
Source: Molecules. 2018 Oct 24;23(11):2751. doi: 10.3390/molecules23112751 (PMC6278491; doi:10.3390/molecules23112751)
Supplement: Supplementary file 1 [file molecules-23-02751-s001.zip › Geno2Pheno-test-set-Predictions-PDF/geno2pheno-results/45.pdf]

geno2pheno®

phenotype prediction from genotype (version 3.4)

I. General information

Patient:	Study Id:
Birth date:	Viral load:
Sample received:	Sample collected:
Sample ID: 45	Predicted subtype: B (100%)
Sample type:	Report date: October 6, 2018
Physician:	Reported by:

II. Substitutions
(relative to the reference strain HXB2)

Protease:	V3I, L10F, V11I, I13I/V, K20R, V32I, L33F, E35D, M36I, S37Q, M46I, I47V, I54L, R57K, Q58E, D60E, I62V, L63P, A71V, G73S, V77T, I84V, L89V, L90M, I93L
Reverse transcriptase:	

III. Phenotype prediction

Drug	Resistance Factor RF (*)	z-score	Scored Positions (**)
ZDV	0	0	
ddI	0	0	
d4T	0	0	
3TC	0	0	
ABC	0	0	
TDF	0	0	
NVP	0	0	
EFV	0	0	
ETR (***)	Susceptible		
RPV (***)	Susceptible		
SQV	64.598	11.975	48G 84V 74T 88N 90M 47V 53F 95C 26T 11I 1P 80T 34E 54L 97L
IDV	112.897	12.25	82V 54L 88N 90M 29D 1P 84V 21E 65E 32I 46I 85I 30D 73S 78G
NFV	315.989	12.988	88N 30D 90M 84V 82V 97L 68G 46I 31T 75V 74T 54L 5L 2Q 1P
APV	1003.993	21.519	33F 76L 50I 84V 32I 89V 46I 54L 85I 47V 10F 22A 63P 1P 82V
LPV	212.611	15.641	82V 50I 46I 84V 33F 76L 54L 89V 22A 10F 7Q 63P 24L 47V 25D
TPV	10.052	4.979	69H 46I 48G 84V 90M 47V 72I 15I 91T 71V 54L 73S 82V 74T 88N
DRV	127.737	12.854	47V 33F 84V 76L 74T 43K 65E 10F 93L 48G 32I 15I 7Q 62V 8R
ATV	311.536	17.125	48G 84V 82V 90M 4T 33F 88N 7Q 73S 24L 76L 45K 32I 54L 93L

(*) based on LIBSVM, Copyright (c) 2000, Chih-Chung Chang and Chih-Jen Lin

(**) Positions are ordered according to their impact on the phenotype prediction. Differences with respect to HXB2 are underlined. Positions shown in red and in green contribute to an increase or decrease in resistance, repectively. At most 15 positions are shown for each drug.

(***) Resistance predictions and scored mutations for ETR and RPV were performed with rules-based drug-resistance interpretation models by HIV-GRADE (http://www.hiv-grade.de)

geno2pheno®

phenotype prediction from genotype (version 3.4)

IV. Interpretation

Patient:	Birth date:	Sampling date:
Current therapy:		Viral load:

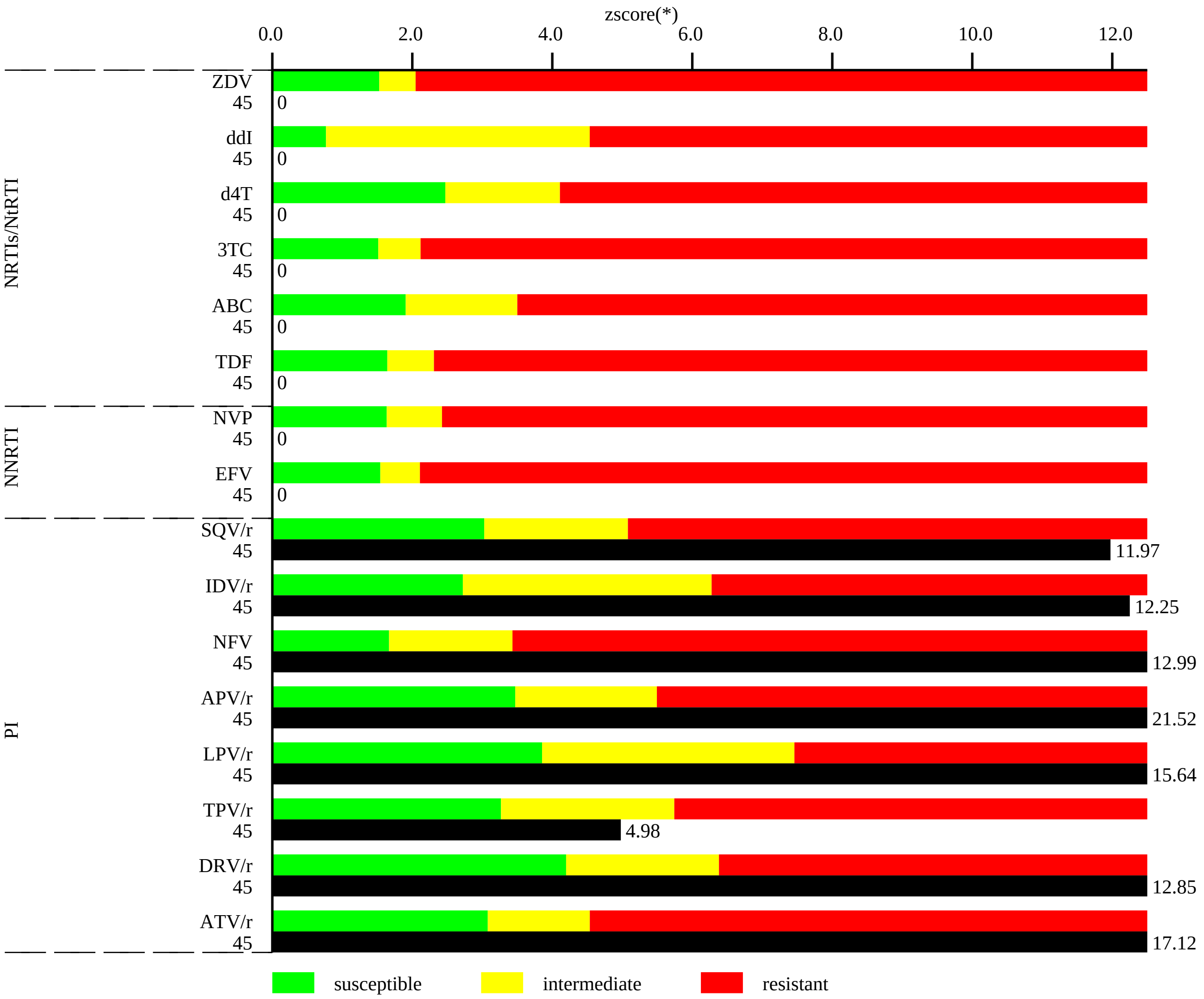

(*)number of standard deviations above mean of drug naive patients. Negative z-scores may indicate hypersusceptibility.

NRTIs/NtRTI:

NNRTIs:

PIs:

Previous genotypes:

date

signature
