# Supplementary material for: A Computational Approach for the Prediction of HIV Resistance Based on Amino Acid and Nucleotide Descriptors
Source: Molecules. 2018 Oct 24;23(11):2751. doi: 10.3390/molecules23112751 (PMC6278491; doi:10.3390/molecules23112751)
Supplement: Supplementary file 1 [file molecules-23-02751-s001.zip › Geno2Pheno-test-set-Predictions-PDF/geno2pheno-results/46.pdf]

geno2pheno®

phenotype prediction from genotype (version 3.4)

I. General information

Patient:	Study Id:
Birth date:	Viral load:
Sample received:	Sample collected:
Sample ID: 46	Predicted subtype: B (100%)
Sample type:	Report date: October 6, 2018
Physician:	Reported by:

II. Substitutions

(relative to the reference strain HXB2)

Protease:	V3I, L10F, I15V, S37N, R41K, M46I, K55R, I62I/V, L63P, A71T, G73G/S, V82T, I84V, I85V, L89L/M
Reverse transcriptase:	

III. Phenotype prediction

Drug	Resistance Factor RF (*)	z-score	Scored Positions (**)
ZDV	0	0	
ddI	0	0	
d4T	0	0	
3TC	0	0	
ABC	0	0	
TDF	0	0	
NVP	0	0	
EFV	0	0	
ETR (***)	Susceptible		
RPV (***)	Susceptible		
SQV	22.18	8.866	48G 84V 90L 54I 11V 74T 88N 53F 95C 26T 1P 80T 34E 47I 97L
IDV	40.471	9.475	54I 88N 29D 1P 84V 71T 21E 65E 46I 11V 30D 90L 73S 78G 98N
NFV	13.584	5.578	88N 54I 30D 84V 97L 20K 68G 90L 36M 46I 31T 75V 74T 5L 2Q
APV	11.546	7.389	54I 76L 50I 84V 32V 46I 82T 10F 22A 63P 1P 47I 13I 97L 45K
LPV	30.105	9.982	54I 50I 46I 84V 76L 22A 10F 7Q 63P 24L 20K 25D 47I 92Q 90L
TPV	2.978	2.341	69H 46I 48G 84V 33L 54I 82T 47I 72I 91T 20K 15V 90L 74T 88N
DRV	10.668	6.173	47I 84V 54I 33L 76L 74T 43K 36M 65E 10F 48G 93I 85V 7Q 62V
ATV	11.989	7.047	54I 48G 84V 71T 4T 88N 90L 7Q 73S 36M 20K 24L 76L 45K 32V

(*) based on LIBSVM, Copyright (c) 2000, Chih-Chung Chang and Chih-Jen Lin

(**) Positions are ordered according to their impact on the phenotype prediction. Differences with respect to HXB2 are underlined. Positions shown in red and in green contribute to an increase or decrease in resistance, repectively. At most 15 positions are shown for each drug.

(***) Resistance predictions and scored mutations for ETR and RPV were performed with rules-based drug-resistance interpretation models by HIV-GRADE (http://www.hiv-grade.de)

geno2pheno®

phenotype prediction from genotype (version 3.4)

IV. Interpretation

Patient:	Birth date:	Sampling date:
Current therapy:		Viral load:

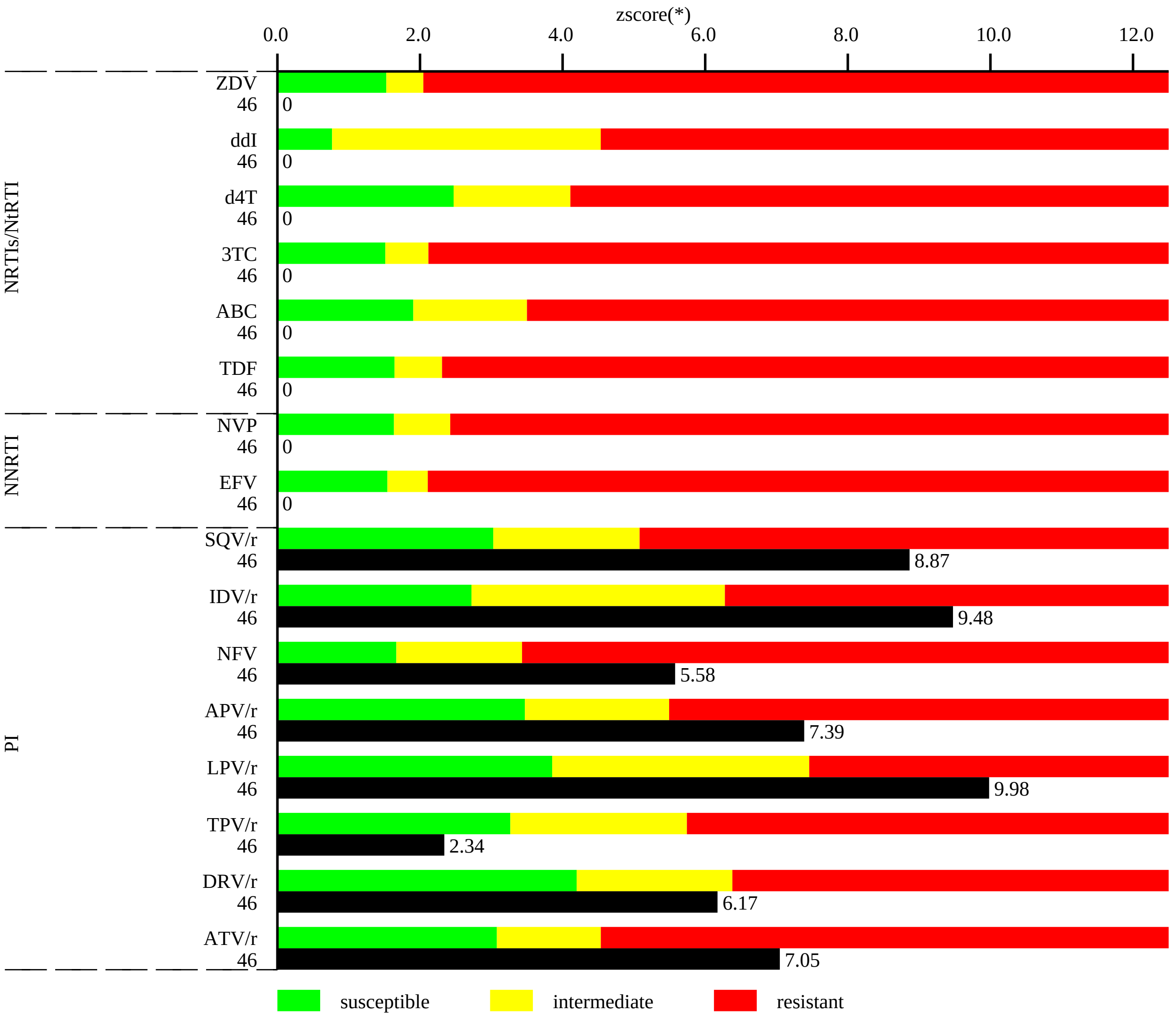

(*)number of standard deviations above mean of drug naive patients. Negative z-scores may indicate hypersusceptibility.

NRTIs/NtRTI:

NNRTIs:

PIs:

Previous genotypes:

date

signature
