# Supplementary material for: A Computational Approach for the Prediction of HIV Resistance Based on Amino Acid and Nucleotide Descriptors
Source: Molecules. 2018 Oct 24;23(11):2751. doi: 10.3390/molecules23112751 (PMC6278491; doi:10.3390/molecules23112751)
Supplement: Supplementary file 1 [file molecules-23-02751-s001.zip › Geno2Pheno-test-set-Predictions-PDF/geno2pheno-results/5.pdf]

geno2pheno®

phenotype prediction from genotype (version 3.4)

I. General information

Patient:		Study Id:	
Birth date:		Viral load:	
Sample received:		Sample collected:	
Sample ID:	5	Predicted subtype:	B (100%)
Sample type:		Report date:	October 6, 2018
Physician:		Reported by:	

II. Substitutions
(relative to the reference strain HXB2)

Protease:	G16E/G, S37N, R41K, K43K/R, K45K/R, I62V, L63P/T, I72I/T, V77I, V82I/V, I93L
Reverse transcriptase:	

III. Phenotype prediction

Drug	Resistance Factor RF (*)	z-score	Scored Positions (**)
ZDV	0	0	
ddI	0	0	
d4T	0	0	
3TC	0	0	
ABC	0	0	
TDF	0	0	
NVP	0	0	
EFV	0	0	
ETR (***)	Susceptible		
RPV (***)	Susceptible		
SQV	0.822	-0.716	48G 73G 90L 84I 54I 11V 74T 88N 53F 95C 26T 71A 80T 34E 47I
IDV	1.74	0.962	54I 82V 88N 46M 29D 73G 21E 65E 84I 11V 71A 85I 30D 90L 78G
NFV	1.695	0.677	88N 54I 30D 46M 97L 20K 73G 68G 90L 71A 36M 31T 75V 74T 84I
APV	1.39	0.689	54I 76L 50I 84I 46M 32V 85I 22A 63P 47I 13I 97L 21E 29D 65E
LPV	1.594	1.475	54I 46M 84I 50I 82I 76L 10L 22A 71A 7Q 63P 24L 16E 20K 25D
TPV	1.308	0.558	69H 48G 84I 33L 54I 47I 71A 15I 91T 20K 90L 74T 46M 88N 58Q
DRV	1.312	0.532	47I 84I 54I 33L 76L 74T 73G 46M 71A 36M 65E 93L 48G 72T
ATV	2.095	1.65	54I 48G 73G 84I 4T 71A 88N 90L 7Q 46M 36M 20K 24L 76L 32V

(*) based on LIBSVM, Copyright (c) 2000, Chih-Chung Chang and Chih-Jen Lin

(**) Positions are ordered according to their impact on the phenotype prediction. Differences with respect to HXB2 are underlined. Positions shown in red and in green contribute to an increase or decrease in resistance, repectively. At most 15 positions are shown for each drug.

(***) Resistance predictions and scored mutations for ETR and RPV were performed with rules-based drug-resistance interpretation models by HIV-GRADE (http://www.hiv-grade.de)

geno2pheno®

phenotype prediction from genotype (version 3.4)

IV. Interpretation

Patient:	Birth date:	Sampling date:
Current therapy:		Viral load:

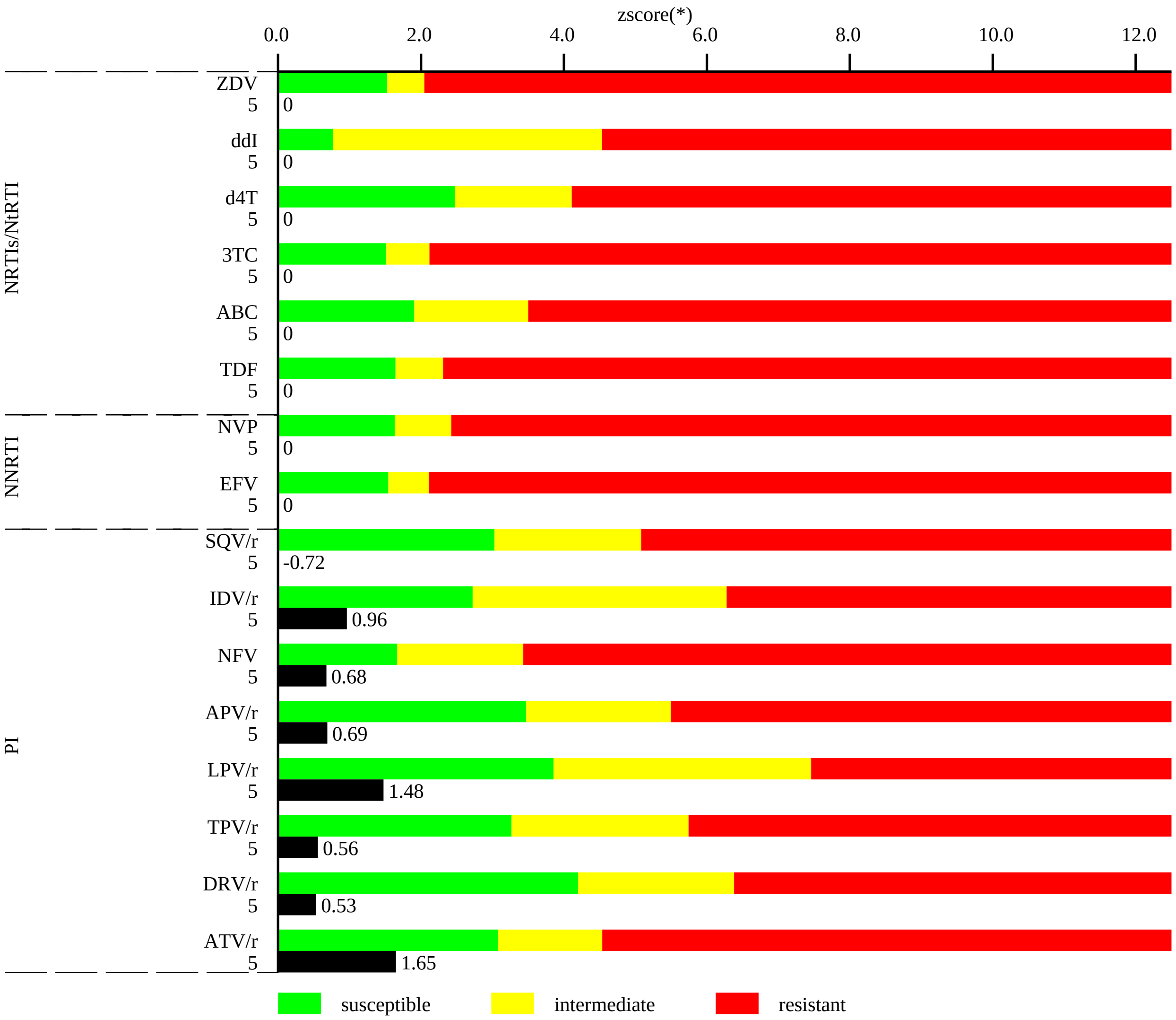

(*)number of standard deviations above mean of drug naive patients. Negative z-scores may indicate hypersusceptibility.

NRTIs/NtRTI:

NNRTIs:

PIs:

Previous genotypes:

date

signature
