# Supplementary material for: A Computational Approach for the Prediction of HIV Resistance Based on Amino Acid and Nucleotide Descriptors
Source: Molecules. 2018 Oct 24;23(11):2751. doi: 10.3390/molecules23112751 (PMC6278491; doi:10.3390/molecules23112751)
Supplement: Supplementary file 1 [file molecules-23-02751-s001.zip › Geno2Pheno-test-set-Predictions-PDF/geno2pheno-results/50.pdf]

geno2pheno®

phenotype prediction from genotype (version 3.4)

I. General information

Patient:	Study Id:
Birth date:	Viral load:
Sample received:	Sample collected:
Sample ID: 50	Predicted subtype: B (100%)
Sample type:	Report date: October 7, 2018
Physician:	Reported by:

II. Substitutions
(relative to the reference strain HXB2)

Protease:	V3I, T4A/T, L10F, I15I/V, K20K/T, E35D, S37N, M46I, I54V, L63P, I64V, A71V, I72L, G73T, V77I/V, I84V, I85V, L90M
Reverse transcriptase:	

III. Phenotype prediction

Drug	Resistance Factor RF (*)	z-score	Scored Positions (**)
ZDV	0	0	
ddI	0	0	
d4T	0	0	
3TC	0	0	
ABC	0	0	
TDF	0	0	
NVP	0	0	
EFV	0	0	
ETR (***)	Susceptible		
RPV (***)	Susceptible		
SQV	323.763	16.661	48G 84V 11V 74T 88N 90M 53F 95C 26T 1P 73T 80T 34E 72L 47I
IDV	396.539	15.649	82V 88N 90M 29D 1P 84V 21E 65E 46I 73T 11V 72L 30D 78G 98N
NFV	477.456	13.96	88N 30D 90M 84V 82V 97L 68G 36M 46I 31T 75V 74T 5L 2Q 1P
APV	89.663	13.875	76L 50I 84V 32V 46I 10F 22A 63P 1P 47I 13I 82V 90M 97L 45K
LPV	153.621	14.7	82V 50I 46I 54V 84V 76L 22A 10F 7Q 63P 24L 25D 47I 92Q 2Q
TPV	11.279	5.228	69H 46I 48G 84V 33L 90M 47I 91T 71V 15V 82V 74T 88N 58Q 19L
DRV	14.212	6.944	47I 84V 33L 76L 74T 43K 36M 65E 10F 48G 93I 85V 15I 7Q 8R
ATV	290.614	16.91	48G 84V 82V 90M 73T 72L 88N 7Q 36M 24L 76L 45K 32V 20T 46I

(*) based on LIBSVM, Copyright (c) 2000, Chih-Chung Chang and Chih-Jen Lin

(**) Positions are ordered according to their impact on the phenotype prediction. Differences with respect to HXB2 are underlined. Positions shown in red and in green contribute to an increase or decrease in resistance, repectively. At most 15 positions are shown for each drug.

(***) Resistance predictions and scored mutations for ETR and RPV were performed with rules-based drug-resistance interpretation models by HIV-GRADE (http://www.hiv-grade.de)

geno2pheno®

phenotype prediction from genotype (version 3.4)

IV. Interpretation

Patient:	Birth date:	Sampling date:
Current therapy:		Viral load:

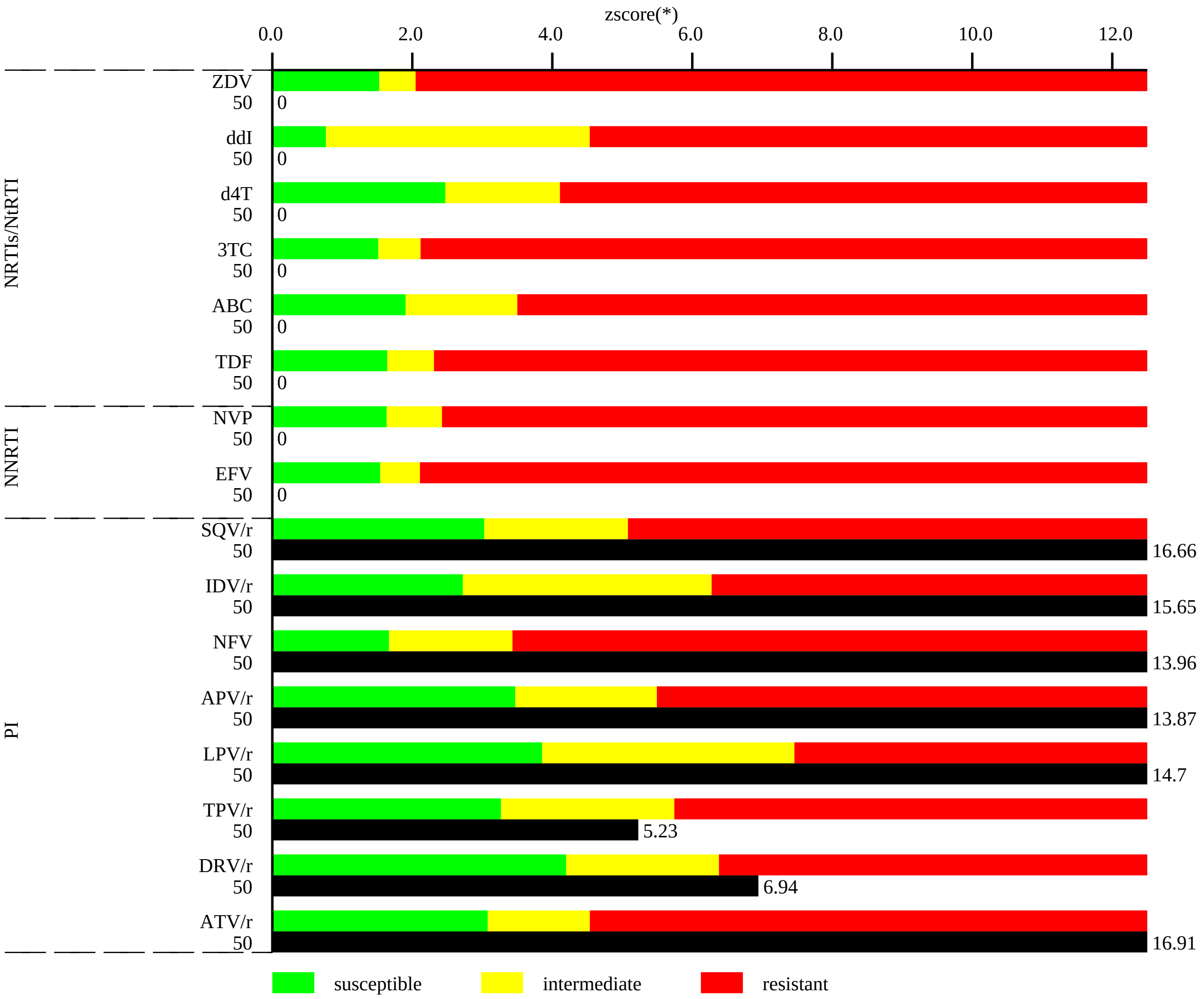

(*)number of standard deviations above mean of drug naive patients. Negative z-scores may indicate hypersusceptibility.

NRTIs/NtRTI:

NNRTIs:

PIs:

Previous genotypes:

date

signature
