# Supplementary material for: A Computational Approach for the Prediction of HIV Resistance Based on Amino Acid and Nucleotide Descriptors
Source: Molecules. 2018 Oct 24;23(11):2751. doi: 10.3390/molecules23112751 (PMC6278491; doi:10.3390/molecules23112751)
Supplement: Supplementary file 1 [file molecules-23-02751-s001.zip › Geno2Pheno-test-set-Predictions-PDF/geno2pheno-results/51.pdf]

geno2pheno®

phenotype prediction from genotype (version 3.4)

I. General information

Patient:	Study Id:
Birth date:	Viral load:
Sample received:	Sample collected:
Sample ID: 51	Predicted subtype: B (100%)
Sample type:	Report date: October 7, 2018
Physician:	Reported by:

II. Substitutions

(relative to the reference strain HXB2)

Protease:	V3I, L10I/L, V32I/V, L33M, S37N, R41K, K43K/R, M46I, I62V, L63P, C67C/F, A71I/T, I72V, G73C/G, T74P, V77I, L90M, I93L
Reverse transcriptase:	

III. Phenotype prediction

Drug	Resistance Factor RF (*)	z-score	Scored Positions (**)
ZDV	0	0	
ddI	0	0	
d4T	0	0	
3TC	0	0	
ABC	0	0	
TDF	0	0	
NVP	0	0	
EFV	0	0	
ETR (***)	Susceptible		
RPV (***)	Susceptible		
SQV	27.825	9.526	48G 84I 54I 11V 88N 90M 53F 95C 26T 1P 80T 34E 47I 97L 24L
IDV	52.725	10.191	54I 82V 88N 90M 29D 1P 21E 65E 84I 32I 46I 11V 10I 85I 30D
NFV	74.943	9.6	88N 54I 30D 90M 82V 97L 20K 68G 36M 46I 31T 75V 84I 5L 2Q
APV	14.277	8.06	54I 76L 50I 84I 32I 46I 85I 22A 63P 1P 47I 13I 82V 90M 97L
LPV	7.233	5.854	54I 82V 84I 50I 46I 76L 22A 7Q 63P 24L 20K 25D 47I 92Q 2Q
TPV	1.625	1.028	69H 46I 48G 84I 90M 54I 47I 15I 91T 20K 82V 88N 58Q 19L 13I
DRV	7.824	5.338	47I 84I 54I 76L 36M 65E 93L 48G 32I 15I 7Q 62V 8R 16G
ATV	11.473	6.911	54I 48G 84I 82V 90M 4T 88N 7Q 36M 20K 24L 76L 45K 32I 41K

(*) based on LIBSVM, Copyright (c) 2000, Chih-Chung Chang and Chih-Jen Lin

(**) Positions are ordered according to their impact on the phenotype prediction. Differences with respect to HXB2 are underlined. Positions shown in red and in green contribute to an increase or decrease in resistance, repectively. At most 15 positions are shown for each drug.

(***) Resistance predictions and scored mutations for ETR and RPV were performed with rules-based drug-resistance interpretation models by HIV-GRADE (http://www.hiv-grade.de)

geno2pheno®

phenotype prediction from genotype (version 3.4)

IV. Interpretation

Patient:	Birth date:	Sampling date:
Current therapy:		Viral load:

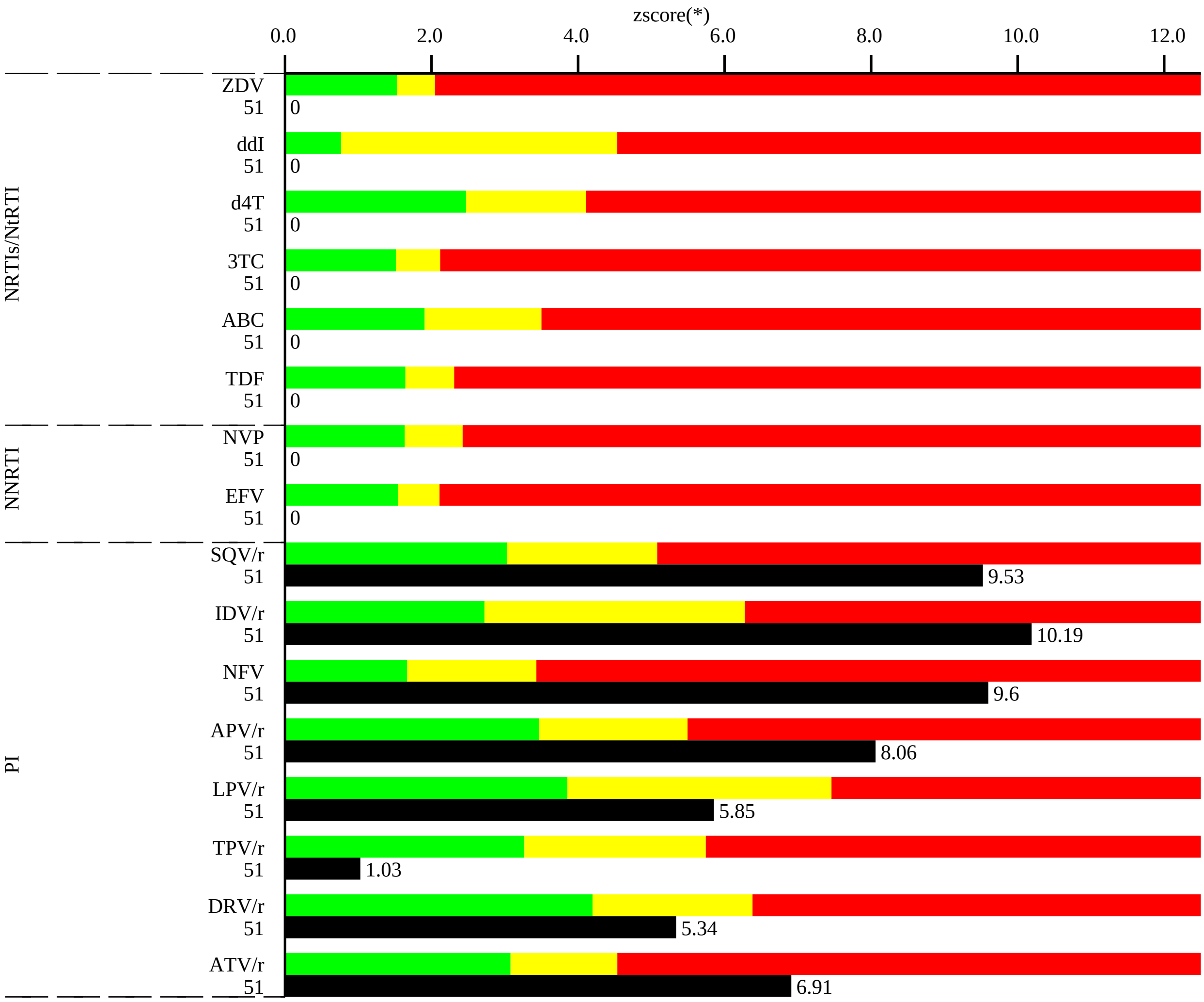

susceptible intermediate resistant

(*)number of standard deviations above mean of drug naive patients. Negative z-scores may indicate hypersusceptibility.

NRTIs/NtRTI:

NNRTIs:

PIs:

Previous genotypes:

date

signature
