# Supplementary material for: A Computational Approach for the Prediction of HIV Resistance Based on Amino Acid and Nucleotide Descriptors
Source: Molecules. 2018 Oct 24;23(11):2751. doi: 10.3390/molecules23112751 (PMC6278491; doi:10.3390/molecules23112751)
Supplement: Supplementary file 1 [file molecules-23-02751-s001.zip › Geno2Pheno-test-set-Predictions-PDF/geno2pheno-results/8.pdf]

geno2pheno®

phenotype prediction from genotype (version 3.4)

I. General information

Patient:	Study Id:
Birth date:	Viral load:
Sample received:	Sample collected:
Sample ID: 8	Predicted subtype: B (100%)
Sample type:	Report date: October 6, 2018
Physician:	Reported by:

II. Substitutions

(relative to the reference strain HXB2)

Protease:	L10I/V, I13V, K20R, L24F, L33F, E35D, M36I, S37N, K43Q, K45R, M46L/M, I54V, K55R, R57K, Q61N, I62V, L63P, A71I, G73S, V82A, I85L, L90M, I93L
Reverse transcriptase:	

III. Phenotype prediction

Drug	Resistance Factor RF (*)	z-score	Scored Positions (**)
ZDV	0	0	
ddI	0	0	
d4T	0	0	
3TC	0	0	
ABC	0	0	
TDF	0	0	
NVP	0	0	
EFV	0	0	
ETR (***)	Susceptible		
RPV (***)	Susceptible		
SQV	80.952	12.631	48G 84I 11V 74T 88N 90M 53F 95C 26T 80T 34E 47I 97L 73S 78G
IDV	391.344	15.613	88N 90M 29D 21E 65E 84I 11V 10I 30D 73S 78G 98N 76L 48G 24F
NFV	326.348	13.064	88N 30D 90M 97L 68G 31T 75V 74T 84I 5L 29D 55R 48G 71I 16G
APV	99.173	14.194	33F 76L 50I 84I 32V 22A 63P 47I 90M 97L 10I 46L 21E 29D 65E
LPV	152.672	14.682	84I 50I 54V 33F 76L 22A 7Q 63P 25D 82A 47I 92Q 48G 74T 61N
TPV	6.446	4.015	69H 82A 48G 84I 90M 47I 72I 15I 91T 73S 74T 46M 10V 88N 58Q
DRV	8.674	5.616	47I 33F 84I 76L 74T 65E 93L 48G 82A 15I 7Q 62V 8R 16G
ATV	208.135	15.877	48G 84I 90M 82A 4T 33F 46L 88N 7Q 73S 76L 32V 93L 53F 41R

(*) based on LIBSVM, Copyright (c) 2000, Chih-Chung Chang and Chih-Jen Lin

(**) Positions are ordered according to their impact on the phenotype prediction. Differences with respect to HXB2 are underlined. Positions shown in red and in green contribute to an increase or decrease in resistance, repectively. At most 15 positions are shown for each drug.

(***) Resistance predictions and scored mutations for ETR and RPV were performed with rules-based drug-resistance interpretation models by HIV-GRADE (http://www.hiv-grade.de)

geno2pheno®

phenotype prediction from genotype (version 3.4)

IV. Interpretation

Patient:	Birth date:	Sampling date:
Current therapy:		Viral load:

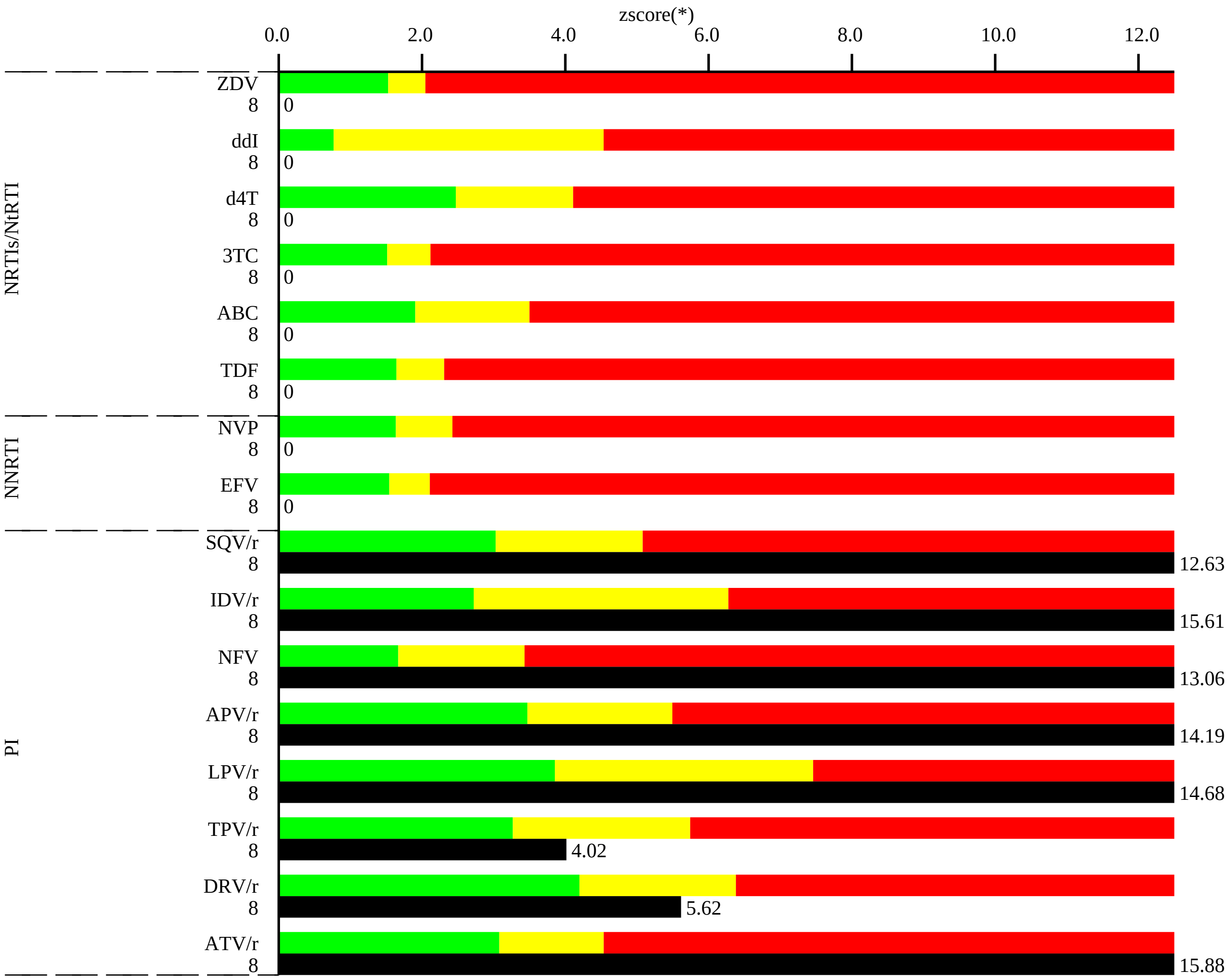

susceptible intermediate resistant

(*)number of standard deviations above mean of drug naive patients. Negative z-scores may indicate hypersusceptibility.

NRTIs/NtRTI:

NNRTIs:

PIs:

Previous genotypes:

date

signature
