# Supplementary material for: A Computational Approach for the Prediction of HIV Resistance Based on Amino Acid and Nucleotide Descriptors
Source: Molecules. 2018 Oct 24;23(11):2751. doi: 10.3390/molecules23112751 (PMC6278491; doi:10.3390/molecules23112751)
Supplement: Supplementary file 1 [file molecules-23-02751-s001.zip › Geno2Pheno-test-set-Predictions-PDF/geno2pheno-results/9.pdf]

geno2pheno®

phenotype prediction from genotype (version 3.4)

I. General information

Patient:	Study Id:
Birth date:	Viral load:
Sample received:	Sample collected:
Sample ID: 9	Predicted subtype: B (100%)
Sample type:	Report date: October 6, 2018
Physician:	Reported by:

II. Substitutions

(relative to the reference strain HXB2)

Protease:	L10F, I15I/V, K20R, V32I, L33F, E34Q, E35D, M36I, S37N/T, M46I, I47V, F53L, I54L, K55N, Q58E, L63L/P, I64L, T74K, I84V, L89M, Q92K
Reverse transcriptase:	

III. Phenotype prediction

Drug	Resistance Factor RF (*)	z-score	Scored Positions (**)
ZDV	0	0	
ddI	0	0	
d4T	0	0	
3TC	0	0	
ABC	0	0	
TDF	0	0	
NVP	0	0	
EFV	0	0	
ETR (***)	Susceptible		
RPV (***)	Susceptible		
SQV	27.51	9.492	48G 84V 73G 90L 11V 88N 47V 95C 53L 26T 71A 80T 54L 97L 24L
IDV	11.785	6.138	82V 54L 88N 29D 84V 73G 21E 65E 32I 46I 11V 71A 85I 30D 90L
NFV	22.547	6.771	88N 30D 84V 82V 97L 73G 68G 90L 71A 46I 31T 75V 54L 5L 89M
APV	232.142	16.885	33F 76L 50I 84V 32I 46I 54L 85I 47V 10F 22A 63P 13I 82V 89M
LPV	98.313	13.408	82V 50I 46I 84V 33F 76L 54L 22A 71A 10F 7Q 63P 24L 47V 25D
TPV	5.659	3.733	69H 46I 48G 84V 47V 89M 71A 72I 91T 15V 54L 90L 82V 88N 19L
DRV	65.381	11.052	47V 33F 84V 76L 43K 73G 71A 65E 10F 89M 48G 32I 93I 15I
ATV	42.544	10.965	48G 73G 84V 82V 4T 33F 71A 88N 90L 7Q 24L 76L 45K 32I 54L

(*) based on LIBSVM, Copyright (c) 2000, Chih-Chung Chang and Chih-Jen Lin

(**) Positions are ordered according to their impact on the phenotype prediction. Differences with respect to HXB2 are underlined. Positions shown in red and in green contribute to an increase or decrease in resistance, repectively. At most 15 positions are shown for each drug.

(***) Resistance predictions and scored mutations for ETR and RPV were performed with rules-based drug-resistance interpretation models by HIV-GRADE (http://www.hiv-grade.de)

geno2pheno®

phenotype prediction from genotype (version 3.4)

IV. Interpretation

Patient:	Birth date:	Sampling date:
Current therapy:		Viral load:

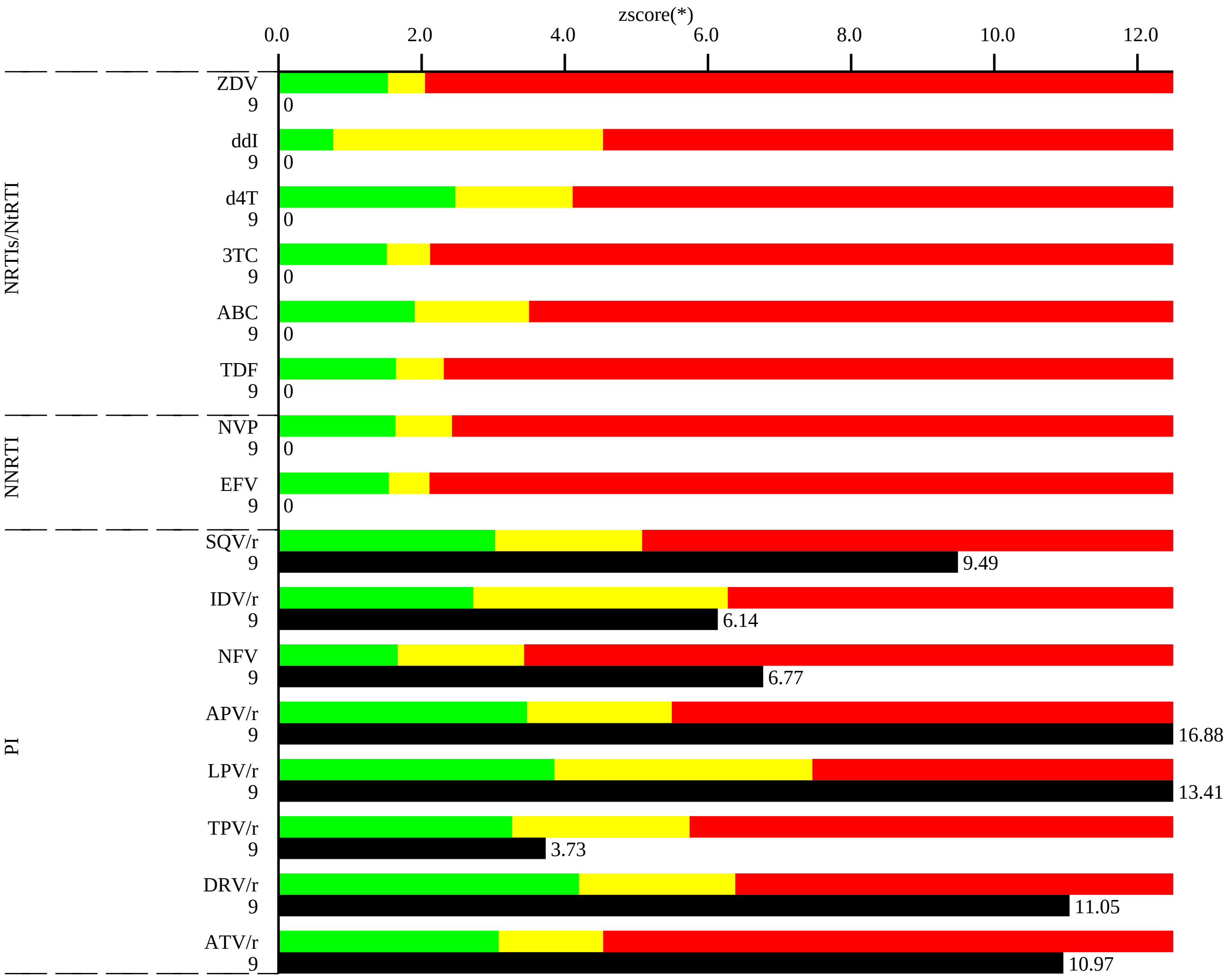

susceptible intermediate resistant

(*)number of standard deviations above mean of drug naive patients. Negative z-scores may indicate hypersusceptibility.

NRTIs/NtRTI:

NNRTIs:

PIs:

Previous genotypes:

date

signature
